# Supplementary material for: Impact of COVID-19 vaccination on symptoms and immune phenotypes in vaccine-naïve individuals with Long COVID
Source: Commun Med (Lond). 2025 May 9;5:163. doi: 10.1038/s43856-025-00829-3 (PMC12064684; doi:10.1038/s43856-025-00829-3)
Supplement: Supplementary file 2 — Supplementary Information [file 43856_2025_829_MOESM2_ESM.pdf]

Supplementary Figure 1: Number of symptoms per participant that resolved, improved, remained not an issue, remained an issue, or worsened after vaccination compared to before vaccination by measure of symptom severity.

The median number of symptoms per participant pre-vaccination was 23 (Q1-Q3 14.5-27, min-max 3-43). Change in symptom severity compared to pre-vaccination were assessed using two questions; physical effects associated with a symptom was assessed by the question, "While experiencing these symptoms, how much do/did they bother you in terms of discomfort or pain?" and social effects associated with a symptom was assessed by the question, "After quarantine, how much does/did the symptom impair your social or family functioning compared to pre-COVID?" in the 2-, 6-, and 12-week surveys. Changes in symptom severity were classified into mutually exclusive categories of resolved, improved, remained not an issue, remained an issue, or worsened. Resolved symptoms were defined as when participants reported that a symptom that affected them "very much", "quite a bit", "somewhat", or "a little bit" before vaccination affected them "not at all". Improved symptoms were defined as when participants reported that the symptom affected them less severely compared to pre-vaccination but not resolved (e.g., from "very much" pre-vaccination to "somewhat"). Symptoms that remained not an issue were defined as when participants reported that a symptom that affected them "not at all" before vaccination still affected them "not at all". Symptoms that remained an issue were defined as when participants reported the same level of severity as they did before vaccination, unless the same level was "not at all" (in which case the symptom would be "remained not an issue"). Worsened symptoms were defined as when participants reported that the symptom affected them more severely compared to pre-vaccination (e.g., from "somewhat" pre-vaccination to "very much"). Participants missing data for a symptom on a given survey and severity scale were not included in the respective plot; the number of participants included is show in the upper right corner of each plot.

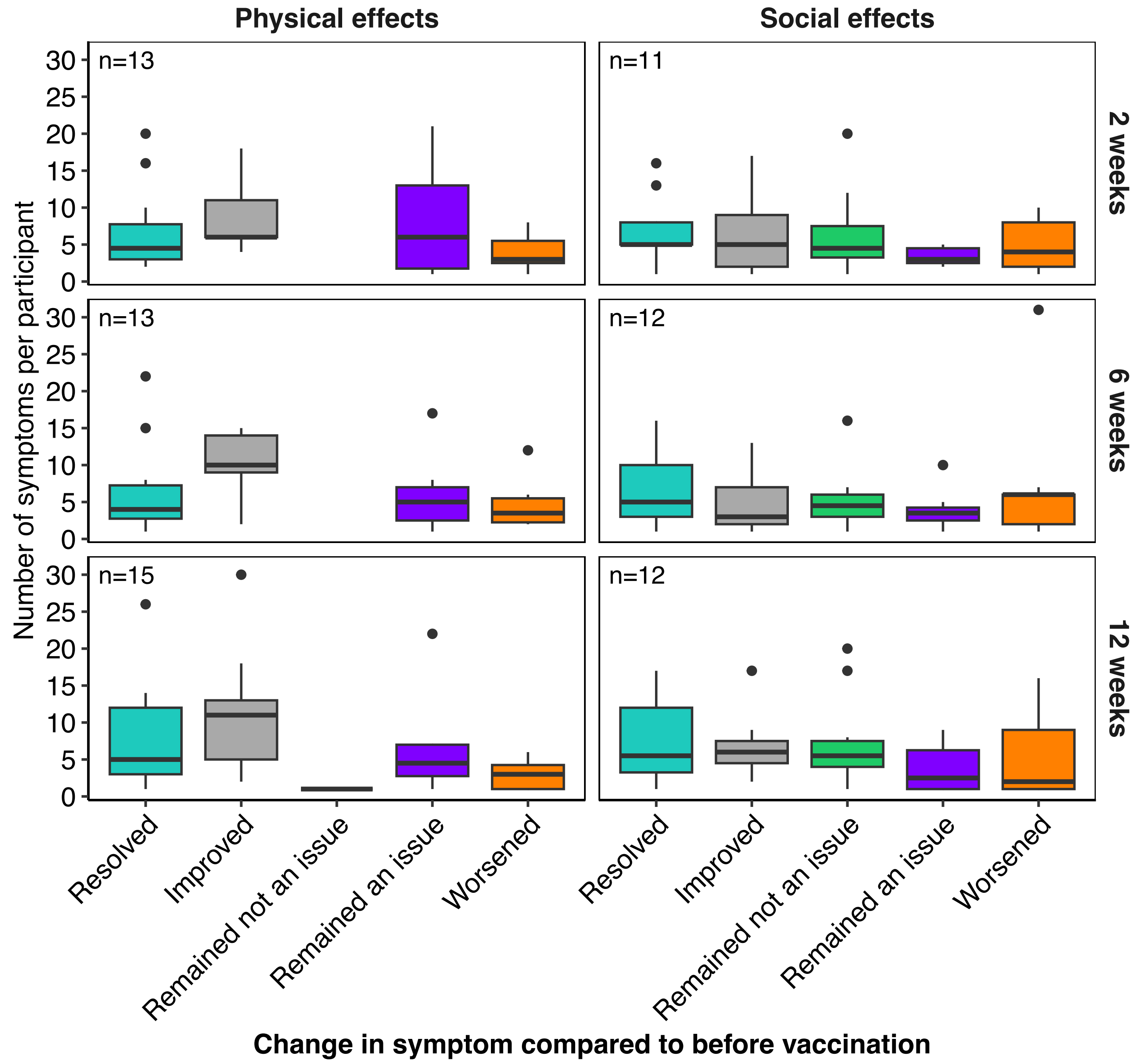

Physical effects
Social effects
n=13
n=11
n=13
n=12
n=15
n=12
2 weeks
6 weeks
12 weeks
Number of symptoms per participant
0
5
10
15
20
25
30
Resolved
Improved
Remained not an issue
Remained an issue
Worsened
Change in symptom compared to before vaccination

Supplementary Figure 2: Number of symptoms per participant that resolved, improved, remained not an issue, remained an issue, or worsened after vaccination compared to before vaccination by measure of symptom severity and overall health compared to before vaccination

Overall health at 12 weeks compared to before vaccination was reported as “better” (n=10), “same” (n=3), or “worse” (n=3). Categories of changes in symptom severity are defined in Supplementary Figure 1. Each dot represents the number of symptoms per participant in a given category of the change in symptom severity; participants with 0 symptoms in a category are not shown. Participants missing data for a symptom on a given survey and severity scale were not included in the respective plot; the number of participants included is show in the upper left corner of each plot.

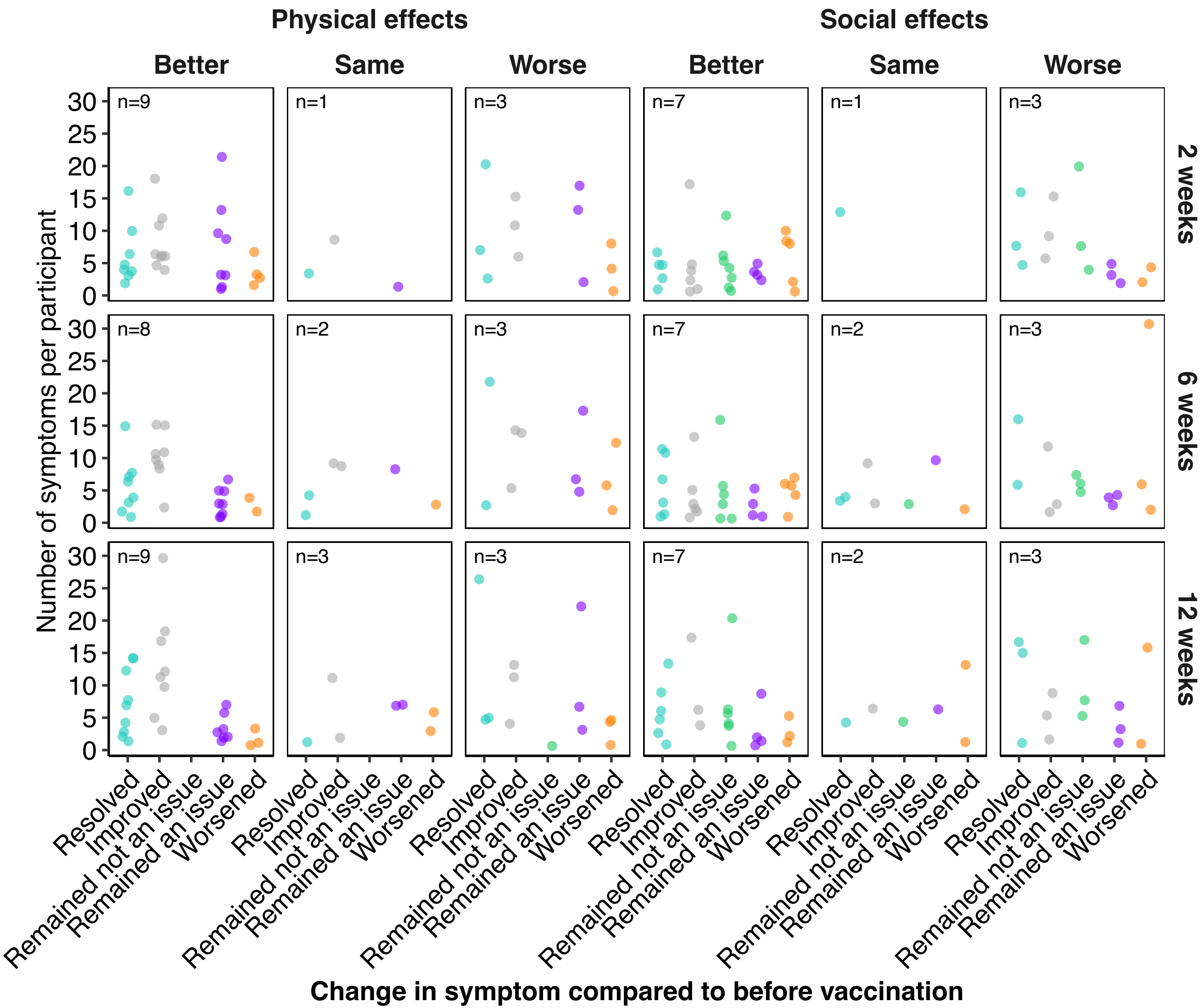

Physical effects
Social effects
Better
Same
Worse
2 weeks
6 weeks
12 weeks
Number of symptoms per participant
Change in symptom compared to before vaccination
Resolved
Improved
Remained not an issue
Remained an issue
Worsened
n=9
n=1
n=3
n=7
n=8
n=2

Supplementary Figure 3: Increased reactivity in pre-Omicron specific RBD epitopes post-vaccination.

a Line plots of matched anti-SARS-CoV-2 Beta variant reactivity scores against the Spike protein assessed by Rapid Extracellular Antigen Profiling (REAP), **b** Anti-SARS-CoV-2 Delta variant reactivity scores, **c** Anti-SARS-CoV-2 Omicron variant reactivity scores. Statistical significance determined by Wilcoxon with Bonferroni correction for multiple comparisons. Only significant values are mentioned in the graphs.

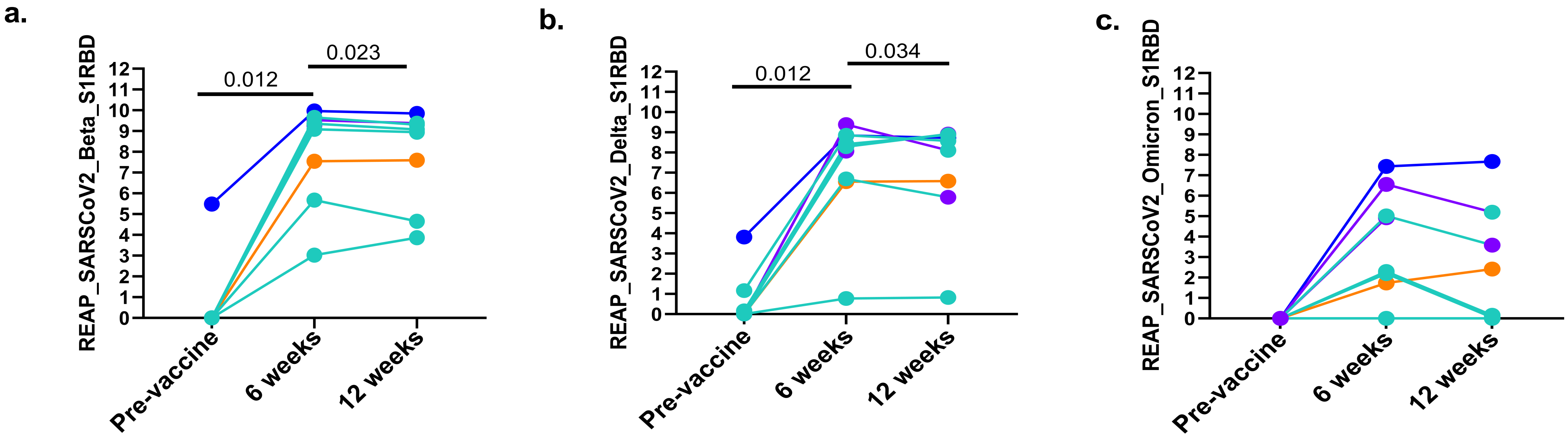

a.
REAP_SARSCoV2_Beta_S1RBD
0.012
0.023
Pre-vaccine
6 weeks
12 weeks
b.
REAP_SARSCoV2_Delta_S1RBD
0.012
0.034
Pre-vaccine
6 weeks
12 weeks
c.
REAP_SARSCoV2_Omicron_S1RBD
Pre-vaccine
6 weeks
12 weeks

Supplementary Figure 4: a REAP reactivities across the cohort at the first time point. Each column is one participant, grouped by cohort (B/W = transient). Each row represents one protein. Reactivities shown have at least one participant with a REAP score ≥1; **b** The number of autoantibody (aAb) reactivities per individual (ID) by group. Significance was assessed using Kruskal–Wallis tests, **c** Average REAP score delta for autoantibody reactivities per individual from the first to the final time point. Significance was assessed using Kruskal–Wallis tests. For the box plots, the central lines indicate the group median values, the top and bottom lines indicate the 75th and 25th percentiles, respectively, the whiskers represent 1.5× the interquartile range. Each dot represents one individual, **d** Autoantibody trajectory for one individual during vaccination. Each line represents one reactivity (red = SARS-CoV2, blue = Herpesvirus, black = autoantibody, **e** Overlay plot of all anti-viral and autoantibodies detected normalized to a starting REAP score of 0. Each line represents one reactivity.

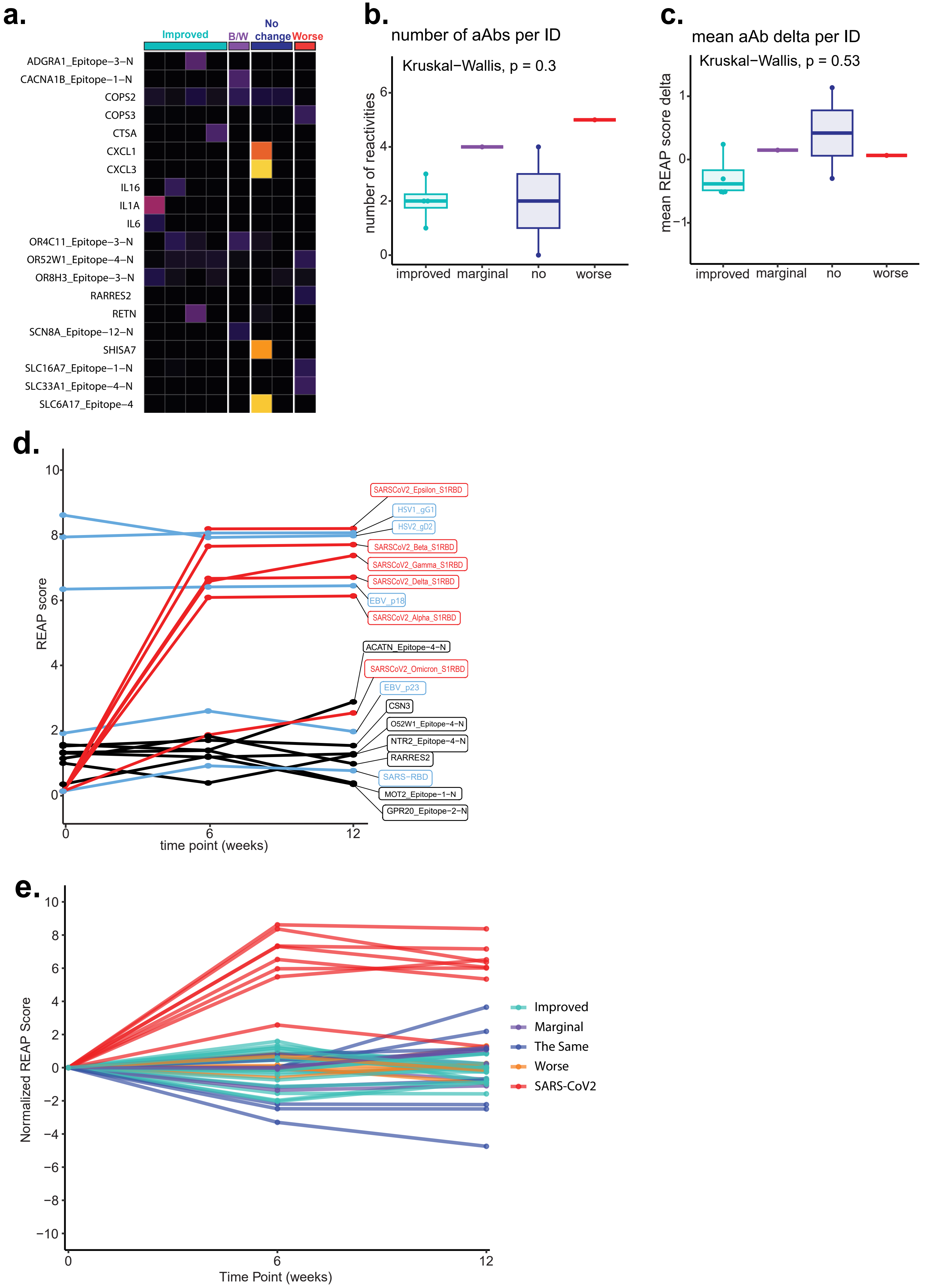
a.
Improved
B/W
No change
Worse
ADGRA1_Epitope−3−N
CACNA1B_Epitope−1−N
COPS2
COPS3
CTSA
CXCL1
CXCL3
IL16
IL1A
IL6
OR4C11_Epitope−3−N
OR52W1_Epitope−4−N
OR8H3_Epitope−3−N
RARRES2
RETN
SCN8A_Epitope−12−N
SHISA7
SLC16A7_Epitope−1−N
SLC33A1_Epitope−4−N
SLC6A17_Epitope−4
b.
number of aAbs per ID
Kruskal−Wallis, p = 0.3
number of reactivities
improved
marginal
no
worse
c.
mean aAb delta per ID
Kruskal−Wallis, p = 0.53
mean REAP score delta
improved
marginal
no
worse
d.
REAP score
time point (weeks)
SARSCoV2_Epsilon_S1RBD
HSV1_gG1
HSV2_gD2
SARSCoV2_Beta_S1RBD
SARSCoV2_Gamma_S1RBD
SARSCoV2_Delta_S1RBD
EBV_p18
SARSCoV2_Alpha_S1RBD
ACATN_Epitope−4−N
SARSCoV2_Omicron_S1RBD
EBV_p23
CSN3
O52W1_Epitope−4−N
NTR2_Epitope−4−N
RARRES2
SARS−RBD
MOT2_Epitope−1−N
GPR20_Epitope−2−N
e.
Normalized REAP Score
Time Point (weeks)
Improved
Marginal
The Same
Worse
SARS-CoV2

Supplementary Figure 5: Bar graph results of the bootstrapped (BS) PLS-DA analysis showing all features ranked from highest initial VIP score to lowest score. VIP Score Threshold of 0.8, representing the initial criteria for importance is shown as a solid line. Bar graphs represent the initial VIP threshold and whiskers represent the bias-corrected (BC) bootstrapped 95% confidence intervals (CIs) of the VIP score. Features were categorized as important if they met the initial threshold criteria (i.e. VIP score ≥ 0.8). Features were categorized as most significant and important if they met the following threshold criteria: an initial VIP score ≥ 0.8, AND whose VIP BSBCCI ≥ 0.8, AND whose regression coefficient BSBCCI did not span zero. The Threshold Criteria (Thrsh. Criteria) dot plot is shown below the bar graphs, demarcating the features passing each threshold parameter. Threshold parameters are defined as follows from lowest to highest: Below- features whose initial VIP score was below the threshold of importance; Initial- features whose initial VIP score passed importance but did not subsequently pass other bootstrapped threshold parameters. Regression Bootstrap (rBS)- features who passed the initial threshold criteria and the regression threshold criteria but did not pass the final bootstrapped VIP threshold criteria (i.e., VIP BC BS 95% CI fell below 0.8); Bootstrapped VIP (BS-VIP)- features that met the initial and Bootstrapped VIP threshold criteria but did not meet the regression threshold criteria; and Complete- features that met all criteria. For additional information on PLS-DA please see the Methods.

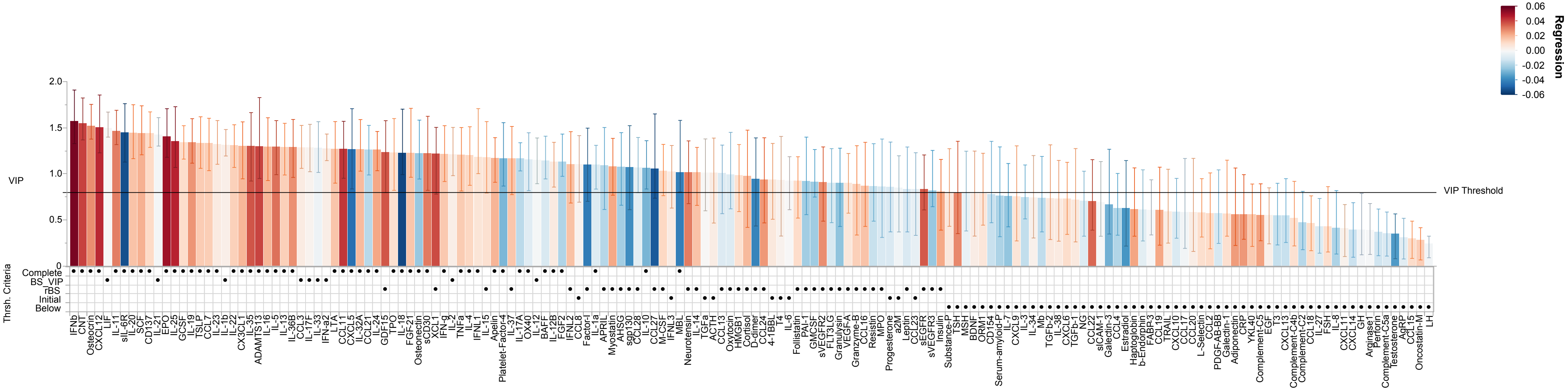
VIP
2.0
1.5
1.0
0.5
0
VIP Threshold
Regression
0.06
0.04
0.02
0.00
-0.02
-0.04
-0.06
Thrsh. Criteria
Complete
BS_VIP
rBS
Initial
Below
IFNb
CNT
Osteocrin
CXCL12
LIF
IL-11
sIL-6R
IL-20
SCF
CD137
IL-21
EPO
IL-25
GCSF
IL-19
TSLP
CCL7
IL-23
IL-1b
IL-22
CX3CL1
IL-35
ADAMTS13
IL-16
IL-5
IL-13
IL-36B
CCL3
IL-17F
IL-33
IFN-a2
LTA
CCL11
CXCL5
IL-32A
CCL21
IL-24
GDF15
TPO
IL-18
FGF-21
Osteonectin
sCD30
XCL1
IFN-g
IL-2
TNFa
IL-4
IFNL1
IL-15
Apelin
Platelet-Factor-4
IL-37
IL-17A
OX40
IL-12
BAFF
IL-12B
FGF2
IFNL2
CCL8
Factor-I
IL-1a
APRIL
Myostatin
AHSG
sgp130
CCL28
IL-10
CCL27
M-CSF
IFNL3
MBL
Neurotensin
IL-14
TGFa
ACTH
CCL13
Oxytocin
HMGB1
Cortisol
D-dimer
CCL24
4-1BBL
T4
IL-6
Follistatin
PAI-1
GMCSF
sVEGFR2
FLT3LG
Granulysin
VEGF-A
Granzyme-B
CCL16
Resistin
MPO
Progesterone
a2M
Leptin
CCL23
sEGFR
sVEGFR3
Insulin
Substance-P
TSH
MSH
BDNF
ORM1
CD154
Serum-amyloid-P
IL-7
CXCL9
IL-3
IL-34
Mb
TGFb-2
IL-38
CXCL6
TGFb-1
NG
CCL22
sICAM-1
Galectin-3
CCL4
Estradiol
Haptoglobin
b-Endorphin
FABP3
CCL19
TRAIL
CXCL10
CCL17
CCL20
L-Selectin
CCL2
PDGF-AB-BB
Galectin-1
Adiponectin
CRP
YKL40
Complement-C5
EGF
T3
CXCL13
Complement-C4b
Complement-C2
CCL18
IL-27
FSH
IL-8
CXCL11
CXCL14
GH
Arginase1
Perforin
Complement-C5a
Testosterone
AGRP
CCL15
Oncostatin-M
LH

Supplementary Table 1: Survey questions assessing the physical and social effects for each symptom prior to vaccination and at surveys sent 2, 6, and 12 weeks after vaccination.

**Measure of symptom severity**	**Survey questions**	**Likert scale response**
Physical	"While experiencing these symptoms, how much do/did they bother you in terms of discomfort or pain?"	Not at all = 0<br>A little bit = 1<br>Somewhat = 2<br>Quite a bit = 3<br>Very much = 4
Social	"After quarantine, how much does/did the symptom impair your social or family functioning compared to pre-COVID?"	

Supplementary Table 2: Median number of symptoms per participant reported as bothering participants "very much", "quite a bit", "somewhat", "a little bit", or "not at all" on the physical and social effects scales.

Weeks since first COVID-19 vaccine dose	n	Very much	Quite a bit	Somewhat	A little bit	Not at all
Physical effect scale						
Pre-vaccination	16	8 (4-12)	6 (5-9.5)	5 (3.5-7)	3 (1.25-4.75)	4.5 (2.75-6.25)
2 weeks	14	4.5 (1.75-9.25)	6 (3-9)	6 (3-7)	4 (3-6.75)	4 (3-7)
6 weeks	14	3 (3-5)	5 (2.25-6)	4 (3-6)	6 (5-8)	4 (3-7)
12 weeks	16	1.5 (1-3.25)	4.5 (3-5.25)	7 (1-11)	7 (3-9)	6.5 (3.25-11)
Social effect scale						
Pre-vaccination	16	5 (2-7)	3 (2-5.75)	5 (3-6.75)	3 (1-7)	5.5 (4-15.5)
2 weeks	14	1 (1-5)	2.5 (1.75-4.25)	4 (1-5)	5 (3-8.5)	11.5 (6.25-18.25)
6 weeks	14	5 (2-5)	3 (1.5-7)	5 (5-5)	4 (2-9)	8 (4.5-15.75)
12 weeks	16	3 (1-3)	3 (1-5)	3 (2.25-4.75)	6 (2-8)	13 (6.5-21)

Interquartile ranges are shown in parentheses. Physical effects were measured with the question "While experiencing these symptoms, how much do/did they bother you in terms of discomfort or pain?", and social effects were measured with the question, "After quarantine, how much does/did the symptom impair your social or family functioning compared to pre-COVID?" in the 2-, 6-, and 12-week surveys.

Supplementary Methods

Study surveys used to screen individuals for eligibility, document consent, collect demographic information, and collect information on symptoms and overall health before vaccination and 2, 6, and 12 weeks after vaccination.

Screening Questionnaire

Please complete the survey below to determine eligibility for the Yale COVID Recovery Study.

If you have any questions about the survey, please email covidrecovery@yale.edu.

Thank you for your interest!

Please enter your first name.	__________________________
Please enter your last name.	__________________________
Please provide an email address that we may use to contact you.	__________________________
What is your birthdate?	__________________________
What is a phone number that we may use to reach you?	__________________________
Are you currently 18 years or older?	◯ Yes<br>◯ No
Have you ever tested positive for COVID-19? (The type of test may have been PCR, antigen, antibody, or T-test.)	◯ Yes<br>◯ No
Have you ever been hospitalized due to COVID-19?	◯ Yes<br>◯ No
Have you ever been diagnosed with COVID-19 by a clinician?	◯ Yes<br>◯ No
In your opinion, do you have symptoms more than 2 months after your initial COVID-19 infection (long covid) that interfere with your quality of life or with your ability to do your normal activities?	◯ Yes<br>◯ No
Have you received any dose(s) of a COVID-19 vaccine?	◯ Yes<br>◯ No
Do you plan to get the COVID-19 vaccine? (Note: You do not need to currently have a vaccine appointment to be planning to get the vaccine.)	◯ Yes<br>◯ No
Are you willing to give a blood and saliva sample 3 times at your home on a Monday or Tuesday morning (before 1 pm)?	◯ Yes<br>◯ No
Has consent been completed and verified?	☐ Yes<br>☐ No

Consent Verification

Please complete the survey below.

Thank you!

15) Study Coordinator First Name __________________________________

16) Study Coordinator Last Name __________________________________

17) Participant First Name __________________________________

18) Participant Last Name __________________________________

19) Has the participant consented and has the consent been verified?
- ◯ Yes
- ◯ No

Survey 1

Thank you for taking Survey 1 for the Yale COVID Recovery Study!

Please remember that you may stop this survey and come back to it at any point.

You may also find it helpful to have the below items ready as you complete the survey. If you do not have these items, please still fill in the information as best you can.

Testing results: dates, type (PCR, antigen) and result of tests. If you do not remember the exact date, the estimated date is enough.Symptom time and severity: your symptom log. You will be asked to pick symptoms you have had since you began having COVID-19 symptoms.

There are 3 sections in this survey. In this section, we will ask for your contact information so that we may follow up with future research opportunities. We will also ask demographic questions so that we can understand how Long COVID is affecting the population.

What is your birthdate?

____

What is your zip code?

____

Do you plan on getting the vaccine?

- ◯ Yes, and I have an appointment scheduled
- ◯ Yes, but I don't have an appointment yet
- ◯ I'm not planning on getting the vaccine
- ◯ I have already gotten at least one dose of the COVID-19 vaccine

When is your vaccine appointment?

____

What is your gender?

- ◯ Male
- ◯ Female
- ◯ Not listed ______

Please specify (optional):

____

What race(s) or ethnicity do you consider yourself to be?
*Please choose 1 or more

- ☐ American Indian/Alaska Native
- ☐ Asian ______
- ☐ Black or African American
- ☐ Hispanic or Latino ______
- ☐ Native Hawaiian/Other Pacific Islander ______
- ☐ White
- ☐ Some other race ______

Please specify your race and/or ethnicity

____

Please specify (select as many as apply to you)

- ☐ Chinese
- ☐ Filipino
- ☐ Asian Indian
- ☐ Vietnamese
- ☐ Korean
- ☐ Japanese
- ☐ Other Asian (e.g., Pakistani, Cambodian, Hmong)

Please specify (select as many as apply to you):

- ☐ Native Hawaiian
- ☐ Samoan
- ☐ Chamorro
- ☐ Other Pacific Islander (e.g., Tongan, Fijian, Mashallese)

Please specify (select as many as apply to you)

- ☐ Mexican/Mexican American
- ☐ Puerto Rican
- ☐ Dominican (Republic)
- ☐ Cuban/Cuban American
- ☐ Central or South American
- ☐ Other Latino/Hispanic

Have you ever been told by a doctor that you have any of the following?

- ☐ Any allergies
- ☐ Arthritis (including rheumatoid arthritis, gout, lupus, or fibromyalgia)
- ☐ Asthma
- ☐ Blood disorder (including sickle cell disease or Thalassemia)
- ☐ Cancer or malignancy of any kind
- ☐ Cerebrovascular conditions affecting blood vessels to or in the brain (including stroke)
- ☐ Chronic lung disease (including emphysema, chronic bronchitis, chronic obstructive pulmonary disease (COPD), or pulmonary fibrosis)
- ☐ Cystic fibrosis
- ☐ Diabetes
- ☐ Gastrointestinal issues (including IBS or acid reflux)
- ☐ Heart attack, also called myocardial infarction
- ☐ Heart conditions (including coronary artery disease or cardiomyopathies)
- ☐ Heart failure
- ☐ High cholesterol
- ☐ History of organ transplant (including kidney, liver, heart, or lung)
- ☐ Hypertension or high blood pressure
- ☐ Immunocompromised state (including weakened immun system from blood or bone marrow transplant, immune deficiencies, HIV, use of corticosteroids, or use of other immune-weakening medicines)
- ☐ Kidney disease
- ☐ Liver disease
- ☐ Mental health conditions (including anxiety, depression, bipolar disorder, schizophrenia, obsessive or compulsive disorder)
- ☐ Neurologic conditions (including seizures or dementia)
- ☐ Spinal disorder(s)
- ☐ Other

Please describe "Other": ______________________________

Now we are going to ask you questions about your experience with COVID-19. Please answer as best you can, and we understand that dates may not be exact. You have 2 survey sections to go.

When did your symptoms due to COVID-19 begin? __________________________________

Have you been tested for COVID-19 at least once?
- ◯ Yes
- ◯ No

What was the date of the first time you were tested for COVID-19? __________________________________

What was the type of test?
- ◯ PCR test
- ◯ Antigen test
- ◯ Not sure

What was the result?
- ◯ Positive
- ◯ Negative
- ◯ Inconclusive
- ◯ Awaiting result

Were you tested for COVID more than once?
- ◯ Yes
- ◯ No

How many times were you tested for COVID-19? __________________________________

What was the date of your second COVID-19 test? __________________________________

What was the result of your second test?
- ◯ Positive
- ◯ Negative
- ◯ Inconclusive
- ◯ Awaiting result

What was the date of your most recent COVID-19 test? __________________________________

What was the result of your most recent COVID-19 test?
- ◯ Positive
- ◯ Negative
- ◯ Inconclusive
- ◯ Awaiting result

If you ever tested positive for COVID-19, when was your most recent positive test? __________________________________

Do you believe that you were reinfected with COVID-19?
- ◯ Yes
- ◯ No

Were you ever hospitalized due to COVID-19?
- ◯ Yes
- ◯ No

Have you visited the hospital or been hospitalized for COVID-19 symptoms that began more than two weeks after you first got sick?
- ◯ Yes
- ◯ No

Which of the following symptoms have you experienced due to COVID-19? Select as many as apply. As a reminder, you stated that your symptoms began on [s1_symptom_date]. *select all that apply

Symptoms

Have you experienced this symptom (at any time)?

Approximately when did you begin experiencing this symptom?

Does/did this symptom come and go?

Has this symptom gone away completely?

Abdominal pain

______

______

______

______

Abnormally low temperature

______

______

______

______

Acid reflux

______

______

______

______

Afternoon or evening fevers/low-grade fevers

______

______

______

______

Anemia (low number of red blood cells)

______

______

______

Anxiety

______

______

______

______

Arrythmia (improper beating of the heart due to electrical impulse problems)

______

______

______

______

Bilateral neck throbbing around lymph nodes

______

______

______

______

Blurry vision

______

______

______

______

Bone aches in extremities

______

______

______

______

Brain fog

______

______

______

______

Brain pressure

______

______

______

______

Bulging veins

______

______

______

______

Burning sensations

______

______

______

______

Bruising of skin

______

______

______

______

Calf cramps

______

______

______

______

Change in nails (i.e. white spots, brittleness, change in moons)

______

______

______

______

Changes in voice

______

______

______

______

Changed sense of taste

______

______

______

______

Chills but no fever

______

______

______

______

Clogged ears

______

______

______

______

Cold burning feeling in lungs

______

______

______

______

Confusion

______

______

______

______

Congested or runny nose

______

______

______

______

Constant thirst

______

______

______

______

Costochondritis (inflammation of the cartilage that connects a rib to the breastbone)

______

______

______

______

Cough

______

______

______

______

Coughing up blood

______

______

______

______

Covid toes (tender or itchy rash or chilblains on the toes or foot)

______

______

______

______

Cracked or dry lips

______

______

______

______

Dental problems (i.e. chipped tooth, tooth loss)

______

______

______

______

Diarrhea

______

______

______

______

Difficulty concentrating or focusing

______

______

______

______

Difficulty sleeping

______

______

______

______

Difficulty speaking properly

______

______

______

______

Discoloration of the skin (for example: purple or blue on the hands or feet, no blistering)

______

______

______

______

Dizziness

______

______

______

______

Dry eyes

______

______

______

______

Dry or peeling skin

______

______

______

______

Dry scalp or dandruff

______

______

______

______

Dry throat

______

______

______

______

Ear pain/earache

______

______

______

______

Elevated thyroid hormones

______

______

______

______

Extreme pressure at base of head or occipital nerve

______

______

______

______

Eye stye or infection

______

______

______

______

Fatigue

______

______

______

______

Feeling irritable

______

______

______

______

Feeling of burning skin

______

______

______

______

Fever or chills

______

______

______

______

Floaters or flashes of light in vision

______

______

______

______

Foot pain

______

______

______

______

GERD (acid reflux) with excessive salivation

______

______

______

______

Goiter or lump in throat

______

______

______

______

Hair loss

______

______

______

______

Hand or wrist pain

______

______

______

______

Headache

______

______

______

______

Heart palpitations (heart skipping a beat or racing)

______

______

______

______

Heat intolerance

______

______

______

______

High blood pressure

______

______

______

______

Hormone imbalances

______

______

______

______

“Hot” blood rush

______

______

______

______

Inability to cry

______

______

______

______

Inability to exercise or be active

______

______

______

______

Inability to yawn

______

______

______

______

Internal tremors or buzzing/vibration

______

______

______

______

Irregular or skipped menstrual cycles

______

______

______

______

Jaw pain

______

______

______

______

Joint pain

______

______

______

______

Kidney issues or protein in urine

______

______

______

______

Kidney pain

______

______

______

______

Low blood oxygen

______

______

______

______

Low blood pressure

______

______

______

______

Lower back pain

______

______

______

______

Loss of appetite

______

______

______

______

Loss of hearing

______

______

______

______

Loss or decrease in quality of vision

______

______

______

______

Lump in throat/difficulty swallowing

______

______

______

______

Memory problems

______

______

______

______

Menstrual cycles that are heavier or lighter than normal

______

______

______

______

Mid-back pain at base of ribs

______

______

______

______

Mouth sores or sore tongue

______

______

______

______

Muscle or body aches

______

______

______

______

Muscle twitching

______

______

______

______

Nausea or vomiting

______

______

______

______

Neck muscle pain

______

______

______

______

Nerve pain

______

______

______

______

Nerve sensations (tingling, pins and needles, numbness)

______

______

______

______

Neuropathy in feet and hands (weakness, numbness, and pain)

______

______

______

______

New allergies

______

______

______

______

Night sweats

______

______

______

______

Nightmares

______

______

______

______

Painful scalp

______

______

______

______

Partial or complete loss of sense of smell

______

______

______

______

Partial or complete loss of sense of taste

______

______

______

______

Persistent chest pain or pressure

______

______

______

______

Personality change (drastic)

______

______

______

______

Petechiae (pinpoint rash)

______

______

______

______

Phantom smells

______

______

______

______

Phlegm in back of throat

______

______

______

______

Post-exertional malaise (worsened symptoms or flu-like symptoms after exertion)

______

______

______

______

Postnasal drip

______

______

______

______

Rash

______

______

______

______

Reflux or heartburn

______

______

______

______

Runny nose

______

______

______

______

Sadness

______

______

______

______

Seizures

______

______

______

______

Sharp or sudden chest pain

______

______

______

______

Shortness of breath or difficulty breathing

______

______

______

______

Shortness of breath or exhaustion from bending over

______

______

______

______

Sleeping more than normal

______

______

______

______

Sore throat

______

______

______

______

Spinal issues

______

______

______

______

Spikes in blood pressure

______

______

______

______

Swollen hands or feet

______

______

______

______

Swollen lymph nodes

______

______

______

______

Syncope (fainting)

______

______

______

______

Tachycardia (rapid heartbeat) at rest

______

______

______

______

Tachycardia (rapid heartbeat) after standing up

______

______

______

______

Thrush (white fungal infection in the mouth or throat)

______

______

______

______

Tinnitus or humming in ears

______

______

______

______

Tremors or shakiness

______

______

______

______

Upper back pain

______

______

______

______

UTI (urinary tract infection)

______

______

______

______

Weakened neck

______

______

______

______

Weight gain

______

______

______

______

Other symptom(s)

______

Please specify "Other symptoms"	______________________________
Have you experienced this symptom (at any time)?	☐ Yes
When did the symptom begin?	______________________________
Does/Did this symptom come and go?	◯ Yes<br>◯ No
Has the symptom gone away completely?	◯ Yes<br>◯ No
Have you experienced this symptom (at any time)?	☐ Yes

When did the symptom begin?	__________________________
Does/Did this symptom come and go?	◯ Yes<br>◯ No
Has the symptom gone away completely?	◯ Yes<br>◯ No
Have you experienced this symptom (at any time)?	☐ Yes
When did the symptom begin?	__________________________
Does/Did this symptom come and go?	◯ Yes<br>◯ No
Has the symptom gone away completely?	◯ Yes<br>◯ No
Have you experienced this symptom (at any time)?	☐ Yes
When did the symptom begin?	__________________________
Does/Did this symptom come and go?	◯ Yes<br>◯ No
Has the symptom gone away completely?	◯ Yes<br>◯ No
Have you experienced this symptom (at any time)?	☐ Yes
When did the symptom begin?	__________________________
Does/Did this symptom come and go?	◯ Yes<br>◯ No
Has the symptom gone away completely?	◯ Yes<br>◯ No
Have you experienced this symptom (at any time)?	☐ Yes
When did the symptom begin?	__________________________
Does/Did this symptom come and go?	◯ Yes<br>◯ No
Has the symptom gone away completely?	◯ Yes<br>◯ No
Have you experienced this symptom (at any time)?	☐ Yes

When did the symptom begin?	__________________________________
Does/Did this symptom come and go?	◯ Yes<br>◯ No
Has the symptom gone away completely?	◯ Yes<br>◯ No
Have you experienced this symptom (at any time)?	☐ Yes
When did the symptom begin?	__________________________________
Does/Did this symptom come and go?	◯ Yes<br>◯ No
Has the symptom gone away completely?	◯ Yes<br>◯ No
Have you experienced this symptom (at any time)?	☐ Yes
When did the symptom begin?	__________________________________
Does/Did this symptom come and go?	◯ Yes<br>◯ No
Has the symptom gone away completely?	◯ Yes<br>◯ No
Have you experienced this symptom (at any time)?	☐ Yes
When did the symptom begin?	__________________________________
Does/Did this symptom come and go?	◯ Yes<br>◯ No
Has the symptom gone away completely?	◯ Yes<br>◯ No
Have you experienced this symptom (at any time)?	☐ Yes
When did the symptom begin?	__________________________________
Does/Did this symptom come and go?	◯ Yes<br>◯ No
Has the symptom gone away completely?	◯ Yes<br>◯ No
Have you experienced this symptom (at any time)?	☐ Yes

When did the symptom begin?	__________________________
Does/Did this symptom come and go?	◯ Yes<br>◯ No
Has the symptom gone away completely?	◯ Yes<br>◯ No
Have you experienced this symptom (at any time)?	☐ Yes
When did the symptom begin?	__________________________
Does/Did this symptom come and go?	◯ Yes<br>◯ No
Has the symptom gone away completely?	◯ Yes<br>◯ No
Have you experienced this symptom (at any time)?	☐ Yes
When did the symptom begin?	__________________________
Does/Did this symptom come and go?	◯ Yes<br>◯ No
Has the symptom gone away completely?	◯ Yes<br>◯ No
Have you experienced this symptom (at any time)?	☐ Yes
When did the symptom begin?	__________________________
Does/Did this symptom come and go?	◯ Yes<br>◯ No
Has the symptom gone away completely?	◯ Yes<br>◯ No
Have you experienced this symptom (at any time)?	☐ Yes
When did the symptom begin?	__________________________
Does/Did this symptom come and go?	◯ Yes<br>◯ No
Has the symptom gone away completely?	◯ Yes<br>◯ No
Have you experienced this symptom (at any time)?	☐ Yes

When did the symptom begin?	______________________
Does/Did this symptom come and go?	◯ Yes<br>◯ No
Has the symptom gone away completely?	◯ Yes<br>◯ No
Have you experienced this symptom (at any time)?	☐ Yes
When did the symptom begin?	______________________
Does/Did this symptom come and go?	◯ Yes<br>◯ No
Has the symptom gone away completely?	◯ Yes<br>◯ No
Have you experienced this symptom (at any time)?	☐ Yes
When did the symptom begin?	______________________
Does/Did this symptom come and go?	◯ Yes<br>◯ No
Has the symptom gone away completely?	◯ Yes<br>◯ No
Have you experienced this symptom (at any time)?	☐ Yes
When did the symptom begin?	______________________
Does/Did this symptom come and go?	◯ Yes<br>◯ No
Has the symptom gone away completely?	◯ Yes<br>◯ No
Have you experienced this symptom (at any time)?	☐ Yes
When did the symptom begin?	______________________
Does/Did this symptom come and go?	◯ Yes<br>◯ No
Has the symptom gone away completely?	◯ Yes<br>◯ No
Have you experienced this symptom (at any time)?	☐ Yes

When did the symptom begin?	__________________________________
Does/Did this symptom come and go?	◯ Yes<br>◯ No
Has the symptom gone away completely?	◯ Yes<br>◯ No
Have you experienced this symptom (at any time)?	☐ Yes
When did the symptom begin?	__________________________________
Does/Did this symptom come and go?	◯ Yes<br>◯ No
Has the symptom gone away completely?	◯ Yes<br>◯ No
Have you experienced this symptom (at any time)?	☐ Yes
When did the symptom begin?	__________________________________
Does/Did this symptom come and go?	◯ Yes<br>◯ No
Has the symptom gone away completely?	◯ Yes<br>◯ No
Have you experienced this symptom (at any time)?	☐ Yes
When did the symptom begin?	__________________________________
Does/Did this symptom come and go?	◯ Yes<br>◯ No
Has the symptom gone away completely?	◯ Yes<br>◯ No
Have you experienced this symptom (at any time)?	☐ Yes
When did the symptom begin?	__________________________________
Does/Did this symptom come and go?	◯ Yes<br>◯ No
Has the symptom gone away completely?	◯ Yes<br>◯ No
Have you experienced this symptom (at any time)?	☐ Yes

When did the symptom begin?	__________________________
Does/Did this symptom come and go?	◯ Yes<br>◯ No
Has the symptom gone away completely?	◯ Yes<br>◯ No
Have you experienced this symptom (at any time)?	☐ Yes
When did the symptom begin?	__________________________
Does/Did this symptom come and go?	◯ Yes<br>◯ No
Has the symptom gone away completely?	◯ Yes<br>◯ No
Have you experienced this symptom (at any time)?	☐ Yes
When did the symptom begin?	__________________________
Does/Did this symptom come and go?	◯ Yes<br>◯ No
Has the symptom gone away completely?	◯ Yes<br>◯ No
Have you experienced this symptom (at any time)?	☐ Yes
When did the symptom begin?	__________________________
Does/Did this symptom come and go?	◯ Yes<br>◯ No
Has the symptom gone away completely?	◯ Yes<br>◯ No
Have you experienced this symptom (at any time)?	☐ Yes
When did the symptom begin?	__________________________
Does/Did this symptom come and go?	◯ Yes<br>◯ No
Has the symptom gone away completely?	◯ Yes<br>◯ No
Have you experienced this symptom (at any time)?	☐ Yes

When did the symptom begin?	________________________________
Does/Did this symptom come and go?	◯ Yes<br>◯ No
Has the symptom gone away completely?	◯ Yes<br>◯ No
Have you experienced this symptom (at any time)?	☐ Yes
When did the symptom begin?	________________________________
Does/Did this symptom come and go?	◯ Yes<br>◯ No
Has the symptom gone away completely?	◯ Yes<br>◯ No
Have you experienced this symptom (at any time)?	☐ Yes
When did the symptom begin?	________________________________
Does/Did this symptom come and go?	◯ Yes<br>◯ No
Has the symptom gone away completely?	◯ Yes<br>◯ No
Have you experienced this symptom (at any time)?	☐ Yes
When did the symptom begin?	________________________________
Does/Did this symptom come and go?	◯ Yes<br>◯ No
Has the symptom gone away completely?	◯ Yes<br>◯ No
Have you experienced this symptom (at any time)?	☐ Yes
When did the symptom begin?	________________________________
Does/Did this symptom come and go?	◯ Yes<br>◯ No
Has the symptom gone away completely?	◯ Yes<br>◯ No
Have you experienced this symptom (at any time)?	☐ Yes

When did the symptom begin?	__________________
Does/Did this symptom come and go?	◯ Yes<br>◯ No
Has the symptom gone away completely?	◯ Yes<br>◯ No
Have you experienced this symptom (at any time)?	☐ Yes
When did the symptom begin?	__________________
Does/Did this symptom come and go?	◯ Yes<br>◯ No
Has the symptom gone away completely?	◯ Yes<br>◯ No
Have you experienced this symptom (at any time)?	☐ Yes
When did the symptom begin?	__________________
Does/Did this symptom come and go?	◯ Yes<br>◯ No
Has the symptom gone away completely?	◯ Yes<br>◯ No
Have you experienced this symptom (at any time)?	☐ Yes
When did the symptom begin?	__________________
Does/Did this symptom come and go?	◯ Yes<br>◯ No
Has the symptom gone away completely?	◯ Yes<br>◯ No
Have you experienced this symptom (at any time)?	☐ Yes
When did the symptom begin?	__________________
Does/Did this symptom come and go?	◯ Yes<br>◯ No
Has the symptom gone away completely?	◯ Yes<br>◯ No
Have you experienced this symptom (at any time)?	☐ Yes

When did the symptom begin?	______________________
Does/Did this symptom come and go?	◯ Yes<br>◯ No
Has the symptom gone away completely?	◯ Yes<br>◯ No
Have you experienced this symptom (at any time)?	☐ Yes
When did the symptom begin?	______________________
Does/Did this symptom come and go?	◯ Yes<br>◯ No
Has the symptom gone away completely?	◯ Yes<br>◯ No
Have you experienced this symptom (at any time)?	☐ Yes
When did the symptom begin?	______________________
Does/Did this symptom come and go?	◯ Yes<br>◯ No
Has the symptom gone away completely?	◯ Yes<br>◯ No
Have you experienced this symptom (at any time)?	☐ Yes
When did the symptom begin?	______________________
Does/Did this symptom come and go?	◯ Yes<br>◯ No
Has the symptom gone away completely?	◯ Yes<br>◯ No
Have you experienced this symptom (at any time)?	☐ Yes
When did the symptom begin?	______________________
Does/Did this symptom come and go?	◯ Yes<br>◯ No
Has the symptom gone away completely?	◯ Yes<br>◯ No
Have you experienced this symptom (at any time)?	☐ Yes

When did the symptom begin?	__________________
Does/Did this symptom come and go?	◯ Yes<br>◯ No
Has the symptom gone away completely?	◯ Yes<br>◯ No
Have you experienced this symptom (at any time)?	☐ Yes
When did the symptom begin?	__________________
Does/Did this symptom come and go?	◯ Yes<br>◯ No
Has the symptom gone away completely?	◯ Yes<br>◯ No
Have you experienced this symptom (at any time)?	☐ Yes
When did the symptom begin?	__________________
Does/Did this symptom come and go?	◯ Yes<br>◯ No
Has the symptom gone away completely?	◯ Yes<br>◯ No
Have you experienced this symptom (at any time)?	☐ Yes
When did the symptom begin?	__________________
Does/Did this symptom come and go?	◯ Yes<br>◯ No
Has the symptom gone away completely?	◯ Yes<br>◯ No
Have you experienced this symptom (at any time)?	☐ Yes
When did the symptom begin?	__________________
Does/Did this symptom come and go?	◯ Yes<br>◯ No
Has the symptom gone away completely?	◯ Yes<br>◯ No
Have you experienced this symptom (at any time)?	☐ Yes

When did the symptom begin?	__________________________________
Does/Did this symptom come and go?	◯ Yes<br>◯ No
Has the symptom gone away completely?	◯ Yes<br>◯ No
Have you experienced this symptom (at any time)?	☐ Yes
When did the symptom begin?	__________________________________
Does/Did this symptom come and go?	◯ Yes<br>◯ No
Has the symptom gone away completely?	◯ Yes<br>◯ No
Have you experienced this symptom (at any time)?	☐ Yes
When did the symptom begin?	__________________________________
Does/Did this symptom come and go?	◯ Yes<br>◯ No
Has the symptom gone away completely?	◯ Yes<br>◯ No
Have you experienced this symptom (at any time)?	☐ Yes
When did the symptom begin?	__________________________________
Does/Did this symptom come and go?	◯ Yes<br>◯ No
Has the symptom gone away completely?	◯ Yes<br>◯ No
Have you experienced this symptom (at any time)?	☐ Yes
When did the symptom begin?	__________________________________
Does/Did this symptom come and go?	◯ Yes<br>◯ No
Has the symptom gone away completely?	◯ Yes<br>◯ No
Have you experienced this symptom (at any time)?	☐ Yes

When did the symptom begin?	__________________
Does/Did this symptom come and go?	◯ Yes<br>◯ No
Has the symptom gone away completely?	◯ Yes<br>◯ No
Have you experienced this symptom (at any time)?	☐ Yes
When did the symptom begin?	__________________
Does/Did this symptom come and go?	◯ Yes<br>◯ No
Has the symptom gone away completely?	◯ Yes<br>◯ No
Have you experienced this symptom (at any time)?	☐ Yes
When did the symptom begin?	__________________
Does/Did this symptom come and go?	◯ Yes<br>◯ No
Has the symptom gone away completely?	◯ Yes<br>◯ No
Have you experienced this symptom (at any time)?	☐ Yes
When did the symptom begin?	__________________
Does/Did this symptom come and go?	◯ Yes<br>◯ No
Has the symptom gone away completely?	◯ Yes<br>◯ No
Have you experienced this symptom (at any time)?	☐ Yes
When did the symptom begin?	__________________
Does/Did this symptom come and go?	◯ Yes<br>◯ No
Has the symptom gone away completely?	◯ Yes<br>◯ No
Have you experienced this symptom (at any time)?	☐ Yes

When did the symptom begin?	______________________________
Does/Did this symptom come and go?	◯ Yes<br>◯ No
Has the symptom gone away completely?	◯ Yes<br>◯ No
Have you experienced this symptom (at any time)?	☐ Yes
When did the symptom begin?	______________________________
Does/Did this symptom come and go?	◯ Yes<br>◯ No
Has the symptom gone away completely?	◯ Yes<br>◯ No
Have you experienced this symptom (at any time)?	☐ Yes
When did the symptom begin?	______________________________
Does/Did this symptom come and go?	◯ Yes<br>◯ No
Has the symptom gone away completely?	◯ Yes<br>◯ No
Have you experienced this symptom (at any time)?	☐ Yes
When did the symptom begin?	______________________________
Does/Did this symptom come and go?	◯ Yes<br>◯ No
Has the symptom gone away completely?	◯ Yes<br>◯ No
Have you experienced this symptom (at any time)?	☐ Yes
When did the symptom begin?	______________________________
Does/Did this symptom come and go?	◯ Yes<br>◯ No
Has the symptom gone away completely?	◯ Yes<br>◯ No
Have you experienced this symptom (at any time)?	☐ Yes

When did the symptom begin?	________________________________
Does/Did this symptom come and go?	◯ Yes<br>◯ No
Has the symptom gone away completely?	◯ Yes<br>◯ No
Have you experienced this symptom (at any time)?	☐ Yes
When did the symptom begin?	________________________________
Does/Did this symptom come and go?	◯ Yes<br>◯ No
Has the symptom gone away completely?	◯ Yes<br>◯ No
Have you experienced this symptom (at any time)?	☐ Yes
When did the symptom begin?	________________________________
Does/Did this symptom come and go?	◯ Yes<br>◯ No
Has the symptom gone away completely?	◯ Yes<br>◯ No
Have you experienced this symptom (at any time)?	☐ Yes
When did the symptom begin?	________________________________
Does/Did this symptom come and go?	◯ Yes<br>◯ No
Has the symptom gone away completely?	◯ Yes<br>◯ No
Have you experienced this symptom (at any time)?	☐ Yes
When did the symptom begin?	________________________________
Does/Did this symptom come and go?	◯ Yes<br>◯ No
Has the symptom gone away completely?	◯ Yes<br>◯ No
Have you experienced this symptom (at any time)?	☐ Yes

When did the symptom begin?	__________________________
Does/Did this symptom come and go?	◯ Yes<br>◯ No
Has the symptom gone away completely?	◯ Yes<br>◯ No
Have you experienced this symptom (at any time)?	☐ Yes
When did the symptom begin?	__________________________
Does/Did this symptom come and go?	◯ Yes<br>◯ No
Has the symptom gone away completely?	◯ Yes<br>◯ No
Have you experienced this symptom (at any time)?	☐ Yes
When did the symptom begin?	__________________________
Does/Did this symptom come and go?	◯ Yes<br>◯ No
Has the symptom gone away completely?	◯ Yes<br>◯ No
Have you experienced this symptom (at any time)?	☐ Yes
When did the symptom begin?	__________________________
Does/Did this symptom come and go?	◯ Yes<br>◯ No
Has the symptom gone away completely?	◯ Yes<br>◯ No
Have you experienced this symptom (at any time)?	☐ Yes
When did the symptom begin?	__________________________
Does/Did this symptom come and go?	◯ Yes<br>◯ No
Has the symptom gone away completely?	◯ Yes<br>◯ No
Have you experienced this symptom (at any time)?	☐ Yes

When did the symptom begin?	______________________
Does/Did this symptom come and go?	◯ Yes<br>◯ No
Has the symptom gone away completely?	◯ Yes<br>◯ No
Have you experienced this symptom (at any time)?	☐ Yes
When did the symptom begin?	______________________
Does/Did this symptom come and go?	◯ Yes<br>◯ No
Has the symptom gone away completely?	◯ Yes<br>◯ No
Have you experienced this symptom (at any time)?	☐ Yes
When did the symptom begin?	______________________
Does/Did this symptom come and go?	◯ Yes<br>◯ No
Has the symptom gone away completely?	◯ Yes<br>◯ No
Have you experienced this symptom (at any time)?	☐ Yes
When did the symptom begin?	______________________
Does/Did this symptom come and go?	◯ Yes<br>◯ No
Has the symptom gone away completely?	◯ Yes<br>◯ No
Have you experienced this symptom (at any time)?	☐ Yes
When did the symptom begin?	______________________
Does/Did this symptom come and go?	◯ Yes<br>◯ No
Has the symptom gone away completely?	◯ Yes<br>◯ No
Have you experienced this symptom (at any time)?	☐ Yes

When did the symptom begin?	__________________________
Does/Did this symptom come and go?	◯ Yes<br>◯ No
Has the symptom gone away completely?	◯ Yes<br>◯ No
Have you experienced this symptom (at any time)?	☐ Yes
When did the symptom begin?	__________________________
Does/Did this symptom come and go?	◯ Yes<br>◯ No
Has the symptom gone away completely?	◯ Yes<br>◯ No
Have you experienced this symptom (at any time)?	☐ Yes
When did the symptom begin?	__________________________
Does/Did this symptom come and go?	◯ Yes<br>◯ No
Has the symptom gone away completely?	◯ Yes<br>◯ No
Have you experienced this symptom (at any time)?	☐ Yes
When did the symptom begin?	__________________________
Does/Did this symptom come and go?	◯ Yes<br>◯ No
Has the symptom gone away completely?	◯ Yes<br>◯ No
Have you experienced this symptom (at any time)?	☐ Yes
When did the symptom begin?	__________________________
Does/Did this symptom come and go?	◯ Yes<br>◯ No
Has the symptom gone away completely?	◯ Yes<br>◯ No
Have you experienced this symptom (at any time)?	☐ Yes

When did the symptom begin?	__________________
Does/Did this symptom come and go?	◯ Yes<br>◯ No
Has the symptom gone away completely?	◯ Yes<br>◯ No
Have you experienced this symptom (at any time)?	☐ Yes
When did the symptom begin?	__________________
Does/Did this symptom come and go?	◯ Yes<br>◯ No
Has the symptom gone away completely?	◯ Yes<br>◯ No
Have you experienced this symptom (at any time)?	☐ Yes
When did the symptom begin?	__________________
Does/Did this symptom come and go?	◯ Yes<br>◯ No
Has the symptom gone away completely?	◯ Yes<br>◯ No
Have you experienced this symptom (at any time)?	☐ Yes
When did the symptom begin?	__________________
Does/Did this symptom come and go?	◯ Yes<br>◯ No
Has the symptom gone away completely?	◯ Yes<br>◯ No
Have you experienced this symptom (at any time)?	☐ Yes
When did the symptom begin?	__________________
Does/Did this symptom come and go?	◯ Yes<br>◯ No
Has the symptom gone away completely?	◯ Yes<br>◯ No
Have you experienced this symptom (at any time)?	☐ Yes

When did the symptom begin?	__________________________________
Does/Did this symptom come and go?	◯ Yes<br>◯ No
Has the symptom gone away completely?	◯ Yes<br>◯ No
Have you experienced this symptom (at any time)?	☐ Yes
When did the symptom begin?	__________________________________
Does/Did this symptom come and go?	◯ Yes<br>◯ No
Has the symptom gone away completely?	◯ Yes<br>◯ No
Have you experienced this symptom (at any time)?	☐ Yes
When did the symptom begin?	__________________________________
Does/Did this symptom come and go?	◯ Yes<br>◯ No
Has the symptom gone away completely?	◯ Yes<br>◯ No
Have you experienced this symptom (at any time)?	☐ Yes
When did the symptom begin?	__________________________________
Does/Did this symptom come and go?	◯ Yes<br>◯ No
Has the symptom gone away completely?	◯ Yes<br>◯ No
Have you experienced this symptom (at any time)?	☐ Yes
When did the symptom begin?	__________________________________
Does/Did this symptom come and go?	◯ Yes<br>◯ No
Has the symptom gone away completely?	◯ Yes<br>◯ No
Have you experienced this symptom (at any time)?	☐ Yes

When did the symptom begin?	__________________
Does/Did this symptom come and go?	◯ Yes<br>◯ No
Has the symptom gone away completely?	◯ Yes<br>◯ No
Have you experienced this symptom (at any time)?	☐ Yes
When did the symptom begin?	__________________
Does/Did this symptom come and go?	◯ Yes<br>◯ No
Has the symptom gone away completely?	◯ Yes<br>◯ No
Have you experienced this symptom (at any time)?	☐ Yes
When did the symptom begin?	__________________
Does/Did this symptom come and go?	◯ Yes<br>◯ No
Has the symptom gone away completely?	◯ Yes<br>◯ No
Have you experienced this symptom (at any time)?	☐ Yes
When did the symptom begin?	__________________
Does/Did this symptom come and go?	◯ Yes<br>◯ No
Has the symptom gone away completely?	◯ Yes<br>◯ No
Have you experienced this symptom (at any time)?	☐ Yes
When did the symptom begin?	__________________
Does/Did this symptom come and go?	◯ Yes<br>◯ No
Has the symptom gone away completely?	◯ Yes<br>◯ No
Have you experienced this symptom (at any time)?	☐ Yes

When did the symptom begin?	__________________________________
Does/Did this symptom come and go?	◯ Yes<br>◯ No
Has the symptom gone away completely?	◯ Yes<br>◯ No
Have you experienced this symptom (at any time)?	☐ Yes
When did the symptom begin?	__________________________________
Does/Did this symptom come and go?	◯ Yes<br>◯ No
Has the symptom gone away completely?	◯ Yes<br>◯ No
Have you experienced this symptom (at any time)?	☐ Yes
When did the symptom begin?	__________________________________
Does/Did this symptom come and go?	◯ Yes<br>◯ No
Has the symptom gone away completely?	◯ Yes<br>◯ No
Have you experienced this symptom (at any time)?	☐ Yes
When did the symptom begin?	__________________________________
Does/Did this symptom come and go?	◯ Yes<br>◯ No
Has the symptom gone away completely?	◯ Yes<br>◯ No
Have you experienced this symptom (at any time)?	☐ Yes
When did the symptom begin?	__________________________________
Does/Did this symptom come and go?	◯ Yes<br>◯ No
Has the symptom gone away completely?	◯ Yes<br>◯ No
Have you experienced this symptom (at any time)?	☐ Yes

When did the symptom begin?	__________________
Does/Did this symptom come and go?	◯ Yes<br>◯ No
Has the symptom gone away completely?	◯ Yes<br>◯ No
Have you experienced this symptom (at any time)?	☐ Yes
When did the symptom begin?	__________________
Does/Did this symptom come and go?	◯ Yes<br>◯ No
Has the symptom gone away completely?	◯ Yes<br>◯ No
Have you experienced this symptom (at any time)?	☐ Yes
When did the symptom begin?	__________________
Does/Did this symptom come and go?	◯ Yes<br>◯ No
Has the symptom gone away completely?	◯ Yes<br>◯ No
Have you experienced this symptom (at any time)?	☐ Yes
When did the symptom begin?	__________________
Does/Did this symptom come and go?	◯ Yes<br>◯ No
Has the symptom gone away completely?	◯ Yes<br>◯ No
Have you experienced this symptom (at any time)?	☐ Yes
When did the symptom begin?	__________________
Does/Did this symptom come and go?	◯ Yes<br>◯ No
Has the symptom gone away completely?	◯ Yes<br>◯ No
Have you experienced this symptom (at any time)?	☐ Yes

When did the symptom begin?	__________________________________
Does/Did this symptom come and go?	◯ Yes<br>◯ No
Has the symptom gone away completely?	◯ Yes<br>◯ No
Have you experienced this symptom (at any time)?	☐ Yes
When did the symptom begin?	__________________________________
Does/Did this symptom come and go?	◯ Yes<br>◯ No
Has the symptom gone away completely?	◯ Yes<br>◯ No
Have you experienced this symptom (at any time)?	☐ Yes
When did the symptom begin?	__________________________________
Does/Did this symptom come and go?	◯ Yes<br>◯ No
Has the symptom gone away completely?	◯ Yes<br>◯ No
Have you experienced this symptom (at any time)?	☐ Yes
When did the symptom begin?	__________________________________
Does/Did this symptom come and go?	◯ Yes<br>◯ No
Has the symptom gone away completely?	◯ Yes<br>◯ No
Have you experienced this symptom (at any time)?	☐ Yes
When did the symptom begin?	__________________________________
Does/Did this symptom come and go?	◯ Yes<br>◯ No
Has the symptom gone away completely?	◯ Yes<br>◯ No
Have you experienced this symptom (at any time)?	☐ Yes

When did the symptom begin?	__________________________________
Does/Did this symptom come and go?	◯ Yes<br>◯ No
Has the symptom gone away completely?	◯ Yes<br>◯ No
Have you experienced this symptom (at any time)?	☐ Yes
When did the symptom begin?	__________________________________
Does/Did this symptom come and go?	◯ Yes<br>◯ No
Has the symptom gone away completely?	◯ Yes<br>◯ No
Have you experienced this symptom (at any time)?	☐ Yes
When did the symptom begin?	__________________________________
Does/Did this symptom come and go?	◯ Yes<br>◯ No
Has the symptom gone away completely?	◯ Yes<br>◯ No
Have you experienced this symptom (at any time)?	☐ Yes
When did the symptom begin?	__________________________________
Does/Did this symptom come and go?	◯ Yes<br>◯ No
Has the symptom gone away completely?	◯ Yes<br>◯ No
Have you experienced this symptom (at any time)?	☐ Yes
When did the symptom begin?	__________________________________
Does/Did this symptom come and go?	◯ Yes<br>◯ No
Has the symptom gone away completely?	◯ Yes<br>◯ No
Have you experienced this symptom (at any time)?	☐ Yes

When did the symptom begin?	__________________
Does/Did this symptom come and go?	◯ Yes<br>◯ No
Has the symptom gone away completely?	◯ Yes<br>◯ No
Have you experienced this symptom (at any time)?	☐ Yes
When did the symptom begin?	__________________
Does/Did this symptom come and go?	◯ Yes<br>◯ No
Has the symptom gone away completely?	◯ Yes<br>◯ No
Have you experienced this symptom (at any time)?	☐ Yes
When did the symptom begin?	__________________
Does/Did this symptom come and go?	◯ Yes<br>◯ No
Has the symptom gone away completely?	◯ Yes<br>◯ No
Have you experienced this symptom (at any time)?	☐ Yes
When did the symptom begin?	__________________
Does/Did this symptom come and go?	◯ Yes<br>◯ No
Has the symptom gone away completely?	◯ Yes<br>◯ No

While experiencing these symptoms, how much do/did they bother you in terms of discomfort or pain?

	Not at all	A little bit	Somewhat	Quite a bit	Very much
Abdominal pain	◯	◯	◯	◯	◯
Abnormally low temperature	◯	◯	◯	◯	◯
Acid reflux	◯	◯	◯	◯	◯
Afternoon or evening fevers/low-grade fevers	◯	◯	◯	◯	◯
Anemia (low number of red blood cells)	◯	◯	◯	◯	◯
Anxiety	◯	◯	◯	◯	◯
Arrythmia (improper beating of the heart due to electrical impulse problems)	◯	◯	◯	◯	◯
Bilateral neck throbbing around lymph nodes	◯	◯	◯	◯	◯
Blurry vision	◯	◯	◯	◯	◯
Bone aches in extremities	◯	◯	◯	◯	◯
Brain fog	◯	◯	◯	◯	◯
Brain pressure	◯	◯	◯	◯	◯
Bulging veins	◯	◯	◯	◯	◯
Burning sensations	◯	◯	◯	◯	◯
Bruising of skin	◯	◯	◯	◯	◯
Calf cramps	◯	◯	◯	◯	◯
Change in nails (i.e. white spots, brittleness, change in moons)	◯	◯	◯	◯	◯
Changes in voice	◯	◯	◯	◯	◯
Changed sense of taste	◯	◯	◯	◯	◯
Chills but no fever	◯	◯	◯	◯	◯
Clogged ears	◯	◯	◯	◯	◯
Cold burning feeling in lungs	◯	◯	◯	◯	◯
Confusion	◯	◯	◯	◯	◯
Congested or runny nose	◯	◯	◯	◯	◯
Constant thirst	◯	◯	◯	◯	◯
Costochondritis (inflammation of the cartilage that connects a rib to the breastbone)	◯	◯	◯	◯	◯
Cough	◯	◯	◯	◯	◯
Coughing up blood	◯	◯	◯	◯	◯

Covid toes (tender or itchy rash or chilblains on the toes or foot)	◯	◯	◯	◯	◯
Cracked or dry lips	◯	◯	◯	◯	◯
Dental problems (i.e. chipped tooth, tooth loss)	◯	◯	◯	◯	◯
Diarrhea	◯	◯	◯	◯	◯
Difficulty concentrating or focusing	◯	◯	◯	◯	◯
Difficulty sleeping	◯	◯	◯	◯	◯
Difficulty speaking properly	◯	◯	◯	◯	◯
Discoloration of the skin (for example: purple or blue on the hands or feet, no blistering)	◯	◯	◯	◯	◯
Dizziness	◯	◯	◯	◯	◯
Dry eyes	◯	◯	◯	◯	◯
Dry or peeling skin	◯	◯	◯	◯	◯
Dry scalp or dandruff	◯	◯	◯	◯	◯
Dry throat	◯	◯	◯	◯	◯
Ear pain/earache	◯	◯	◯	◯	◯
Elevated thyroid hormones	◯	◯	◯	◯	◯
Extreme pressure at base of head or occipital nerve	◯	◯	◯	◯	◯
Eye stye or infection	◯	◯	◯	◯	◯
Fatigue	◯	◯	◯	◯	◯
Feeling irritable	◯	◯	◯	◯	◯
Feeling of burning skin	◯	◯	◯	◯	◯
Fever or chills	◯	◯	◯	◯	◯
Floaters or flashes of light in vision	◯	◯	◯	◯	◯
Foot pain	◯	◯	◯	◯	◯
GERD (acid reflux) with excessive salivation	◯	◯	◯	◯	◯
Goiter or lump in throat	◯	◯	◯	◯	◯
Hair loss	◯	◯	◯	◯	◯
Hand or wrist pain	◯	◯	◯	◯	◯
Headache	◯	◯	◯	◯	◯
Heart palpitations (heart skipping a beat or racing)	◯	◯	◯	◯	◯
Heat intolerance	◯	◯	◯	◯	◯
High blood pressure	◯	◯	◯	◯	◯
Hormone imbalances	◯	◯	◯	◯	◯

"Hot" blood rush	◯	◯	◯	◯	◯
Inability to cry	◯	◯	◯	◯	◯
Inability to exercise or be active	◯	◯	◯	◯	◯
Inability to yawn	◯	◯	◯	◯	◯
Internal tremors or buzzing/vibration	◯	◯	◯	◯	◯
Irregular or skipped menstrual cycles	◯	◯	◯	◯	◯
Jaw pain	◯	◯	◯	◯	◯
Joint pain	◯	◯	◯	◯	◯
Kidney issues or protein in urine	◯	◯	◯	◯	◯
Kidney pain	◯	◯	◯	◯	◯
Low blood oxygen	◯	◯	◯	◯	◯
Low blood pressure	◯	◯	◯	◯	◯
Lower back pain	◯	◯	◯	◯	◯
Loss of appetite	◯	◯	◯	◯	◯
Loss of hearing	◯	◯	◯	◯	◯
Loss or decrease in quality of vision	◯	◯	◯	◯	◯
Lump in throat/difficulty swallowing	◯	◯	◯	◯	◯
Memory problems	◯	◯	◯	◯	◯
Menstrual cycles that are heavier or lighter than normal	◯	◯	◯	◯	◯
Mid-back pain at base of ribs	◯	◯	◯	◯	◯
Mouth sores or sore tongue	◯	◯	◯	◯	◯
Muscle or body aches	◯	◯	◯	◯	◯
Muscle twitching	◯	◯	◯	◯	◯
Nausea or vomiting	◯	◯	◯	◯	◯
Neck muscle pain	◯	◯	◯	◯	◯
Nerve pain	◯	◯	◯	◯	◯
Nerve sensations (tingling, pins and needles, numbness)	◯	◯	◯	◯	◯
Neuropathy in feet and hands (weakness, numbness, and pain)	◯	◯	◯	◯	◯
New allergies	◯	◯	◯	◯	◯
Night sweats	◯	◯	◯	◯	◯
Nightmares	◯	◯	◯	◯	◯
Painful scalp	◯	◯	◯	◯	◯
Partial or complete loss of sense of smell	◯	◯	◯	◯	◯

Partial or complete loss of sense of taste	○	○	○	○	○
Persistent chest pain or pressure	○	○	○	○	○
Personality change (drastic)	○	○	○	○	○
Petechiae (pinpoint rash)	○	○	○	○	○
Phantom smells	○	○	○	○	○
Phlegm in back of throat	○	○	○	○	○
Post-exertional malaise (worsened symptoms or flu-like symptoms after exertion)	○	○	○	○	○
Postnasal drip	○	○	○	○	○
Rash	○	○	○	○	○
Reflux or heartburn	○	○	○	○	○
Runny nose	○	○	○	○	○
Sadness	○	○	○	○	○
Seizures	○	○	○	○	○
Sharp or sudden chest pain	○	○	○	○	○
Shortness of breath or difficulty breathing	○	○	○	○	○
Shortness of breath or exhaustion from bending over	○	○	○	○	○
Sleeping more than normal	○	○	○	○	○
Sore throat	○	○	○	○	○
Spinal issues	○	○	○	○	○
Spikes in blood pressure	○	○	○	○	○
Swollen hands or feet	○	○	○	○	○
Swollen lymph nodes	○	○	○	○	○
Syncope (fainting)	○	○	○	○	○
Tachycardia (rapid heartbeat) at rest	○	○	○	○	○
Tachycardia (rapid heartbeat) after standing up	○	○	○	○	○
Thrush (white fungal infection in the mouth or throat)	○	○	○	○	○
Tinnitus or humming in ears	○	○	○	○	○
Tremors or shakiness	○	○	○	○	○
Upper back pain	○	○	○	○	○
UTI (urinary tract infection)	○	○	○	○	○
Weakened neck	○	○	○	○	○
Weight gain	○	○	○	○	○

After quarantine, how much does/did the symptom impair your ability to work compared to pre-COVID?					
Abdominal pain	◯	◯	◯	◯	◯
Abnormally low temperature	◯	◯	◯	◯	◯
Acid reflux	◯	◯	◯	◯	◯
Afternoon or evening fevers/low-grade fevers	◯	◯	◯	◯	◯
Anemia (low number of red blood cells)	◯	◯	◯	◯	◯
Anxiety	◯	◯	◯	◯	◯
Arrythmia (improper beating of the heart due to electrical impulse problems)	◯	◯	◯	◯	◯
Bilateral neck throbbing around lymph nodes	◯	◯	◯	◯	◯
Blurry vision	◯	◯	◯	◯	◯
Bone aches in extremities	◯	◯	◯	◯	◯
Brain fog	◯	◯	◯	◯	◯
Brain pressure	◯	◯	◯	◯	◯
Bulging veins	◯	◯	◯	◯	◯
Burning sensations	◯	◯	◯	◯	◯
Bruising of skin	◯	◯	◯	◯	◯
Calf cramps	◯	◯	◯	◯	◯
Change in nails (i.e. white spots, brittleness, change in moons)	◯	◯	◯	◯	◯
Changes in voice	◯	◯	◯	◯	◯
Changed sense of taste	◯	◯	◯	◯	◯
Chills but no fever	◯	◯	◯	◯	◯
Clogged ears	◯	◯	◯	◯	◯
Cold burning feeling in lungs	◯	◯	◯	◯	◯
Confusion	◯	◯	◯	◯	◯
Congested or runny nose	◯	◯	◯	◯	◯
Constant thirst	◯	◯	◯	◯	◯
Costochondritis (inflammation of the cartilage that connects a rib to the breastbone)	◯	◯	◯	◯	◯
Cough	◯	◯	◯	◯	◯
Coughing up blood	◯	◯	◯	◯	◯
Covid toes (tender or itchy rash or chilblains on the toes or foot)	◯	◯	◯	◯	◯

Cracked or dry lips	◯	◯	◯	◯	◯
Dental problems (i.e. chipped tooth, tooth loss)	◯	◯	◯	◯	◯
Diarrhea	◯	◯	◯	◯	◯
Difficulty concentrating or focusing	◯	◯	◯	◯	◯
Difficulty sleeping	◯	◯	◯	◯	◯
Difficulty speaking properly	◯	◯	◯	◯	◯
Discoloration of the skin (for example: purple or blue on the hands or feet, no blistering)	◯	◯	◯	◯	◯
Dizziness	◯	◯	◯	◯	◯
Dry eyes	◯	◯	◯	◯	◯
Dry or peeling skin	◯	◯	◯	◯	◯
Dry scalp or dandruff	◯	◯	◯	◯	◯
Dry throat	◯	◯	◯	◯	◯
Ear pain/earache	◯	◯	◯	◯	◯
Elevated thyroid hormones	◯	◯	◯	◯	◯
Extreme pressure at base of head or occipital nerve	◯	◯	◯	◯	◯
Eye stye or infection	◯	◯	◯	◯	◯
Fatigue	◯	◯	◯	◯	◯
Feeling irritable	◯	◯	◯	◯	◯
Feeling of burning skin	◯	◯	◯	◯	◯
Fever or chills	◯	◯	◯	◯	◯
Floaters or flashes of light in vision	◯	◯	◯	◯	◯
Foot pain	◯	◯	◯	◯	◯
GERD (acid reflux) with excessive salivation	◯	◯	◯	◯	◯
Goiter or lump in throat	◯	◯	◯	◯	◯
Hair loss	◯	◯	◯	◯	◯
Hand or wrist pain	◯	◯	◯	◯	◯
Headache	◯	◯	◯	◯	◯
Heart palpitations (heart skipping a beat or racing)	◯	◯	◯	◯	◯
Heat intolerance	◯	◯	◯	◯	◯
High blood pressure	◯	◯	◯	◯	◯
Hormone imbalances	◯	◯	◯	◯	◯
"Hot" blood rush	◯	◯	◯	◯	◯
Inability to cry	◯	◯	◯	◯	◯

Inability to exercise or be active	○	○	○	○	○
Inability to yawn	○	○	○	○	○
Internal tremors or buzzing/vibration	○	○	○	○	○
Irregular or skipped menstrual cycles	○	○	○	○	○
Jaw pain	○	○	○	○	○
Joint pain	○	○	○	○	○
Kidney issues or protein in urine	○	○	○	○	○
Kidney pain	○	○	○	○	○
Low blood oxygen	○	○	○	○	○
Low blood pressure	○	○	○	○	○
Lower back pain	○	○	○	○	○
Loss of appetite	○	○	○	○	○
Loss of hearing	○	○	○	○	○
Loss or decrease in quality of vision	○	○	○	○	○
Lump in throat/difficulty swallowing	○	○	○	○	○
Memory problems	○	○	○	○	○
Menstrual cycles that are heavier or lighter than normal	○	○	○	○	○
Mid-back pain at base of ribs	○	○	○	○	○
Mouth sores or sore tongue	○	○	○	○	○
Muscle or body aches	○	○	○	○	○
Muscle twitching	○	○	○	○	○
Nausea or vomiting	○	○	○	○	○
Neck muscle pain	○	○	○	○	○
Nerve pain	○	○	○	○	○
Nerve sensations (tingling, pins and needles, numbness)	○	○	○	○	○
Neuropathy in feet and hands (weakness, numbness, and pain)	○	○	○	○	○
New allergies	○	○	○	○	○
Night sweats	○	○	○	○	○
Nightmares	○	○	○	○	○
Painful scalp	○	○	○	○	○
Partial or complete loss of sense of smell	○	○	○	○	○
Partial or complete loss of sense of taste	○	○	○	○	○

Persistent chest pain or pressure	○	○	○	○	○
Personality change (drastic)	○	○	○	○	○
Petechiae (pinpoint rash)	○	○	○	○	○
Phantom smells	○	○	○	○	○
Phlegm in back of throat	○	○	○	○	○
Post-exertional malaise (worsened symptoms or flu-like symptoms after exertion)	○	○	○	○	○
Postnasal drip	○	○	○	○	○
Rash	○	○	○	○	○
Reflux or heartburn	○	○	○	○	○
Runny nose	○	○	○	○	○
Sadness	○	○	○	○	○
Seizures	○	○	○	○	○
Sharp or sudden chest pain	○	○	○	○	○
Shortness of breath or difficulty breathing	○	○	○	○	○
Shortness of breath or exhaustion from bending over	○	○	○	○	○
Sleeping more than normal	○	○	○	○	○
Sore throat	○	○	○	○	○
Spinal issues	○	○	○	○	○
Spikes in blood pressure	○	○	○	○	○
Swollen hands or feet	○	○	○	○	○
Swollen lymph nodes	○	○	○	○	○
Syncope (fainting)	○	○	○	○	○
Tachycardia (rapid heartbeat) at rest	○	○	○	○	○
Tachycardia (rapid heartbeat) after standing up	○	○	○	○	○
Thrush (white fungal infection in the mouth or throat)	○	○	○	○	○
Tinnitus or humming in ears	○	○	○	○	○
Tremors or shakiness	○	○	○	○	○
Upper back pain	○	○	○	○	○
UTI (urinary tract infection)	○	○	○	○	○
Weakened neck	○	○	○	○	○
Weight gain	○	○	○	○	○

After quarantine, how much does/did the symptom impair your social or family functioning compared to pre-COVID?

Abdominal pain	○	○	○	○	○
Abnormally low temperature	○	○	○	○	○
Acid reflux	○	○	○	○	○
Afternoon or evening fevers/low-grade fevers	○	○	○	○	○
Anemia (low number of red blood cells)	○	○	○	○	○
Anxiety	○	○	○	○	○
Arrythmia (improper beating of the heart due to electrical impulse problems)	○	○	○	○	○
Bilateral neck throbbing around lymph nodes	○	○	○	○	○
Blurry vision	○	○	○	○	○
Bone aches in extremities	○	○	○	○	○
Brain fog	○	○	○	○	○
Brain pressure	○	○	○	○	○
Bulging veins	○	○	○	○	○
Burning sensations	○	○	○	○	○
Bruising of skin	○	○	○	○	○
Calf cramps	○	○	○	○	○
Change in nails (i.e. white spots, brittleness, change in moons)	○	○	○	○	○
Changes in voice	○	○	○	○	○
Changed sense of taste	○	○	○	○	○
Chills but no fever	○	○	○	○	○
Clogged ears	○	○	○	○	○
Cold burning feeling in lungs	○	○	○	○	○
Confusion	○	○	○	○	○
Congested or runny nose	○	○	○	○	○
Constant thirst	○	○	○	○	○
Costochondritis (inflammation of the cartilage that connects a rib to the breastbone)	○	○	○	○	○
Cough	○	○	○	○	○
Coughing up blood	○	○	○	○	○
Covid toes (tender or itchy rash or chilblains on the toes or foot)	○	○	○	○	○

Cracked or dry lips	○	○	○	○	○
Dental problems (i.e. chipped tooth, tooth loss)	○	○	○	○	○
Diarrhea	○	○	○	○	○
Difficulty concentrating or focusing	○	○	○	○	○
Difficulty sleeping	○	○	○	○	○
Difficulty speaking properly	○	○	○	○	○
Discoloration of the skin (for example: purple or blue on the hands or feet, no blistering)	○	○	○	○	○
Dizziness	○	○	○	○	○
Dry eyes	○	○	○	○	○
Dry or peeling skin	○	○	○	○	○
Dry scalp or dandruff	○	○	○	○	○
Dry throat	○	○	○	○	○
Ear pain/earache	○	○	○	○	○
Elevated thyroid hormones	○	○	○	○	○
Extreme pressure at base of head or occipital nerve	○	○	○	○	○
Eye stye or infection	○	○	○	○	○
Fatigue	○	○	○	○	○
Feeling irritable	○	○	○	○	○
Feeling of burning skin	○	○	○	○	○
Fever or chills	○	○	○	○	○
Floaters or flashes of light in vision	○	○	○	○	○
Foot pain	○	○	○	○	○
GERD (acid reflux) with excessive salivation	○	○	○	○	○
Goiter or lump in throat	○	○	○	○	○
Hair loss	○	○	○	○	○
Hand or wrist pain	○	○	○	○	○
Headache	○	○	○	○	○
Heart palpitations (heart skipping a beat or racing)	○	○	○	○	○
Heat intolerance	○	○	○	○	○
High blood pressure	○	○	○	○	○
Hormone imbalances	○	○	○	○	○
"Hot" blood rush	○	○	○	○	○
Inability to cry	○	○	○	○	○

Inability to exercise or be active	○	○	○	○	○
Inability to yawn	○	○	○	○	○
Internal tremors or buzzing/vibration	○	○	○	○	○
Irregular or skipped menstrual cycles	○	○	○	○	○
Jaw pain	○	○	○	○	○
Joint pain	○	○	○	○	○
Kidney issues or protein in urine	○	○	○	○	○
Kidney pain	○	○	○	○	○
Low blood oxygen	○	○	○	○	○
Low blood pressure	○	○	○	○	○
Lower back pain	○	○	○	○	○
Loss of appetite	○	○	○	○	○
Loss of hearing	○	○	○	○	○
Loss or decrease in quality of vision	○	○	○	○	○
Lump in throat/difficulty swallowing	○	○	○	○	○
Memory problems	○	○	○	○	○
Menstrual cycles that are heavier or lighter than normal	○	○	○	○	○
Mid-back pain at base of ribs	○	○	○	○	○
Mouth sores or sore tongue	○	○	○	○	○
Muscle or body aches	○	○	○	○	○
Muscle twitching	○	○	○	○	○
Nausea or vomiting	○	○	○	○	○
Neck muscle pain	○	○	○	○	○
Nerve pain	○	○	○	○	○
Nerve sensations (tingling, pins and needles, numbness)	○	○	○	○	○
Neuropathy in feet and hands (weakness, numbness, and pain)	○	○	○	○	○
New allergies	○	○	○	○	○
Night sweats	○	○	○	○	○
Nightmares	○	○	○	○	○
Painful scalp	○	○	○	○	○
Partial or complete loss of sense of smell	○	○	○	○	○
Partial or complete loss of sense of taste	○	○	○	○	○

Persistent chest pain or pressure	○	○	○	○	○
Personality change (drastic)	○	○	○	○	○
Petechiae (pinpoint rash)	○	○	○	○	○
Phantom smells	○	○	○	○	○
Phlegm in back of throat	○	○	○	○	○
Post-exertional malaise (worsened symptoms or flu-like symptoms after exertion)	○	○	○	○	○
Postnasal drip	○	○	○	○	○
Rash	○	○	○	○	○
Reflux or heartburn	○	○	○	○	○
Runny nose	○	○	○	○	○
Sadness	○	○	○	○	○
Seizures	○	○	○	○	○
Sharp or sudden chest pain	○	○	○	○	○
Shortness of breath or difficulty breathing	○	○	○	○	○
Shortness of breath or exhaustion from bending over	○	○	○	○	○
Sleeping more than normal	○	○	○	○	○
Sore throat	○	○	○	○	○
Spinal issues	○	○	○	○	○
Spikes in blood pressure	○	○	○	○	○
Swollen hands or feet	○	○	○	○	○
Swollen lymph nodes	○	○	○	○	○
Syncope (fainting)	○	○	○	○	○
Tachycardia (rapid heartbeat) at rest	○	○	○	○	○
Tachycardia (rapid heartbeat) after standing up	○	○	○	○	○
Thrush (white fungal infection in the mouth or throat)	○	○	○	○	○
Tinnitus or humming in ears	○	○	○	○	○
Tremors or shakiness	○	○	○	○	○
Upper back pain	○	○	○	○	○
UTI (urinary tract infection)	○	○	○	○	○
Weakened neck	○	○	○	○	○
Weight gain	○	○	○	○	○

What treatments (medicine, therapy, vitamins, body positioning, at-home care, etc.) have you found effective for reducing your symptoms, if any? ______________________________

	76-100% of health before COVID-19	51-75% of health before COVID-19	26-50% of health before COVID-19	0-25% of health before COVID-19
On your best days, would you say you that you are at	○	○	○	○
On your worst days, would you say that you are at	○	○	○	○

Please, in the space below, share any information about your experience with COVID that we might have missed. ____

Vaccine Verification [old]

Fill this out once you have confirmed that participant has received vaccine.

928) Study Coordinator First Name ______________________

929) Study Coordinator Last Name ______________________

930) Today's date ______________________

931) Participant First Name ______________________

932) Participant Last Name ______________________

933) On what date did participant receive first dose of vaccine? ______________________

934) Has participant confirmed receiving vaccine ☐ Yes

Study Visit 1 Complete

Please complete the survey below as soon as a participant has completed the first study visit.

Thank you!

935) Study Coordinator First Name __________________________________

936) Study Coordinator Last Name __________________________________

937) Participant First Name __________________________________

938) Participant Last Name __________________________________

939) Has the participant completed the first study visit?
- ◯ Yes
- ◯ No

940) Please enter the date of the completed first study visit. __________________________________

Participant Vaccine Verification

Please complete the survey below once you have received the first dose of a Covid-19 vaccine.

Thank you!

941) Please enter your first name ________________________

942) Please enter your last name ________________________

943) Please enter the date you received the first dose of the Covid-19 vaccine ________________________

944) Please select the vaccine type you received

- ○ Pfizer
- ○ Moderna
- ○ Johnson & Johnson (J&J)
- ○ Other

Survey 2

Thank you for taking Survey 2 for the Yale COVID Recovery Study!

Please remember that you may stop this survey and come back to it at any point. To return to the survey, please return to the link in your browser (or reclick the link in your email).

You may also find it helpful to have the below items ready as you complete the survey. If you do not have these items, please still fill in the information as best you can.

Testing results: dates, type (PCR, antigen) and result of tests. If you do not remember the exact date, the estimated date is enough.Symptom time and severity: your symptom log. You will be asked to pick symptoms you have had since you began having COVID-19 symptoms.

Please verify that you received the first dose of your vaccine on [vaccine_arm_1][v_v_vax_date]	◯ This is correct<br>◯ This is not correct ______
On what date did you receive the first dose of your COVID-19 vaccine?	__________________________________
Which COVID-19 vaccine did you receive?	◯ Pfizer-BioNTech<br>◯ Moderna<br>◯ Johnson & Johnson<br>◯ AstraZeneca or Covishield<br>◯ Other<br>◯ Don't know
Have you received your second dose of the vaccine?	◯ Yes ______<br>◯ No, but it's scheduled<br>◯ No, and I don't have an appointment scheduled<br>◯ No, but the vaccine I received requires only 1 dose
When did you receive your second dose of the COVID-19 vaccine?	__________________________________

Before you received the vaccine, you completed Survey 1 and marked the symptoms that you had experienced. Now, we will ask you a series of questions about these symptoms, and how they have changed (if at all). Please answer these questions based on your experience since you received the COVID-19 vaccine, or for the time period from [vaccine_arm_1][v_v_vax_date] until now.

Since [vaccine_arm_1][v_v_vax_date], this symptom has been	Better	Worse	The same
Abdominal pain	○	○	○
Abnormally low temperature	○	○	○
Acid reflux	○	○	○
Afternoon or evening fevers/low-grade fevers	○	○	○
Anemia (low number of red blood cells)	○	○	○
Anxiety	○	○	○
Arrythmia (improper beating of the heart due to electrical impulse problems)	○	○	○
Bilateral neck throbbing around lymph nodes	○	○	○
Blurry vision	○	○	○
Bone aches in extremities	○	○	○
Brain fog	○	○	○
Brain pressure	○	○	○
Bulging veins	○	○	○
Burning sensations	○	○	○
Bruising of skin	○	○	○
Calf cramps	○	○	○
Change in nails (i.e. white spots, brittleness, change in moons)	○	○	○
Changes in voice	○	○	○
Changed sense of taste	○	○	○
Chills but no fever	○	○	○
Clogged ears	○	○	○
Cold burning feeling in lungs	○	○	○
Confusion	○	○	○
Congested or runny nose	○	○	○
Constant thirst	○	○	○
Costochondritis (inflammation of the cartilage that connects a rib to the breastbone)	○	○	○
Cough	○	○	○
Coughing up blood	○	○	○
Covid toes (tender or itchy rash or chilblains on the toes or foot)	○	○	○

Cracked or dry lips	◯	◯	◯
Dental problems (i.e. chipped tooth, tooth loss)	◯	◯	◯
Diarrhea	◯	◯	◯
Difficulty concentrating or focusing	◯	◯	◯
Difficulty sleeping	◯	◯	◯
Difficulty speaking properly	◯	◯	◯
Discoloration of the skin (for example: purple or blue on the hands or feet, no blistering)	◯	◯	◯
Dizziness	◯	◯	◯
Dry eyes	◯	◯	◯
Dry or peeling skin	◯	◯	◯
Dry scalp or dandruff	◯	◯	◯
Dry throat	◯	◯	◯
Ear pain/earache	◯	◯	◯
Elevated thyroid hormones	◯	◯	◯
Extreme pressure at base of head or occipital nerve	◯	◯	◯
Eye stye or infection	◯	◯	◯
Fatigue	◯	◯	◯
Feeling irritable	◯	◯	◯
Feeling of burning skin	◯	◯	◯
Fever or chills	◯	◯	◯
Floaters or flashes of light in vision	◯	◯	◯
Foot pain	◯	◯	◯
GERD (acid reflux) with excessive salivation	◯	◯	◯
Goiter or lump in throat	◯	◯	◯
Hair loss	◯	◯	◯
Hand or wrist pain	◯	◯	◯
Headache	◯	◯	◯
Heart palpitations (heart skipping a beat or racing)	◯	◯	◯
Heat intolerance	◯	◯	◯
High blood pressure	◯	◯	◯
Hormone imbalances	◯	◯	◯
"Hot" blood rush	◯	◯	◯
Inability to cry	◯	◯	◯

Inability to exercise or be active	◯	◯	◯
Inability to yawn	◯	◯	◯
Internal tremors or buzzing/vibration	◯	◯	◯
Irregular or skipped menstrual cycles	◯	◯	◯
Jaw pain	◯	◯	◯
Joint pain	◯	◯	◯
Kidney issues or protein in urine	◯	◯	◯
Kidney pain	◯	◯	◯
Low blood oxygen	◯	◯	◯
Low blood pressure	◯	◯	◯
Lower back pain	◯	◯	◯
Loss of appetite	◯	◯	◯
Loss of hearing	◯	◯	◯
Loss or decrease in quality of vision	◯	◯	◯
Lump in throat/difficulty swallowing	◯	◯	◯
Memory problems	◯	◯	◯
Menstrual cycles that are heavier or lighter than normal	◯	◯	◯
Mid-back pain at base of ribs	◯	◯	◯
Mouth sores or sore tongue	◯	◯	◯
Muscle or body aches	◯	◯	◯
Muscle twitching	◯	◯	◯
Nausea or vomiting	◯	◯	◯
Neck muscle pain	◯	◯	◯
Nerve pain	◯	◯	◯
Nerve sensations (tingling, pins and needles, numbness)	◯	◯	◯
Neuropathy in feet and hands (weakness, numbness, and pain)	◯	◯	◯
New allergies	◯	◯	◯
Night sweats	◯	◯	◯
Nightmares	◯	◯	◯
Painful scalp	◯	◯	◯
Partial or complete loss of sense of smell	◯	◯	◯
Partial or complete loss of sense of taste	◯	◯	◯

Persistent chest pain or pressure	○	○	○
Personality change (drastic)	○	○	○
Petechiae (pinpoint rash)	○	○	○
Phantom smells	○	○	○
Phlegm in back of throat	○	○	○
Post-exertional malaise (worsened symptoms or flu-like symptoms after exertion)	○	○	○
Postnasal drip	○	○	○
Rash	○	○	○
Reflux or heartburn	○	○	○
Runny nose	○	○	○
Sadness	○	○	○
Seizures	○	○	○
Sharp or sudden chest pain	○	○	○
Shortness of breath or difficulty breathing	○	○	○
Shortness of breath or exhaustion from bending over	○	○	○
Sleeping more than normal	○	○	○
Sore throat	○	○	○
Spinal issues	○	○	○
Spikes in blood pressure	○	○	○
Swollen hands or feet	○	○	○
Swollen lymph nodes	○	○	○
Syncope (fainting)	○	○	○
Tachycardia (rapid heartbeat) at rest	○	○	○
Tachycardia (rapid heartbeat) after standing up	○	○	○
Thrush (white fungal infection in the mouth or throat)	○	○	○
Tinnitus or humming in ears	○	○	○
Tremors or shakiness	○	○	○
Upper back pain	○	○	○
UTI (urinary tract infection)	○	○	○
Weakened neck	○	○	○
Weight gain	○	○	○

Since [vaccine_arm_1][v_v_vax_date], I have experienced this symptom, on average

	Never--it has gone away completely	Once a month	Once or twice a week	3 or 4 times a week	Most days	All the time
Abdominal pain	○	○	○	○	○	○
Abnormally low temperature	○	○	○	○	○	○
Acid reflux	○	○	○	○	○	○
Afternoon or evening fevers/low-grade fevers	○	○	○	○	○	○
Anemia (low number of red blood cells)	○	○	○	○	○	○
Anxiety	○	○	○	○	○	○
Arrythmia (improper beating of the heart due to electrical impulse problems)	○	○	○	○	○	○
Bilateral neck throbbing around lymph nodes	○	○	○	○	○	○
Blurry vision	○	○	○	○	○	○
Bone aches in extremities	○	○	○	○	○	○
Brain fog	○	○	○	○	○	○
Brain pressure	○	○	○	○	○	○
Bulging veins	○	○	○	○	○	○
Burning sensations	○	○	○	○	○	○
Bruising of skin	○	○	○	○	○	○
Calf cramps	○	○	○	○	○	○
Change in nails (i.e. white spots, brittleness, change in moons)	○	○	○	○	○	○
Changes in voice	○	○	○	○	○	○
Changed sense of taste	○	○	○	○	○	○
Chills but no fever	○	○	○	○	○	○
Clogged ears	○	○	○	○	○	○
Cold burning feeling in lungs	○	○	○	○	○	○
Confusion	○	○	○	○	○	○
Congested or runny nose	○	○	○	○	○	○
Constant thirst	○	○	○	○	○	○
Costochondritis (inflammation of the cartilage that connects a rib to the breastbone)	○	○	○	○	○	○
Cough	○	○	○	○	○	○
Coughing up blood	○	○	○	○	○	○

Covid toes (tender or itchy rash or chilblains on the toes or foot)	○	○	○	○	○	○
Cracked or dry lips	○	○	○	○	○	○
Dental problems (i.e. chipped tooth, tooth loss)	○	○	○	○	○	○
Diarrhea	○	○	○	○	○	○
Difficulty concentrating or focusing	○	○	○	○	○	○
Difficulty sleeping	○	○	○	○	○	○
Difficulty speaking properly	○	○	○	○	○	○
Discoloration of the skin (for example: purple or blue on the hands or feet, no blistering)	○	○	○	○	○	○
Dizziness	○	○	○	○	○	○
Dry eyes	○	○	○	○	○	○
Dry or peeling skin	○	○	○	○	○	○
Dry scalp or dandruff	○	○	○	○	○	○
Dry throat	○	○	○	○	○	○
Ear pain/earache	○	○	○	○	○	○
Elevated thyroid hormones	○	○	○	○	○	○
Extreme pressure at base of head or occipital nerve	○	○	○	○	○	○
Eye stye or infection	○	○	○	○	○	○
Fatigue	○	○	○	○	○	○
Feeling irritable	○	○	○	○	○	○
Feeling of burning skin	○	○	○	○	○	○
Fever or chills	○	○	○	○	○	○
Floaters or flashes of light in vision	○	○	○	○	○	○
Foot pain	○	○	○	○	○	○
GERD (acid reflux) with excessive salivation	○	○	○	○	○	○
Goiter or lump in throat	○	○	○	○	○	○
Hair loss	○	○	○	○	○	○
Hand or wrist pain	○	○	○	○	○	○
Headache	○	○	○	○	○	○
Heart palpitations (heart skipping a beat or racing)	○	○	○	○	○	○
Heat intolerance	○	○	○	○	○	○
High blood pressure	○	○	○	○	○	○
Hormone imbalances	○	○	○	○	○	○

"Hot" blood rush	○	○	○	○	○	○
Inability to cry	○	○	○	○	○	○
Inability to exercise or be active	○	○	○	○	○	○
Inability to yawn	○	○	○	○	○	○
Internal tremors or buzzing/vibration	○	○	○	○	○	○
Irregular or skipped menstrual cycles	○	○	○	○	○	○
Jaw pain	○	○	○	○	○	○
Joint pain	○	○	○	○	○	○
Kidney issues or protein in urine	○	○	○	○	○	○
Kidney pain	○	○	○	○	○	○
Low blood oxygen	○	○	○	○	○	○
Low blood pressure	○	○	○	○	○	○
Lower back pain	○	○	○	○	○	○
Loss of appetite	○	○	○	○	○	○
Loss of hearing	○	○	○	○	○	○
Loss or decrease in quality of vision	○	○	○	○	○	○
Lump in throat/difficulty swallowing	○	○	○	○	○	○
Memory problems	○	○	○	○	○	○
Menstrual cycles that are heavier or lighter than normal	○	○	○	○	○	○
Mid-back pain at base of ribs	○	○	○	○	○	○
Mouth sores or sore tongue	○	○	○	○	○	○
Muscle or body aches	○	○	○	○	○	○
Muscle twitching	○	○	○	○	○	○
Nausea or vomiting	○	○	○	○	○	○
Neck muscle pain	○	○	○	○	○	○
Nerve pain	○	○	○	○	○	○
Nerve sensations (tingling, pins and needles, numbness)	○	○	○	○	○	○
Neuropathy in feet and hands (weakness, numbness, and pain)	○	○	○	○	○	○
New allergies	○	○	○	○	○	○
Night sweats	○	○	○	○	○	○
Nightmares	○	○	○	○	○	○
Painful scalp	○	○	○	○	○	○
Partial or complete loss of sense of smell	○	○	○	○	○	○

Partial or complete loss of sense of taste	○	○	○	○	○	○
Persistent chest pain or pressure	○	○	○	○	○	○
Personality change (drastic)	○	○	○	○	○	○
Petechiae (pinpoint rash)	○	○	○	○	○	○
Phantom smells	○	○	○	○	○	○
Phlegm in back of throat	○	○	○	○	○	○
Post-exertional malaise (worsened symptoms or flu-like symptoms after exertion)	○	○	○	○	○	○
Postnasal drip	○	○	○	○	○	○
Rash	○	○	○	○	○	○
Reflux or heartburn	○	○	○	○	○	○
Runny nose	○	○	○	○	○	○
Sadness	○	○	○	○	○	○
Seizures	○	○	○	○	○	○
Sharp or sudden chest pain	○	○	○	○	○	○
Shortness of breath or difficulty breathing	○	○	○	○	○	○
Shortness of breath or exhaustion from bending over	○	○	○	○	○	○
Sleeping more than normal	○	○	○	○	○	○
Sore throat	○	○	○	○	○	○
Spinal issues	○	○	○	○	○	○
Spikes in blood pressure	○	○	○	○	○	○
Swollen hands or feet	○	○	○	○	○	○
Swollen lymph nodes	○	○	○	○	○	○
Syncope (fainting)	○	○	○	○	○	○
Tachycardia (rapid heartbeat) at rest	○	○	○	○	○	○
Tachycardia (rapid heartbeat) after standing up	○	○	○	○	○	○
Thrush (white fungal infection in the mouth or throat)	○	○	○	○	○	○
Tinnitus or humming in ears	○	○	○	○	○	○
Tremors or shakiness	○	○	○	○	○	○
Upper back pain	○	○	○	○	○	○
UTI (urinary tract infection)	○	○	○	○	○	○
Weakened neck	○	○	○	○	○	○
Weight gain	○	○	○	○	○	○

Since [vaccine_arm_1][v_v_vax_date], while experiencing the symptom, how much did it bother you in terms of discomfort or pain?

***If you have not experienced the symptom since your vaccine, please choose "Not at all"**

	Not at all	A little bit	Somewhat	Quite a bit	Very much
Abdominal pain	○	○	○	○	○
Abnormally low temperature	○	○	○	○	○
Acid reflux	○	○	○	○	○
Afternoon or evening fevers/low-grade fevers	○	○	○	○	○
Anemia (low number of red blood cells)	○	○	○	○	○
Anxiety	○	○	○	○	○
Arrythmia (improper beating of the heart due to electrical impulse problems)	○	○	○	○	○
Bilateral neck throbbing around lymph nodes	○	○	○	○	○
Blurry vision	○	○	○	○	○
Bone aches in extremities	○	○	○	○	○
Brain fog	○	○	○	○	○
Brain pressure	○	○	○	○	○
Bulging veins	○	○	○	○	○
Burning sensations	○	○	○	○	○
Bruising of skin	○	○	○	○	○
Calf cramps	○	○	○	○	○
Change in nails (i.e. white spots, brittleness, change in moons)	○	○	○	○	○
Changes in voice	○	○	○	○	○
Changed sense of taste	○	○	○	○	○
Chills but no fever	○	○	○	○	○
Clogged ears	○	○	○	○	○
Cold burning feeling in lungs	○	○	○	○	○
Confusion	○	○	○	○	○
Congested or runny nose	○	○	○	○	○
Constant thirst	○	○	○	○	○
Costochondritis (inflammation of the cartilage that connects a rib to the breastbone)	○	○	○	○	○
Cough	○	○	○	○	○

Coughing up blood	◯	◯	◯	◯	◯
Covid toes (tender or itchy rash or chilblains on the toes or foot)	◯	◯	◯	◯	◯
Cracked or dry lips	◯	◯	◯	◯	◯
Dental problems (i.e. chipped tooth, tooth loss)	◯	◯	◯	◯	◯
Diarrhea	◯	◯	◯	◯	◯
Difficulty concentrating or focusing	◯	◯	◯	◯	◯
Difficulty sleeping	◯	◯	◯	◯	◯
Difficulty speaking properly	◯	◯	◯	◯	◯
Discoloration of the skin (for example: purple or blue on the hands or feet, no blistering)	◯	◯	◯	◯	◯
Dizziness	◯	◯	◯	◯	◯
Dry eyes	◯	◯	◯	◯	◯
Dry or peeling skin	◯	◯	◯	◯	◯
Dry scalp or dandruff	◯	◯	◯	◯	◯
Dry throat	◯	◯	◯	◯	◯
Ear pain/earache	◯	◯	◯	◯	◯
Elevated thyroid hormones	◯	◯	◯	◯	◯
Extreme pressure at base of head or occipital nerve	◯	◯	◯	◯	◯
Eye stye or infection	◯	◯	◯	◯	◯
Fatigue	◯	◯	◯	◯	◯
Feeling irritable	◯	◯	◯	◯	◯
Feeling of burning skin	◯	◯	◯	◯	◯
Fever or chills	◯	◯	◯	◯	◯
Floaters or flashes of light in vision	◯	◯	◯	◯	◯
Foot pain	◯	◯	◯	◯	◯
GERD (acid reflux) with excessive salivation	◯	◯	◯	◯	◯
Goiter or lump in throat	◯	◯	◯	◯	◯
Hair loss	◯	◯	◯	◯	◯
Hand or wrist pain	◯	◯	◯	◯	◯
Headache	◯	◯	◯	◯	◯
Heart palpitations (heart skipping a beat or racing)	◯	◯	◯	◯	◯
Heat intolerance	◯	◯	◯	◯	◯
High blood pressure	◯	◯	◯	◯	◯

Hormone imbalances	○	○	○	○	○
"Hot" blood rush	○	○	○	○	○
Inability to cry	○	○	○	○	○
Inability to exercise or be active	○	○	○	○	○
Inability to yawn	○	○	○	○	○
Internal tremors or buzzing/vibration	○	○	○	○	○
Irregular or skipped menstrual cycles	○	○	○	○	○
Jaw pain	○	○	○	○	○
Joint pain	○	○	○	○	○
Kidney issues or protein in urine	○	○	○	○	○
Kidney pain	○	○	○	○	○
Low blood oxygen	○	○	○	○	○
Low blood pressure	○	○	○	○	○
Lower back pain	○	○	○	○	○
Loss of appetite	○	○	○	○	○
Loss of hearing	○	○	○	○	○
Loss or decrease in quality of vision	○	○	○	○	○
Lump in throat/difficulty swallowing	○	○	○	○	○
Memory problems	○	○	○	○	○
Menstrual cycles that are heavier or lighter than normal	○	○	○	○	○
Mid-back pain at base of ribs	○	○	○	○	○
Mouth sores or sore tongue	○	○	○	○	○
Muscle or body aches	○	○	○	○	○
Muscle twitching	○	○	○	○	○
Nausea or vomiting	○	○	○	○	○
Neck muscle pain	○	○	○	○	○
Nerve pain	○	○	○	○	○
Nerve sensations (tingling, pins and needles, numbness)	○	○	○	○	○
Neuropathy in feet and hands (weakness, numbness, and pain)	○	○	○	○	○
New allergies	○	○	○	○	○
Night sweats	○	○	○	○	○
Nightmares	○	○	○	○	○
Painful scalp	○	○	○	○	○

Partial or complete loss of sense of smell	○	○	○	○	○
Partial or complete loss of sense of taste	○	○	○	○	○
Persistent chest pain or pressure	○	○	○	○	○
Personality change (drastic)	○	○	○	○	○
Petechiae (pinpoint rash)	○	○	○	○	○
Phantom smells	○	○	○	○	○
Phlegm in back of throat	○	○	○	○	○
Post-exertional malaise (worsened symptoms or flu-like symptoms after exertion)	○	○	○	○	○
Postnasal drip	○	○	○	○	○
Rash	○	○	○	○	○
Reflux or heartburn	○	○	○	○	○
Runny nose	○	○	○	○	○
Sadness	○	○	○	○	○
Seizures	○	○	○	○	○
Sharp or sudden chest pain	○	○	○	○	○
Shortness of breath or difficulty breathing	○	○	○	○	○
Shortness of breath or exhaustion from bending over	○	○	○	○	○
Sleeping more than normal	○	○	○	○	○
Sore throat	○	○	○	○	○
Spinal issues	○	○	○	○	○
Spikes in blood pressure	○	○	○	○	○
Swollen hands or feet	○	○	○	○	○
Swollen lymph nodes	○	○	○	○	○
Syncope (fainting)	○	○	○	○	○
Tachycardia (rapid heartbeat) at rest	○	○	○	○	○
Tachycardia (rapid heartbeat) after standing up	○	○	○	○	○
Thrush (white fungal infection in the mouth or throat)	○	○	○	○	○
Tinnitus or humming in ears	○	○	○	○	○
Tremors or shakiness	○	○	○	○	○
Upper back pain	○	○	○	○	○
UTI (urinary tract infection)	○	○	○	○	○
Weakened neck	○	○	○	○	○

Weight gain	◯	◯	◯	◯	◯

Since [vaccine_arm_1][v_v_vax_date], while experiencing the symptom, how much did it impair your ability to work compared to pre-COVID?

***If you have not experienced the symptom since your vaccine, please choose "Not at all."**

	Not at all	A little bit	Somewhat	Quite a bit	Very much
Abdominal pain	○	○	○	○	○
Abnormally low temperature	○	○	○	○	○
Acid reflux	○	○	○	○	○
Afternoon or evening fevers/low-grade fevers	○	○	○	○	○
Anemia (low number of red blood cells)	○	○	○	○	○
Anxiety	○	○	○	○	○
Arrythmia (improper beating of the heart due to electrical impulse problems)	○	○	○	○	○
Bilateral neck throbbing around lymph nodes	○	○	○	○	○
Blurry vision	○	○	○	○	○
Bone aches in extremities	○	○	○	○	○
Brain fog	○	○	○	○	○
Brain pressure	○	○	○	○	○
Bulging veins	○	○	○	○	○
Burning sensations	○	○	○	○	○
Bruising of skin	○	○	○	○	○
Calf cramps	○	○	○	○	○
Change in nails (i.e. white spots, brittleness, change in moons)	○	○	○	○	○
Changes in voice	○	○	○	○	○
Changed sense of taste	○	○	○	○	○
Chills but no fever	○	○	○	○	○
Clogged ears	○	○	○	○	○
Cold burning feeling in lungs	○	○	○	○	○
Confusion	○	○	○	○	○
Congested or runny nose	○	○	○	○	○
Constant thirst	○	○	○	○	○
Costochondritis (inflammation of the cartilage that connects a rib to the breastbone)	○	○	○	○	○
Cough	○	○	○	○	○

Coughing up blood	○	○	○	○	○
Covid toes (tender or itchy rash or chilblains on the toes or foot)	○	○	○	○	○
Cracked or dry lips	○	○	○	○	○
Dental problems (i.e. chipped tooth, tooth loss)	○	○	○	○	○
Diarrhea	○	○	○	○	○
Difficulty concentrating or focusing	○	○	○	○	○
Difficulty sleeping	○	○	○	○	○
Difficulty speaking properly	○	○	○	○	○
Discoloration of the skin (for example: purple or blue on the hands or feet, no blistering)	○	○	○	○	○
Dizziness	○	○	○	○	○
Dry eyes	○	○	○	○	○
Dry or peeling skin	○	○	○	○	○
Dry scalp or dandruff	○	○	○	○	○
Dry throat	○	○	○	○	○
Ear pain/earache	○	○	○	○	○
Elevated thyroid hormones	○	○	○	○	○
Extreme pressure at base of head or occipital nerve	○	○	○	○	○
Eye stye or infection	○	○	○	○	○
Fatigue	○	○	○	○	○
Feeling irritable	○	○	○	○	○
Feeling of burning skin	○	○	○	○	○
Fever or chills	○	○	○	○	○
Floaters or flashes of light in vision	○	○	○	○	○
Foot pain	○	○	○	○	○
GERD (acid reflux) with excessive salivation	○	○	○	○	○
Goiter or lump in throat	○	○	○	○	○
Hair loss	○	○	○	○	○
Hand or wrist pain	○	○	○	○	○
Headache	○	○	○	○	○
Heart palpitations (heart skipping a beat or racing)	○	○	○	○	○
Heat intolerance	○	○	○	○	○
High blood pressure	○	○	○	○	○

Hormone imbalances	◯	◯	◯	◯	◯
"Hot" blood rush	◯	◯	◯	◯	◯
Inability to cry	◯	◯	◯	◯	◯
Inability to exercise or be active	◯	◯	◯	◯	◯
Inability to yawn	◯	◯	◯	◯	◯
Internal tremors or buzzing/vibration	◯	◯	◯	◯	◯
Irregular or skipped menstrual cycles	◯	◯	◯	◯	◯
Jaw pain	◯	◯	◯	◯	◯
Joint pain	◯	◯	◯	◯	◯
Kidney issues or protein in urine	◯	◯	◯	◯	◯
Kidney pain	◯	◯	◯	◯	◯
Low blood oxygen	◯	◯	◯	◯	◯
Low blood pressure	◯	◯	◯	◯	◯
Lower back pain	◯	◯	◯	◯	◯
Loss of appetite	◯	◯	◯	◯	◯
Loss of hearing	◯	◯	◯	◯	◯
Loss or decrease in quality of vision	◯	◯	◯	◯	◯
Lump in throat/difficulty swallowing	◯	◯	◯	◯	◯
Memory problems	◯	◯	◯	◯	◯
Menstrual cycles that are heavier or lighter than normal	◯	◯	◯	◯	◯
Mid-back pain at base of ribs	◯	◯	◯	◯	◯
Mouth sores or sore tongue	◯	◯	◯	◯	◯
Muscle or body aches	◯	◯	◯	◯	◯
Muscle twitching	◯	◯	◯	◯	◯
Nausea or vomiting	◯	◯	◯	◯	◯
Neck muscle pain	◯	◯	◯	◯	◯
Nerve pain	◯	◯	◯	◯	◯
Nerve sensations (tingling, pins and needles, numbness)	◯	◯	◯	◯	◯
Neuropathy in feet and hands (weakness, numbness, and pain)	◯	◯	◯	◯	◯
New allergies	◯	◯	◯	◯	◯
Night sweats	◯	◯	◯	◯	◯
Nightmares	◯	◯	◯	◯	◯
Painful scalp	◯	◯	◯	◯	◯

Partial or complete loss of sense of smell	◯	◯	◯	◯	◯
Partial or complete loss of sense of taste	◯	◯	◯	◯	◯
Persistent chest pain or pressure	◯	◯	◯	◯	◯
Personality change (drastic)	◯	◯	◯	◯	◯
Petechiae (pinpoint rash)	◯	◯	◯	◯	◯
Phantom smells	◯	◯	◯	◯	◯
Phlegm in back of throat	◯	◯	◯	◯	◯
Post-exertional malaise (worsened symptoms or flu-like symptoms after exertion)	◯	◯	◯	◯	◯
Postnasal drip	◯	◯	◯	◯	◯
Rash	◯	◯	◯	◯	◯
Reflux or heartburn	◯	◯	◯	◯	◯
Runny nose	◯	◯	◯	◯	◯
Sadness	◯	◯	◯	◯	◯
Seizures	◯	◯	◯	◯	◯
Sharp or sudden chest pain	◯	◯	◯	◯	◯
Shortness of breath or difficulty breathing	◯	◯	◯	◯	◯
Shortness of breath or exhaustion from bending over	◯	◯	◯	◯	◯
Sleeping more than normal	◯	◯	◯	◯	◯
Sore throat	◯	◯	◯	◯	◯
Spinal issues	◯	◯	◯	◯	◯
Spikes in blood pressure	◯	◯	◯	◯	◯
Swollen hands or feet	◯	◯	◯	◯	◯
Swollen lymph nodes	◯	◯	◯	◯	◯
Syncope (fainting)	◯	◯	◯	◯	◯
Tachycardia (rapid heartbeat) at rest	◯	◯	◯	◯	◯
Tachycardia (rapid heartbeat) after standing up	◯	◯	◯	◯	◯
Thrush (white fungal infection in the mouth or throat)	◯	◯	◯	◯	◯
Tinnitus or humming in ears	◯	◯	◯	◯	◯
Tremors or shakiness	◯	◯	◯	◯	◯
Upper back pain	◯	◯	◯	◯	◯
UTI (urinary tract infection)	◯	◯	◯	◯	◯
Weakened neck	◯	◯	◯	◯	◯

Weight gain	○	○	○	○	○

Since [vaccine_arm_1][v_v_vax_date], while experiencing the symptom, how much did it impair your social or family functioning compared to pre-COVID?

***If you have not experienced these symptoms since your vaccine, please choose "Not at all"**

	Not at all	A little bit	Somewhat	Quite a bit	Very much
Abdominal pain	◯	◯	◯	◯	◯
Abnormally low temperature	◯	◯	◯	◯	◯
Acid reflux	◯	◯	◯	◯	◯
Afternoon or evening fevers/low-grade fevers	◯	◯	◯	◯	◯
Anemia (low number of red blood cells)	◯	◯	◯	◯	◯
Anxiety	◯	◯	◯	◯	◯
Arrythmia (improper beating of the heart due to electrical impulse problems)	◯	◯	◯	◯	◯
Bilateral neck throbbing around lymph nodes	◯	◯	◯	◯	◯
Blurry vision	◯	◯	◯	◯	◯
Bone aches in extremities	◯	◯	◯	◯	◯
Brain fog	◯	◯	◯	◯	◯
Brain pressure	◯	◯	◯	◯	◯
Bulging veins	◯	◯	◯	◯	◯
Burning sensations	◯	◯	◯	◯	◯
Bruising of skin	◯	◯	◯	◯	◯
Calf cramps	◯	◯	◯	◯	◯
Change in nails (i.e. white spots, brittleness, change in moons)	◯	◯	◯	◯	◯
Changes in voice	◯	◯	◯	◯	◯
Changed sense of taste	◯	◯	◯	◯	◯
Chills but no fever	◯	◯	◯	◯	◯
Clogged ears	◯	◯	◯	◯	◯
Cold burning feeling in lungs	◯	◯	◯	◯	◯
Confusion	◯	◯	◯	◯	◯
Congested or runny nose	◯	◯	◯	◯	◯
Constant thirst	◯	◯	◯	◯	◯
Costochondritis (inflammation of the cartilage that connects a rib to the breastbone)	◯	◯	◯	◯	◯
Cough	◯	◯	◯	◯	◯

Coughing up blood	○	○	○	○	○
Covid toes (tender or itchy rash or chilblains on the toes or foot)	○	○	○	○	○
Cracked or dry lips	○	○	○	○	○
Dental problems (i.e. chipped tooth, tooth loss)	○	○	○	○	○
Diarrhea	○	○	○	○	○
Difficulty concentrating or focusing	○	○	○	○	○
Difficulty sleeping	○	○	○	○	○
Difficulty speaking properly	○	○	○	○	○
Discoloration of the skin (for example: purple or blue on the hands or feet, no blistering)	○	○	○	○	○
Dizziness	○	○	○	○	○
Dry eyes	○	○	○	○	○
Dry or peeling skin	○	○	○	○	○
Dry scalp or dandruff	○	○	○	○	○
Dry throat	○	○	○	○	○
Ear pain/earache	○	○	○	○	○
Elevated thyroid hormones	○	○	○	○	○
Extreme pressure at base of head or occipital nerve	○	○	○	○	○
Eye stye or infection	○	○	○	○	○
Fatigue	○	○	○	○	○
Feeling irritable	○	○	○	○	○
Feeling of burning skin	○	○	○	○	○
Fever or chills	○	○	○	○	○
Floaters or flashes of light in vision	○	○	○	○	○
Foot pain	○	○	○	○	○
GERD (acid reflux) with excessive salivation	○	○	○	○	○
Goiter or lump in throat	○	○	○	○	○
Hair loss	○	○	○	○	○
Hand or wrist pain	○	○	○	○	○
Headache	○	○	○	○	○
Heart palpitations (heart skipping a beat or racing)	○	○	○	○	○
Heat intolerance	○	○	○	○	○
High blood pressure	○	○	○	○	○

Hormone imbalances	◯	◯	◯	◯	◯
"Hot" blood rush	◯	◯	◯	◯	◯
Inability to cry	◯	◯	◯	◯	◯
Inability to exercise or be active	◯	◯	◯	◯	◯
Inability to yawn	◯	◯	◯	◯	◯
Internal tremors or buzzing/vibration	◯	◯	◯	◯	◯
Irregular or skipped menstrual cycles	◯	◯	◯	◯	◯
Jaw pain	◯	◯	◯	◯	◯
Joint pain	◯	◯	◯	◯	◯
Kidney issues or protein in urine	◯	◯	◯	◯	◯
Kidney pain	◯	◯	◯	◯	◯
Low blood oxygen	◯	◯	◯	◯	◯
Low blood pressure	◯	◯	◯	◯	◯
Lower back pain	◯	◯	◯	◯	◯
Loss of appetite	◯	◯	◯	◯	◯
Loss of hearing	◯	◯	◯	◯	◯
Loss or decrease in quality of vision	◯	◯	◯	◯	◯
Lump in throat/difficulty swallowing	◯	◯	◯	◯	◯
Memory problems	◯	◯	◯	◯	◯
Menstrual cycles that are heavier or lighter than normal	◯	◯	◯	◯	◯
Mid-back pain at base of ribs	◯	◯	◯	◯	◯
Mouth sores or sore tongue	◯	◯	◯	◯	◯
Muscle or body aches	◯	◯	◯	◯	◯
Muscle twitching	◯	◯	◯	◯	◯
Nausea or vomiting	◯	◯	◯	◯	◯
Neck muscle pain	◯	◯	◯	◯	◯
Nerve pain	◯	◯	◯	◯	◯
Nerve sensations (tingling, pins and needles, numbness)	◯	◯	◯	◯	◯
Neuropathy in feet and hands (weakness, numbness, and pain)	◯	◯	◯	◯	◯
New allergies	◯	◯	◯	◯	◯
Night sweats	◯	◯	◯	◯	◯
Nightmares	◯	◯	◯	◯	◯
Painful scalp	◯	◯	◯	◯	◯

Partial or complete loss of sense of smell	○	○	○	○	○
Partial or complete loss of sense of taste	○	○	○	○	○
Persistent chest pain or pressure	○	○	○	○	○
Personality change (drastic)	○	○	○	○	○
Petechiae (pinpoint rash)	○	○	○	○	○
Phantom smells	○	○	○	○	○
Phlegm in back of throat	○	○	○	○	○
Post-exertional malaise (worsened symptoms or flu-like symptoms after exertion)	○	○	○	○	○
Postnasal drip	○	○	○	○	○
Rash	○	○	○	○	○
Reflux or heartburn	○	○	○	○	○
Runny nose	○	○	○	○	○
Sadness	○	○	○	○	○
Seizures	○	○	○	○	○
Sharp or sudden chest pain	○	○	○	○	○
Shortness of breath or difficulty breathing	○	○	○	○	○
Shortness of breath or exhaustion from bending over	○	○	○	○	○
Sleeping more than normal	○	○	○	○	○
Sore throat	○	○	○	○	○
Spinal issues	○	○	○	○	○
Spikes in blood pressure	○	○	○	○	○
Swollen hands or feet	○	○	○	○	○
Swollen lymph nodes	○	○	○	○	○
Syncope (fainting)	○	○	○	○	○
Tachycardia (rapid heartbeat) at rest	○	○	○	○	○
Tachycardia (rapid heartbeat) after standing up	○	○	○	○	○
Thrush (white fungal infection in the mouth or throat)	○	○	○	○	○
Tinnitus or humming in ears	○	○	○	○	○
Tremors or shakiness	○	○	○	○	○
Upper back pain	○	○	○	○	○
UTI (urinary tract infection)	○	○	○	○	○
Weakened neck	○	○	○	○	○

Weight gain	◯	◯	◯	◯	◯

Are there any symptoms not listed above that you have experienced since you received the first dose of your vaccine on [vaccine_arm_1][v_v_vax_date]? __________________________________

*Please list all if so

Since you received the first dose of your vaccine on [vaccine_arm_1][v_v_vax_date]

	76-100% of health before COVID-19	51-75% of health before COVID-19	26-50% of health before COVID-19	0-25% of health before COVID-19
On your best days, would you say that you are at	◯	◯	◯	◯
On your worst days, would you say that you are at	◯	◯	◯	◯

Since you received the first dose of your vaccine on [vaccine_arm_1][v_v_vax_date], would you say that your health is

◯ Better
◯ Worse
◯ The same
◯ Don't know

Please, in the space below, share any information about your experience with COVID-19 or the vaccine that we might have missed.

____

Survey 3

Thank you for taking Survey 3 for the Yale COVID Recovery Study!

Please remember that you may stop this survey and come back to it at any point. To return to the survey, please return to the link in your browser (or reclick the link in your email).

You may also find it helpful to have the below items ready as you complete the survey. If you do not have these items, please still fill in the information as best you can.

Testing results: dates, type (PCR, antigen) and result of tests. If you do not remember the exact date, the estimated date is enough.Symptom time and severity: your symptom log. You will be asked to pick symptoms you have had since you began having COVID-19 symptoms.

Have you received your second dose of the COVID-19 vaccine?	◯ Yes ______<br>◯ No, but it's scheduled<br>◯ No, and I don't have an appointment scheduled
When did you receive your second dose of the COVID-19 vaccine?	______________________________

On your previous survey, you responded that you have experienced the following symptoms due to COVID-19 at some point in the past.

The following questions will ask about how these symptoms have changed, if at all. Please respond based on your experiences during the past month.

During the past month (as compared to before one month ago), this symptom has been	Better	Worse	The same
Abdominal pain	◯	◯	◯
Abnormally low temperature	◯	◯	◯
Acid reflux	◯	◯	◯
Afternoon or evening fevers/low-grade fevers	◯	◯	◯
Anemia (low number of red blood cells)	◯	◯	◯
Anxiety	◯	◯	◯
Arrythmia (improper beating of the heart due to electrical impulse problems)	◯	◯	◯
Bilateral neck throbbing around lymph nodes	◯	◯	◯
Blurry vision	◯	◯	◯
Bone aches in extremities	◯	◯	◯
Brain fog	◯	◯	◯
Brain pressure	◯	◯	◯
Bulging veins	◯	◯	◯
Burning sensations	◯	◯	◯
Bruising of skin	◯	◯	◯
Calf cramps	◯	◯	◯
Change in nails (i.e. white spots, brittleness, change in moons)	◯	◯	◯
Changes in voice	◯	◯	◯
Changed sense of taste	◯	◯	◯
Chills but no fever	◯	◯	◯
Clogged ears	◯	◯	◯
Cold burning feeling in lungs	◯	◯	◯
Confusion	◯	◯	◯
Congested or runny nose	◯	◯	◯
Constant thirst	◯	◯	◯
Costochondritis (inflammation of the cartilage that connects a rib to the breastbone)	◯	◯	◯
Cough	◯	◯	◯
Coughing up blood	◯	◯	◯
Covid toes (tender or itchy rash or chilblains on the toes or foot)	◯	◯	◯

Cracked or dry lips	○	○	○
Dental problems (i.e. chipped tooth, tooth loss)	○	○	○
Diarrhea	○	○	○
Difficulty concentrating or focusing	○	○	○
Difficulty sleeping	○	○	○
Difficulty speaking properly	○	○	○
Discoloration of the skin (for example: purple or blue on the hands or feet, no blistering)	○	○	○
Dizziness	○	○	○
Dry eyes	○	○	○
Dry or peeling skin	○	○	○
Dry scalp or dandruff	○	○	○
Dry throat	○	○	○
Ear pain/earache	○	○	○
Elevated thyroid hormones	○	○	○
Extreme pressure at base of head or occipital nerve	○	○	○
Eye stye or infection	○	○	○
Fatigue	○	○	○
Feeling irritable	○	○	○
Feeling of burning skin	○	○	○
Fever or chills	○	○	○
Floaters or flashes of light in vision	○	○	○
Foot pain	○	○	○
GERD (acid reflux) with excessive salivation	○	○	○
Goiter or lump in throat	○	○	○
Hair loss	○	○	○
Hand or wrist pain	○	○	○
Headache	○	○	○
Heart palpitations (heart skipping a beat or racing)	○	○	○
Heat intolerance	○	○	○
High blood pressure	○	○	○
Hormone imbalances	○	○	○
"Hot" blood rush	○	○	○
Inability to cry	○	○	○

Inability to exercise or be active	○	○	○
Inability to yawn	○	○	○
Internal tremors or buzzing/vibration	○	○	○
Irregular or skipped menstrual cycles	○	○	○
Jaw pain	○	○	○
Joint pain	○	○	○
Kidney issues or protein in urine	○	○	○
Kidney pain	○	○	○
Low blood oxygen	○	○	○
Low blood pressure	○	○	○
Lower back pain	○	○	○
Loss of appetite	○	○	○
Loss of hearing	○	○	○
Loss or decrease in quality of vision	○	○	○
Lump in throat/difficulty swallowing	○	○	○
Memory problems	○	○	○
Menstrual cycles that are heavier or lighter than normal	○	○	○
Mid-back pain at base of ribs	○	○	○
Mouth sores or sore tongue	○	○	○
Muscle or body aches	○	○	○
Muscle twitching	○	○	○
Nausea or vomiting	○	○	○
Neck muscle pain	○	○	○
Nerve pain	○	○	○
Nerve sensations (tingling, pins and needles, numbness)	○	○	○
Neuropathy in feet and hands (weakness, numbness, and pain)	○	○	○
New allergies	○	○	○
Night sweats	○	○	○
Nightmares	○	○	○
Painful scalp	○	○	○
Partial or complete loss of sense of smell	○	○	○
Partial or complete loss of sense of taste	○	○	○

Persistent chest pain or pressure	◯	◯	◯
Personality change (drastic)	◯	◯	◯
Petechiae (pinpoint rash)	◯	◯	◯
Phantom smells	◯	◯	◯
Phlegm in back of throat	◯	◯	◯
Post-exertional malaise (worsened symptoms or flu-like symptoms after exertion)	◯	◯	◯
Postnasal drip	◯	◯	◯
Rash	◯	◯	◯
Reflux or heartburn	◯	◯	◯
Runny nose	◯	◯	◯
Sadness	◯	◯	◯
Seizures	◯	◯	◯
Sharp or sudden chest pain	◯	◯	◯
Shortness of breath or difficulty breathing	◯	◯	◯
Shortness of breath or exhaustion from bending over	◯	◯	◯
Sleeping more than normal	◯	◯	◯
Sore throat	◯	◯	◯
Spinal issues	◯	◯	◯
Spikes in blood pressure	◯	◯	◯
Swollen hands or feet	◯	◯	◯
Swollen lymph nodes	◯	◯	◯
Syncope (fainting)	◯	◯	◯
Tachycardia (rapid heartbeat) at rest	◯	◯	◯
Tachycardia (rapid heartbeat) after standing up	◯	◯	◯
Thrush (white fungal infection in the mouth or throat)	◯	◯	◯
Tinnitus or humming in ears	◯	◯	◯
Tremors or shakiness	◯	◯	◯
Upper back pain	◯	◯	◯
UTI (urinary tract infection)	◯	◯	◯
Weakened neck	◯	◯	◯
Weight gain	◯	◯	◯

During the past month, I have experienced the symptom, on average

	Never--it has gone away completely	Once a month	Once or twice a week	3 or 4 times a week	Most days	All the time
Abdominal pain	○	○	○	○	○	○
Abnormally low temperature	○	○	○	○	○	○
Acid reflux	○	○	○	○	○	○
Afternoon or evening fevers/low-grade fevers	○	○	○	○	○	○
Anemia (low number of red blood cells)	○	○	○	○	○	○
Anxiety	○	○	○	○	○	○
Arrythmia (improper beating of the heart due to electrical impulse problems)	○	○	○	○	○	○
Bilateral neck throbbing around lymph nodes	○	○	○	○	○	○
Blurry vision	○	○	○	○	○	○
Bone aches in extremities	○	○	○	○	○	○
Brain fog	○	○	○	○	○	○
Brain pressure	○	○	○	○	○	○
Bulging veins	○	○	○	○	○	○
Burning sensations	○	○	○	○	○	○
Bruising of skin	○	○	○	○	○	○
Calf cramps	○	○	○	○	○	○
Change in nails (i.e. white spots, brittleness, change in moons)	○	○	○	○	○	○
Changes in voice	○	○	○	○	○	○
Changed sense of taste	○	○	○	○	○	○
Chills but no fever	○	○	○	○	○	○
Clogged ears	○	○	○	○	○	○
Cold burning feeling in lungs	○	○	○	○	○	○
Confusion	○	○	○	○	○	○
Congested or runny nose	○	○	○	○	○	○
Constant thirst	○	○	○	○	○	○
Costochondritis (inflammation of the cartilage that connects a rib to the breastbone)	○	○	○	○	○	○
Cough	○	○	○	○	○	○
Coughing up blood	○	○	○	○	○	○

Covid toes (tender or itchy rash or chilblains on the toes or foot)	○	○	○	○	○	○
Cracked or dry lips	○	○	○	○	○	○
Dental problems (i.e. chipped tooth, tooth loss)	○	○	○	○	○	○
Diarrhea	○	○	○	○	○	○
Difficulty concentrating or focusing	○	○	○	○	○	○
Difficulty sleeping	○	○	○	○	○	○
Difficulty speaking properly	○	○	○	○	○	○
Discoloration of the skin (for example: purple or blue on the hands or feet, no blistering)	○	○	○	○	○	○
Dizziness	○	○	○	○	○	○
Dry eyes	○	○	○	○	○	○
Dry or peeling skin	○	○	○	○	○	○
Dry scalp or dandruff	○	○	○	○	○	○
Dry throat	○	○	○	○	○	○
Ear pain/earache	○	○	○	○	○	○
Elevated thyroid hormones	○	○	○	○	○	○
Extreme pressure at base of head or occipital nerve	○	○	○	○	○	○
Eye stye or infection	○	○	○	○	○	○
Fatigue	○	○	○	○	○	○
Feeling irritable	○	○	○	○	○	○
Feeling of burning skin	○	○	○	○	○	○
Fever or chills	○	○	○	○	○	○
Floaters or flashes of light in vision	○	○	○	○	○	○
Foot pain	○	○	○	○	○	○
GERD (acid reflux) with excessive salivation	○	○	○	○	○	○
Goiter or lump in throat	○	○	○	○	○	○
Hair loss	○	○	○	○	○	○
Hand or wrist pain	○	○	○	○	○	○
Headache	○	○	○	○	○	○
Heart palpitations (heart skipping a beat or racing)	○	○	○	○	○	○
Heat intolerance	○	○	○	○	○	○
High blood pressure	○	○	○	○	○	○
Hormone imbalances	○	○	○	○	○	○

"Hot" blood rush	◯	◯	◯	◯	◯	◯
Inability to cry	◯	◯	◯	◯	◯	◯
Inability to exercise or be active	◯	◯	◯	◯	◯	◯
Inability to yawn	◯	◯	◯	◯	◯	◯
Internal tremors or buzzing/vibration	◯	◯	◯	◯	◯	◯
Irregular or skipped menstrual cycles	◯	◯	◯	◯	◯	◯
Jaw pain	◯	◯	◯	◯	◯	◯
Joint pain	◯	◯	◯	◯	◯	◯
Kidney issues or protein in urine	◯	◯	◯	◯	◯	◯
Kidney pain	◯	◯	◯	◯	◯	◯
Low blood oxygen	◯	◯	◯	◯	◯	◯
Low blood pressure	◯	◯	◯	◯	◯	◯
Lower back pain	◯	◯	◯	◯	◯	◯
Loss of appetite	◯	◯	◯	◯	◯	◯
Loss of hearing	◯	◯	◯	◯	◯	◯
Loss or decrease in quality of vision	◯	◯	◯	◯	◯	◯
Lump in throat/difficulty swallowing	◯	◯	◯	◯	◯	◯
Memory problems	◯	◯	◯	◯	◯	◯
Menstrual cycles that are heavier or lighter than normal	◯	◯	◯	◯	◯	◯
Mid-back pain at base of ribs	◯	◯	◯	◯	◯	◯
Mouth sores or sore tongue	◯	◯	◯	◯	◯	◯
Muscle or body aches	◯	◯	◯	◯	◯	◯
Muscle twitching	◯	◯	◯	◯	◯	◯
Nausea or vomiting	◯	◯	◯	◯	◯	◯
Neck muscle pain	◯	◯	◯	◯	◯	◯
Nerve pain	◯	◯	◯	◯	◯	◯
Nerve sensations (tingling, pins and needles, numbness)	◯	◯	◯	◯	◯	◯
Neuropathy in feet and hands (weakness, numbness, and pain)	◯	◯	◯	◯	◯	◯
New allergies	◯	◯	◯	◯	◯	◯
Night sweats	◯	◯	◯	◯	◯	◯
Nightmares	◯	◯	◯	◯	◯	◯
Painful scalp	◯	◯	◯	◯	◯	◯
Partial or complete loss of sense of smell	◯	◯	◯	◯	◯	◯

Partial or complete loss of sense of taste	○	○	○	○	○	○
Persistent chest pain or pressure	○	○	○	○	○	○
Personality change (drastic)	○	○	○	○	○	○
Petechiae (pinpoint rash)	○	○	○	○	○	○
Phantom smells	○	○	○	○	○	○
Phlegm in back of throat	○	○	○	○	○	○
Post-exertional malaise (worsened symptoms or flu-like symptoms after exertion)	○	○	○	○	○	○
Postnasal drip	○	○	○	○	○	○
Rash	○	○	○	○	○	○
Reflux or heartburn	○	○	○	○	○	○
Runny nose	○	○	○	○	○	○
Sadness	○	○	○	○	○	○
Seizures	○	○	○	○	○	○
Sharp or sudden chest pain	○	○	○	○	○	○
Shortness of breath or difficulty breathing	○	○	○	○	○	○
Shortness of breath or exhaustion from bending over	○	○	○	○	○	○
Sleeping more than normal	○	○	○	○	○	○
Sore throat	○	○	○	○	○	○
Spinal issues	○	○	○	○	○	○
Spikes in blood pressure	○	○	○	○	○	○
Swollen hands or feet	○	○	○	○	○	○
Swollen lymph nodes	○	○	○	○	○	○
Syncope (fainting)	○	○	○	○	○	○
Tachycardia (rapid heartbeat) at rest	○	○	○	○	○	○
Tachycardia (rapid heartbeat) after standing up	○	○	○	○	○	○
Thrush (white fungal infection in the mouth or throat)	○	○	○	○	○	○
Tinnitus or humming in ears	○	○	○	○	○	○
Tremors or shakiness	○	○	○	○	○	○
Upper back pain	○	○	○	○	○	○
UTI (urinary tract infection)	○	○	○	○	○	○
Weakened neck	○	○	○	○	○	○
Weight gain	○	○	○	○	○	○

The next set of questions will ask about the same symptoms. Please answer based on your experience during the past month. If you have not experienced any of the symptoms at all, please select "Not at all" from the choices.

During the past month, while experiencing the symptom, how much did it bothered you in terms of discomfort or pain?

If you have not experienced the symptom during the past month, please choose "Not at all."

	Not at all	A little bit	Somewhat	Quite a bit	Very much
Abdominal pain	◯	◯	◯	◯	◯
Abnormally low temperature	◯	◯	◯	◯	◯
Acid reflux	◯	◯	◯	◯	◯
Afternoon or evening fevers/low-grade fevers	◯	◯	◯	◯	◯
Anemia (low number of red blood cells)	◯	◯	◯	◯	◯
Anxiety	◯	◯	◯	◯	◯
Arrythmia (improper beating of the heart due to electrical impulse problems)	◯	◯	◯	◯	◯
Bilateral neck throbbing around lymph nodes	◯	◯	◯	◯	◯
Blurry vision	◯	◯	◯	◯	◯
Bone aches in extremities	◯	◯	◯	◯	◯
Brain fog	◯	◯	◯	◯	◯
Brain pressure	◯	◯	◯	◯	◯
Bulging veins	◯	◯	◯	◯	◯
Burning sensations	◯	◯	◯	◯	◯
Bruising of skin	◯	◯	◯	◯	◯
Calf cramps	◯	◯	◯	◯	◯
Change in nails (i.e. white spots, brittleness, change in moons)	◯	◯	◯	◯	◯
Changes in voice	◯	◯	◯	◯	◯
Changed sense of taste	◯	◯	◯	◯	◯
Chills but no fever	◯	◯	◯	◯	◯
Clogged ears	◯	◯	◯	◯	◯
Cold burning feeling in lungs	◯	◯	◯	◯	◯
Confusion	◯	◯	◯	◯	◯
Congested or runny nose	◯	◯	◯	◯	◯
Constant thirst	◯	◯	◯	◯	◯
Costochondritis (inflammation of the cartilage that connects a rib to the breastbone)	◯	◯	◯	◯	◯
Cough	◯	◯	◯	◯	◯

Coughing up blood	◯	◯	◯	◯	◯
Covid toes (tender or itchy rash or chilblains on the toes or foot)	◯	◯	◯	◯	◯
Cracked or dry lips	◯	◯	◯	◯	◯
Dental problems (i.e. chipped tooth, tooth loss)	◯	◯	◯	◯	◯
Diarrhea	◯	◯	◯	◯	◯
Difficulty concentrating or focusing	◯	◯	◯	◯	◯
Difficulty sleeping	◯	◯	◯	◯	◯
Difficulty speaking properly	◯	◯	◯	◯	◯
Discoloration of the skin (for example: purple or blue on the hands or feet, no blistering)	◯	◯	◯	◯	◯
Dizziness	◯	◯	◯	◯	◯
Dry eyes	◯	◯	◯	◯	◯
Dry or peeling skin	◯	◯	◯	◯	◯
Dry scalp or dandruff	◯	◯	◯	◯	◯
Dry throat	◯	◯	◯	◯	◯
Ear pain/earache	◯	◯	◯	◯	◯
Elevated thyroid hormones	◯	◯	◯	◯	◯
Extreme pressure at base of head or occipital nerve	◯	◯	◯	◯	◯
Eye stye or infection	◯	◯	◯	◯	◯
Fatigue	◯	◯	◯	◯	◯
Feeling irritable	◯	◯	◯	◯	◯
Feeling of burning skin	◯	◯	◯	◯	◯
Fever or chills	◯	◯	◯	◯	◯
Floaters or flashes of light in vision	◯	◯	◯	◯	◯
Foot pain	◯	◯	◯	◯	◯
GERD (acid reflux) with excessive salivation	◯	◯	◯	◯	◯
Goiter or lump in throat	◯	◯	◯	◯	◯
Hair loss	◯	◯	◯	◯	◯
Hand or wrist pain	◯	◯	◯	◯	◯
Headache	◯	◯	◯	◯	◯
Heart palpitations (heart skipping a beat or racing)	◯	◯	◯	◯	◯
Heat intolerance	◯	◯	◯	◯	◯
High blood pressure	◯	◯	◯	◯	◯

Hormone imbalances	○	○	○	○	○
"Hot" blood rush	○	○	○	○	○
Inability to cry	○	○	○	○	○
Inability to exercise or be active	○	○	○	○	○
Inability to yawn	○	○	○	○	○
Internal tremors or buzzing/vibration	○	○	○	○	○
Irregular or skipped menstrual cycles	○	○	○	○	○
Jaw pain	○	○	○	○	○
Joint pain	○	○	○	○	○
Kidney issues or protein in urine	○	○	○	○	○
Kidney pain	○	○	○	○	○
Low blood oxygen	○	○	○	○	○
Low blood pressure	○	○	○	○	○
Lower back pain	○	○	○	○	○
Loss of appetite	○	○	○	○	○
Loss of hearing	○	○	○	○	○
Loss or decrease in quality of vision	○	○	○	○	○
Lump in throat/difficulty swallowing	○	○	○	○	○
Memory problems	○	○	○	○	○
Menstrual cycles that are heavier or lighter than normal	○	○	○	○	○
Mid-back pain at base of ribs	○	○	○	○	○
Mouth sores or sore tongue	○	○	○	○	○
Muscle or body aches	○	○	○	○	○
Muscle twitching	○	○	○	○	○
Nausea or vomiting	○	○	○	○	○
Neck muscle pain	○	○	○	○	○
Nerve pain	○	○	○	○	○
Nerve sensations (tingling, pins and needles, numbness)	○	○	○	○	○
Neuropathy in feet and hands (weakness, numbness, and pain)	○	○	○	○	○
New allergies	○	○	○	○	○
Night sweats	○	○	○	○	○
Nightmares	○	○	○	○	○
Painful scalp	○	○	○	○	○

Partial or complete loss of sense of smell	○	○	○	○	○
Partial or complete loss of sense of taste	○	○	○	○	○
Persistent chest pain or pressure	○	○	○	○	○
Personality change (drastic)	○	○	○	○	○
Petechiae (pinpoint rash)	○	○	○	○	○
Phantom smells	○	○	○	○	○
Phlegm in back of throat	○	○	○	○	○
Post-exertional malaise (worsened symptoms or flu-like symptoms after exertion)	○	○	○	○	○
Postnasal drip	○	○	○	○	○
Rash	○	○	○	○	○
Reflux or heartburn	○	○	○	○	○
Runny nose	○	○	○	○	○
Sadness	○	○	○	○	○
Seizures	○	○	○	○	○
Sharp or sudden chest pain	○	○	○	○	○
Shortness of breath or difficulty breathing	○	○	○	○	○
Shortness of breath or exhaustion from bending over	○	○	○	○	○
Sleeping more than normal	○	○	○	○	○
Sore throat	○	○	○	○	○
Spinal issues	○	○	○	○	○
Spikes in blood pressure	○	○	○	○	○
Swollen hands or feet	○	○	○	○	○
Swollen lymph nodes	○	○	○	○	○
Syncope (fainting)	○	○	○	○	○
Tachycardia (rapid heartbeat) at rest	○	○	○	○	○
Tachycardia (rapid heartbeat) after standing up	○	○	○	○	○
Thrush (white fungal infection in the mouth or throat)	○	○	○	○	○
Tinnitus or humming in ears	○	○	○	○	○
Tremors or shakiness	○	○	○	○	○
Upper back pain	○	○	○	○	○
UTI (urinary tract infection)	○	○	○	○	○
Weakened neck	○	○	○	○	○

Weight gain	○	○	○	○	○

During the past month, how much did the symptom impair your ability to work compared to pre-COVID?

If you have not experienced the symptom during the past month, please choose "Not at all."

	Not at all	A little bit	Somewhat	Quite a bit	Very much
Abdominal pain	○	○	○	○	○
Abnormally low temperature	○	○	○	○	○
Acid reflux	○	○	○	○	○
Afternoon or evening fevers/low-grade fevers	○	○	○	○	○
Anemia (low number of red blood cells)	○	○	○	○	○
Anxiety	○	○	○	○	○
Arrythmia (improper beating of the heart due to electrical impulse problems)	○	○	○	○	○
Bilateral neck throbbing around lymph nodes	○	○	○	○	○
Blurry vision	○	○	○	○	○
Bone aches in extremities	○	○	○	○	○
Brain fog	○	○	○	○	○
Brain pressure	○	○	○	○	○
Bulging veins	○	○	○	○	○
Burning sensations	○	○	○	○	○
Bruising of skin	○	○	○	○	○
Calf cramps	○	○	○	○	○
Change in nails (i.e. white spots, brittleness, change in moons)	○	○	○	○	○
Changes in voice	○	○	○	○	○
Changed sense of taste	○	○	○	○	○
Chills but no fever	○	○	○	○	○
Clogged ears	○	○	○	○	○
Cold burning feeling in lungs	○	○	○	○	○
Confusion	○	○	○	○	○
Congested or runny nose	○	○	○	○	○
Constant thirst	○	○	○	○	○
Costochondritis (inflammation of the cartilage that connects a rib to the breastbone)	○	○	○	○	○
Cough	○	○	○	○	○

Coughing up blood	○	○	○	○	○
Covid toes (tender or itchy rash or chilblains on the toes or foot)	○	○	○	○	○
Cracked or dry lips	○	○	○	○	○
Dental problems (i.e. chipped tooth, tooth loss)	○	○	○	○	○
Diarrhea	○	○	○	○	○
Difficulty concentrating or focusing	○	○	○	○	○
Difficulty sleeping	○	○	○	○	○
Difficulty speaking properly	○	○	○	○	○
Discoloration of the skin (for example: purple or blue on the hands or feet, no blistering)	○	○	○	○	○
Dizziness	○	○	○	○	○
Dry eyes	○	○	○	○	○
Dry or peeling skin	○	○	○	○	○
Dry scalp or dandruff	○	○	○	○	○
Dry throat	○	○	○	○	○
Ear pain/earache	○	○	○	○	○
Elevated thyroid hormones	○	○	○	○	○
Extreme pressure at base of head or occipital nerve	○	○	○	○	○
Eye stye or infection	○	○	○	○	○
Fatigue	○	○	○	○	○
Feeling irritable	○	○	○	○	○
Feeling of burning skin	○	○	○	○	○
Fever or chills	○	○	○	○	○
Floaters or flashes of light in vision	○	○	○	○	○
Foot pain	○	○	○	○	○
GERD (acid reflux) with excessive salivation	○	○	○	○	○
Goiter or lump in throat	○	○	○	○	○
Hair loss	○	○	○	○	○
Hand or wrist pain	○	○	○	○	○
Headache	○	○	○	○	○
Heart palpitations (heart skipping a beat or racing)	○	○	○	○	○
Heat intolerance	○	○	○	○	○
High blood pressure	○	○	○	○	○

Hormone imbalances	◯	◯	◯	◯	◯
"Hot" blood rush	◯	◯	◯	◯	◯
Inability to cry	◯	◯	◯	◯	◯
Inability to exercise or be active	◯	◯	◯	◯	◯
Inability to yawn	◯	◯	◯	◯	◯
Internal tremors or buzzing/vibration	◯	◯	◯	◯	◯
Irregular or skipped menstrual cycles	◯	◯	◯	◯	◯
Jaw pain	◯	◯	◯	◯	◯
Joint pain	◯	◯	◯	◯	◯
Kidney issues or protein in urine	◯	◯	◯	◯	◯
Kidney pain	◯	◯	◯	◯	◯
Low blood oxygen	◯	◯	◯	◯	◯
Low blood pressure	◯	◯	◯	◯	◯
Lower back pain	◯	◯	◯	◯	◯
Loss of appetite	◯	◯	◯	◯	◯
Loss of hearing	◯	◯	◯	◯	◯
Loss or decrease in quality of vision	◯	◯	◯	◯	◯
Lump in throat/difficulty swallowing	◯	◯	◯	◯	◯
Memory problems	◯	◯	◯	◯	◯
Menstrual cycles that are heavier or lighter than normal	◯	◯	◯	◯	◯
Mid-back pain at base of ribs	◯	◯	◯	◯	◯
Mouth sores or sore tongue	◯	◯	◯	◯	◯
Muscle or body aches	◯	◯	◯	◯	◯
Muscle twitching	◯	◯	◯	◯	◯
Nausea or vomiting	◯	◯	◯	◯	◯
Neck muscle pain	◯	◯	◯	◯	◯
Nerve pain	◯	◯	◯	◯	◯
Nerve sensations (tingling, pins and needles, numbness)	◯	◯	◯	◯	◯
Neuropathy in feet and hands (weakness, numbness, and pain)	◯	◯	◯	◯	◯
New allergies	◯	◯	◯	◯	◯
Night sweats	◯	◯	◯	◯	◯
Nightmares	◯	◯	◯	◯	◯
Painful scalp	◯	◯	◯	◯	◯

Partial or complete loss of sense of smell	○	○	○	○	○
Partial or complete loss of sense of taste	○	○	○	○	○
Persistent chest pain or pressure	○	○	○	○	○
Personality change (drastic)	○	○	○	○	○
Petechiae (pinpoint rash)	○	○	○	○	○
Phantom smells	○	○	○	○	○
Phlegm in back of throat	○	○	○	○	○
Post-exertional malaise (worsened symptoms or flu-like symptoms after exertion)	○	○	○	○	○
Postnasal drip	○	○	○	○	○
Rash	○	○	○	○	○
Reflux or heartburn	○	○	○	○	○
Runny nose	○	○	○	○	○
Sadness	○	○	○	○	○
Seizures	○	○	○	○	○
Sharp or sudden chest pain	○	○	○	○	○
Shortness of breath or difficulty breathing	○	○	○	○	○
Shortness of breath or exhaustion from bending over	○	○	○	○	○
Sleeping more than normal	○	○	○	○	○
Sore throat	○	○	○	○	○
Spinal issues	○	○	○	○	○
Spikes in blood pressure	○	○	○	○	○
Swollen hands or feet	○	○	○	○	○
Swollen lymph nodes	○	○	○	○	○
Syncope (fainting)	○	○	○	○	○
Tachycardia (rapid heartbeat) at rest	○	○	○	○	○
Tachycardia (rapid heartbeat) after standing up	○	○	○	○	○
Thrush (white fungal infection in the mouth or throat)	○	○	○	○	○
Tinnitus or humming in ears	○	○	○	○	○
Tremors or shakiness	○	○	○	○	○
Upper back pain	○	○	○	○	○
UTI (urinary tract infection)	○	○	○	○	○
Weakened neck	○	○	○	○	○

Weight gain	○	○	○	○	○

During the past month, how much did the symptom impair your social or family functioning compared to pre-COVID?

If you have not experienced the symptom during the past month, please choose "Not at all."

	Not at all	A little bit	Somewhat	Quite a bit	Very much
Abdominal pain	◯	◯	◯	◯	◯
Abnormally low temperature	◯	◯	◯	◯	◯
Acid reflux	◯	◯	◯	◯	◯
Afternoon or evening fevers/low-grade fevers	◯	◯	◯	◯	◯
Anemia (low number of red blood cells)	◯	◯	◯	◯	◯
Anxiety	◯	◯	◯	◯	◯
Arrythmia (improper beating of the heart due to electrical impulse problems)	◯	◯	◯	◯	◯
Bilateral neck throbbing around lymph nodes	◯	◯	◯	◯	◯
Blurry vision	◯	◯	◯	◯	◯
Bone aches in extremities	◯	◯	◯	◯	◯
Brain fog	◯	◯	◯	◯	◯
Brain pressure	◯	◯	◯	◯	◯
Bulging veins	◯	◯	◯	◯	◯
Burning sensations	◯	◯	◯	◯	◯
Bruising of skin	◯	◯	◯	◯	◯
Calf cramps	◯	◯	◯	◯	◯
Change in nails (i.e. white spots, brittleness, change in moons)	◯	◯	◯	◯	◯
Changes in voice	◯	◯	◯	◯	◯
Changed sense of taste	◯	◯	◯	◯	◯
Chills but no fever	◯	◯	◯	◯	◯
Clogged ears	◯	◯	◯	◯	◯
Cold burning feeling in lungs	◯	◯	◯	◯	◯
Confusion	◯	◯	◯	◯	◯
Congested or runny nose	◯	◯	◯	◯	◯
Constant thirst	◯	◯	◯	◯	◯
Costochondritis (inflammation of the cartilage that connects a rib to the breastbone)	◯	◯	◯	◯	◯
Cough	◯	◯	◯	◯	◯

Coughing up blood	○	○	○	○	○
Covid toes (tender or itchy rash or chilblains on the toes or foot)	○	○	○	○	○
Cracked or dry lips	○	○	○	○	○
Dental problems (i.e. chipped tooth, tooth loss)	○	○	○	○	○
Diarrhea	○	○	○	○	○
Difficulty concentrating or focusing	○	○	○	○	○
Difficulty sleeping	○	○	○	○	○
Difficulty speaking properly	○	○	○	○	○
Discoloration of the skin (for example: purple or blue on the hands or feet, no blistering)	○	○	○	○	○
Dizziness	○	○	○	○	○
Dry eyes	○	○	○	○	○
Dry or peeling skin	○	○	○	○	○
Dry scalp or dandruff	○	○	○	○	○
Dry throat	○	○	○	○	○
Ear pain/earache	○	○	○	○	○
Elevated thyroid hormones	○	○	○	○	○
Extreme pressure at base of head or occipital nerve	○	○	○	○	○
Eye stye or infection	○	○	○	○	○
Fatigue	○	○	○	○	○
Feeling irritable	○	○	○	○	○
Feeling of burning skin	○	○	○	○	○
Fever or chills	○	○	○	○	○
Floaters or flashes of light in vision	○	○	○	○	○
Foot pain	○	○	○	○	○
GERD (acid reflux) with excessive salivation	○	○	○	○	○
Goiter or lump in throat	○	○	○	○	○
Hair loss	○	○	○	○	○
Hand or wrist pain	○	○	○	○	○
Headache	○	○	○	○	○
Heart palpitations (heart skipping a beat or racing)	○	○	○	○	○
Heat intolerance	○	○	○	○	○
High blood pressure	○	○	○	○	○

Hormone imbalances	◯	◯	◯	◯	◯
"Hot" blood rush	◯	◯	◯	◯	◯
Inability to cry	◯	◯	◯	◯	◯
Inability to exercise or be active	◯	◯	◯	◯	◯
Inability to yawn	◯	◯	◯	◯	◯
Internal tremors or buzzing/vibration	◯	◯	◯	◯	◯
Irregular or skipped menstrual cycles	◯	◯	◯	◯	◯
Jaw pain	◯	◯	◯	◯	◯
Joint pain	◯	◯	◯	◯	◯
Kidney issues or protein in urine	◯	◯	◯	◯	◯
Kidney pain	◯	◯	◯	◯	◯
Low blood oxygen	◯	◯	◯	◯	◯
Low blood pressure	◯	◯	◯	◯	◯
Lower back pain	◯	◯	◯	◯	◯
Loss of appetite	◯	◯	◯	◯	◯
Loss of hearing	◯	◯	◯	◯	◯
Loss or decrease in quality of vision	◯	◯	◯	◯	◯
Lump in throat/difficulty swallowing	◯	◯	◯	◯	◯
Memory problems	◯	◯	◯	◯	◯
Menstrual cycles that are heavier or lighter than normal	◯	◯	◯	◯	◯
Mid-back pain at base of ribs	◯	◯	◯	◯	◯
Mouth sores or sore tongue	◯	◯	◯	◯	◯
Muscle or body aches	◯	◯	◯	◯	◯
Muscle twitching	◯	◯	◯	◯	◯
Nausea or vomiting	◯	◯	◯	◯	◯
Neck muscle pain	◯	◯	◯	◯	◯
Nerve pain	◯	◯	◯	◯	◯
Nerve sensations (tingling, pins and needles, numbness)	◯	◯	◯	◯	◯
Neuropathy in feet and hands (weakness, numbness, and pain)	◯	◯	◯	◯	◯
New allergies	◯	◯	◯	◯	◯
Night sweats	◯	◯	◯	◯	◯
Nightmares	◯	◯	◯	◯	◯
Painful scalp	◯	◯	◯	◯	◯

Partial or complete loss of sense of smell	◯	◯	◯	◯	◯
Partial or complete loss of sense of taste	◯	◯	◯	◯	◯
Persistent chest pain or pressure	◯	◯	◯	◯	◯
Personality change (drastic)	◯	◯	◯	◯	◯
Petechiae (pinpoint rash)	◯	◯	◯	◯	◯
Phantom smells	◯	◯	◯	◯	◯
Phlegm in back of throat	◯	◯	◯	◯	◯
Post-exertional malaise (worsened symptoms or flu-like symptoms after exertion)	◯	◯	◯	◯	◯
Postnasal drip	◯	◯	◯	◯	◯
Rash	◯	◯	◯	◯	◯
Reflux or heartburn	◯	◯	◯	◯	◯
Runny nose	◯	◯	◯	◯	◯
Sadness	◯	◯	◯	◯	◯
Seizures	◯	◯	◯	◯	◯
Sharp or sudden chest pain	◯	◯	◯	◯	◯
Shortness of breath or difficulty breathing	◯	◯	◯	◯	◯
Shortness of breath or exhaustion from bending over	◯	◯	◯	◯	◯
Sleeping more than normal	◯	◯	◯	◯	◯
Sore throat	◯	◯	◯	◯	◯
Spinal issues	◯	◯	◯	◯	◯
Spikes in blood pressure	◯	◯	◯	◯	◯
Swollen hands or feet	◯	◯	◯	◯	◯
Swollen lymph nodes	◯	◯	◯	◯	◯
Syncope (fainting)	◯	◯	◯	◯	◯
Tachycardia (rapid heartbeat) at rest	◯	◯	◯	◯	◯
Tachycardia (rapid heartbeat) after standing up	◯	◯	◯	◯	◯
Thrush (white fungal infection in the mouth or throat)	◯	◯	◯	◯	◯
Tinnitus or humming in ears	◯	◯	◯	◯	◯
Tremors or shakiness	◯	◯	◯	◯	◯
Upper back pain	◯	◯	◯	◯	◯
UTI (urinary tract infection)	◯	◯	◯	◯	◯
Weakened neck	◯	◯	◯	◯	◯

Weight gain	○	○	○	○	○

Are there any symptoms not listed above that you have experienced within the past month?

*Please list all if so

____

Have you visited the hospital or been hospitalized in the past month for COVID-19 symptoms?

○ Yes ______
○ No

Please elaborate on your hospitalization (cause of hospitalization; relation, if any, to COVID-19; and health since hospitalization).

____

During the past month

	76-100% of health before COVID-19	51-75% of health before COVID-19	26-50% of health before COVID-19	0-25% of health before COVID-19
On your best days, would you say that you are at	◯	◯	◯	◯
On your worst days, would you say that you are at	◯	◯	◯	◯

Compared to your health before you received the first dose of your vaccine on [vaccine_arm_1][v_v_vax_date], would you say that your health is currently

◯ Better
◯ Worse
◯ The same
◯ Don't know

Please, in the space below, share any information about your experience with COVID-19 or the vaccine that we might have missed.

____

Survey 4

Thank you for taking Survey 4, the final survey for the Yale COVID Recovery Study!

Please remember that you may stop this survey and come back to it at any point.

You may also find it helpful to have the below items ready as you complete the survey. If you do not have these items, please still fill in the information as best you can.

Testing results: dates, type (PCR, antigen) and result of tests. If you do not remember the exact date, the estimated date is enough.Symptom time and severity: your symptom log. You will be asked to pick symptoms you have had since you began having COVID-19 symptoms.

During the past 6 weeks, have you visited the hospital or been hospitalized for COVID-19 symptoms?

◯ Yes ______
◯ No

What were you hospitalized for?

____

On your previous survey, you responded that you have experienced the following symptoms due to COVID-19 at some point in the past.

The following questions will ask about how these symptoms have changed, if at all. Please respond based on your experiences during the past 6 weeks.

During the past 6 weeks, as compared to before, this symptom has been

	Better	Worse	The same
Abdominal pain	◯	◯	◯
Abnormally low temperature	◯	◯	◯
Acid reflux	◯	◯	◯
Afternoon or evening fevers/low-grade fevers	◯	◯	◯
Anemia (low number of red blood cells)	◯	◯	◯
Anxiety	◯	◯	◯
Arrythmia (improper beating of the heart due to electrical impulse problems)	◯	◯	◯
Bilateral neck throbbing around lymph nodes	◯	◯	◯
Blurry vision	◯	◯	◯
Bone aches in extremities	◯	◯	◯
Brain fog	◯	◯	◯
Brain pressure	◯	◯	◯
Bulging veins	◯	◯	◯
Burning sensations	◯	◯	◯
Bruising of skin	◯	◯	◯
Calf cramps	◯	◯	◯
Change in nails (i.e. white spots, brittleness, change in moons)	◯	◯	◯
Changes in voice	◯	◯	◯
Changed sense of taste	◯	◯	◯
Chills but no fever	◯	◯	◯
Clogged ears	◯	◯	◯
Cold burning feeling in lungs	◯	◯	◯
Confusion	◯	◯	◯
Congested or runny nose	◯	◯	◯
Constant thirst	◯	◯	◯
Costochondritis (inflammation of the cartilage that connects a rib to the breastbone)	◯	◯	◯
Cough	◯	◯	◯
Coughing up blood	◯	◯	◯
Covid toes (tender or itchy rash or chilblains on the toes or foot)	◯	◯	◯

Cracked or dry lips	◯	◯	◯
Dental problems (i.e. chipped tooth, tooth loss)	◯	◯	◯
Diarrhea	◯	◯	◯
Difficulty concentrating or focusing	◯	◯	◯
Difficulty sleeping	◯	◯	◯
Difficulty speaking properly	◯	◯	◯
Discoloration of the skin (for example: purple or blue on the hands or feet, no blistering)	◯	◯	◯
Dizziness	◯	◯	◯
Dry eyes	◯	◯	◯
Dry or peeling skin	◯	◯	◯
Dry scalp or dandruff	◯	◯	◯
Dry throat	◯	◯	◯
Ear pain/earache	◯	◯	◯
Elevated thyroid hormones	◯	◯	◯
Extreme pressure at base of head or occipital nerve	◯	◯	◯
Eye stye or infection	◯	◯	◯
Fatigue	◯	◯	◯
Feeling irritable	◯	◯	◯
Feeling of burning skin	◯	◯	◯
Fever or chills	◯	◯	◯
Floaters or flashes of light in vision	◯	◯	◯
Foot pain	◯	◯	◯
GERD (acid reflux) with excessive salivation	◯	◯	◯
Goiter or lump in throat	◯	◯	◯
Hair loss	◯	◯	◯
Hand or wrist pain	◯	◯	◯
Headache	◯	◯	◯
Heart palpitations (heart skipping a beat or racing)	◯	◯	◯
Heat intolerance	◯	◯	◯
High blood pressure	◯	◯	◯
Hormone imbalances	◯	◯	◯
"Hot" blood rush	◯	◯	◯
Inability to cry	◯	◯	◯

Inability to exercise or be active	◯	◯	◯
Inability to yawn	◯	◯	◯
Internal tremors or buzzing/vibration	◯	◯	◯
Irregular or skipped menstrual cycles	◯	◯	◯
Jaw pain	◯	◯	◯
Joint pain	◯	◯	◯
Kidney issues or protein in urine	◯	◯	◯
Kidney pain	◯	◯	◯
Low blood oxygen	◯	◯	◯
Low blood pressure	◯	◯	◯
Lower back pain	◯	◯	◯
Loss of appetite	◯	◯	◯
Loss of hearing	◯	◯	◯
Loss or decrease in quality of vision	◯	◯	◯
Lump in throat/difficulty swallowing	◯	◯	◯
Memory problems	◯	◯	◯
Menstrual cycles that are heavier or lighter than normal	◯	◯	◯
Mid-back pain at base of ribs	◯	◯	◯
Mouth sores or sore tongue	◯	◯	◯
Muscle or body aches	◯	◯	◯
Muscle twitching	◯	◯	◯
Nausea or vomiting	◯	◯	◯
Neck muscle pain	◯	◯	◯
Nerve pain	◯	◯	◯
Nerve sensations (tingling, pins and needles, numbness)	◯	◯	◯
Neuropathy in feet and hands (weakness, numbness, and pain)	◯	◯	◯
New allergies	◯	◯	◯
Night sweats	◯	◯	◯
Nightmares	◯	◯	◯
Painful scalp	◯	◯	◯
Partial or complete loss of sense of smell	◯	◯	◯
Partial or complete loss of sense of taste	◯	◯	◯

Persistent chest pain or pressure	○	○	○
Personality change (drastic)	○	○	○
Petechiae (pinpoint rash)	○	○	○
Phantom smells	○	○	○
Phlegm in back of throat	○	○	○
Post-exertional malaise (worsened symptoms or flu-like symptoms after exertion)	○	○	○
Postnasal drip	○	○	○
Rash	○	○	○
Reflux or heartburn	○	○	○
Runny nose	○	○	○
Sadness	○	○	○
Seizures	○	○	○
Sharp or sudden chest pain	○	○	○
Shortness of breath or difficulty breathing	○	○	○
Shortness of breath or exhaustion from bending over	○	○	○
Sleeping more than normal	○	○	○
Sore throat	○	○	○
Spinal issues	○	○	○
Spikes in blood pressure	○	○	○
Swollen hands or feet	○	○	○
Swollen lymph nodes	○	○	○
Syncope (fainting)	○	○	○
Tachycardia (rapid heartbeat) at rest	○	○	○
Tachycardia (rapid heartbeat) after standing up	○	○	○
Thrush (white fungal infection in the mouth or throat)	○	○	○
Tinnitus or humming in ears	○	○	○
Tremors or shakiness	○	○	○
Upper back pain	○	○	○
UTI (urinary tract infection)	○	○	○
Weakened neck	○	○	○
Weight gain	○	○	○

During the past 6 weeks, I have experienced this symptom, on average

	Never--it has gone away completely	Once a month	Once or twice a week	3 or 4 times a week	Most days	All the time
Abdominal pain	◯	◯	◯	◯	◯	◯
Abnormally low temperature	◯	◯	◯	◯	◯	◯
Acid reflux	◯	◯	◯	◯	◯	◯
Afternoon or evening fevers/low-grade fevers	◯	◯	◯	◯	◯	◯
Anemia (low number of red blood cells)	◯	◯	◯	◯	◯	◯
Anxiety	◯	◯	◯	◯	◯	◯
Arrythmia (improper beating of the heart due to electrical impulse problems)	◯	◯	◯	◯	◯	◯
Bilateral neck throbbing around lymph nodes	◯	◯	◯	◯	◯	◯
Blurry vision	◯	◯	◯	◯	◯	◯
Bone aches in extremities	◯	◯	◯	◯	◯	◯
Brain fog	◯	◯	◯	◯	◯	◯
Brain pressure	◯	◯	◯	◯	◯	◯
Bulging veins	◯	◯	◯	◯	◯	◯
Burning sensations	◯	◯	◯	◯	◯	◯
Bruising of skin	◯	◯	◯	◯	◯	◯
Calf cramps	◯	◯	◯	◯	◯	◯
Change in nails (i.e. white spots, brittleness, change in moons)	◯	◯	◯	◯	◯	◯
Changes in voice	◯	◯	◯	◯	◯	◯
Changed sense of taste	◯	◯	◯	◯	◯	◯
Chills but no fever	◯	◯	◯	◯	◯	◯
Clogged ears	◯	◯	◯	◯	◯	◯
Cold burning feeling in lungs	◯	◯	◯	◯	◯	◯
Confusion	◯	◯	◯	◯	◯	◯
Congested or runny nose	◯	◯	◯	◯	◯	◯
Constant thirst	◯	◯	◯	◯	◯	◯
Costochondritis (inflammation of the cartilage that connects a rib to the breastbone)	◯	◯	◯	◯	◯	◯
Cough	◯	◯	◯	◯	◯	◯
Coughing up blood	◯	◯	◯	◯	◯	◯

Covid toes (tender or itchy rash or chilblains on the toes or foot)	○	○	○	○	○	○
Cracked or dry lips	○	○	○	○	○	○
Dental problems (i.e. chipped tooth, tooth loss)	○	○	○	○	○	○
Diarrhea	○	○	○	○	○	○
Difficulty concentrating or focusing	○	○	○	○	○	○
Difficulty sleeping	○	○	○	○	○	○
Difficulty speaking properly	○	○	○	○	○	○
Discoloration of the skin (for example: purple or blue on the hands or feet, no blistering)	○	○	○	○	○	○
Dizziness	○	○	○	○	○	○
Dry eyes	○	○	○	○	○	○
Dry or peeling skin	○	○	○	○	○	○
Dry scalp or dandruff	○	○	○	○	○	○
Dry throat	○	○	○	○	○	○
Ear pain/earache	○	○	○	○	○	○
Elevated thyroid hormones	○	○	○	○	○	○
Extreme pressure at base of head or occipital nerve	○	○	○	○	○	○
Eye stye or infection	○	○	○	○	○	○
Fatigue	○	○	○	○	○	○
Feeling irritable	○	○	○	○	○	○
Feeling of burning skin	○	○	○	○	○	○
Fever or chills	○	○	○	○	○	○
Floaters or flashes of light in vision	○	○	○	○	○	○
Foot pain	○	○	○	○	○	○
GERD (acid reflux) with excessive salivation	○	○	○	○	○	○
Goiter or lump in throat	○	○	○	○	○	○
Hair loss	○	○	○	○	○	○
Hand or wrist pain	○	○	○	○	○	○
Headache	○	○	○	○	○	○
Heart palpitations (heart skipping a beat or racing)	○	○	○	○	○	○
Heat intolerance	○	○	○	○	○	○
High blood pressure	○	○	○	○	○	○
Hormone imbalances	○	○	○	○	○	○

"Hot" blood rush	◯	◯	◯	◯	◯	◯
Inability to cry	◯	◯	◯	◯	◯	◯
Inability to exercise or be active	◯	◯	◯	◯	◯	◯
Inability to yawn	◯	◯	◯	◯	◯	◯
Internal tremors or buzzing/vibration	◯	◯	◯	◯	◯	◯
Irregular or skipped menstrual cycles	◯	◯	◯	◯	◯	◯
Jaw pain	◯	◯	◯	◯	◯	◯
Joint pain	◯	◯	◯	◯	◯	◯
Kidney issues or protein in urine	◯	◯	◯	◯	◯	◯
Kidney pain	◯	◯	◯	◯	◯	◯
Low blood oxygen	◯	◯	◯	◯	◯	◯
Low blood pressure	◯	◯	◯	◯	◯	◯
Lower back pain	◯	◯	◯	◯	◯	◯
Loss of appetite	◯	◯	◯	◯	◯	◯
Loss of hearing	◯	◯	◯	◯	◯	◯
Loss or decrease in quality of vision	◯	◯	◯	◯	◯	◯
Lump in throat/difficulty swallowing	◯	◯	◯	◯	◯	◯
Memory problems	◯	◯	◯	◯	◯	◯
Menstrual cycles that are heavier or lighter than normal	◯	◯	◯	◯	◯	◯
Mid-back pain at base of ribs	◯	◯	◯	◯	◯	◯
Mouth sores or sore tongue	◯	◯	◯	◯	◯	◯
Muscle or body aches	◯	◯	◯	◯	◯	◯
Muscle twitching	◯	◯	◯	◯	◯	◯
Nausea or vomiting	◯	◯	◯	◯	◯	◯
Neck muscle pain	◯	◯	◯	◯	◯	◯
Nerve pain	◯	◯	◯	◯	◯	◯
Nerve sensations (tingling, pins and needles, numbness)	◯	◯	◯	◯	◯	◯
Neuropathy in feet and hands (weakness, numbness, and pain)	◯	◯	◯	◯	◯	◯
New allergies	◯	◯	◯	◯	◯	◯
Night sweats	◯	◯	◯	◯	◯	◯
Nightmares	◯	◯	◯	◯	◯	◯
Painful scalp	◯	◯	◯	◯	◯	◯
Partial or complete loss of sense of smell	◯	◯	◯	◯	◯	◯

Partial or complete loss of sense of taste	○	○	○	○	○	○
Persistent chest pain or pressure	○	○	○	○	○	○
Personality change (drastic)	○	○	○	○	○	○
Petechiae (pinpoint rash)	○	○	○	○	○	○
Phantom smells	○	○	○	○	○	○
Phlegm in back of throat	○	○	○	○	○	○
Post-exertional malaise (worsened symptoms or flu-like symptoms after exertion)	○	○	○	○	○	○
Postnasal drip	○	○	○	○	○	○
Rash	○	○	○	○	○	○
Reflux or heartburn	○	○	○	○	○	○
Runny nose	○	○	○	○	○	○
Sadness	○	○	○	○	○	○
Seizures	○	○	○	○	○	○
Sharp or sudden chest pain	○	○	○	○	○	○
Shortness of breath or difficulty breathing	○	○	○	○	○	○
Shortness of breath or exhaustion from bending over	○	○	○	○	○	○
Sleeping more than normal	○	○	○	○	○	○
Sore throat	○	○	○	○	○	○
Spinal issues	○	○	○	○	○	○
Spikes in blood pressure	○	○	○	○	○	○
Swollen hands or feet	○	○	○	○	○	○
Swollen lymph nodes	○	○	○	○	○	○
Syncope (fainting)	○	○	○	○	○	○
Tachycardia (rapid heartbeat) at rest	○	○	○	○	○	○
Tachycardia (rapid heartbeat) after standing up	○	○	○	○	○	○
Thrush (white fungal infection in the mouth or throat)	○	○	○	○	○	○
Tinnitus or humming in ears	○	○	○	○	○	○
Tremors or shakiness	○	○	○	○	○	○
Upper back pain	○	○	○	○	○	○
UTI (urinary tract infection)	○	○	○	○	○	○
Weakened neck	○	○	○	○	○	○
Weight gain	○	○	○	○	○	○

The next set of questions will ask about the same symptoms. Please answer based on your experience during the past 6 weeks. If you have not experienced any of the symptoms at all, please select "Not at all" from the choices.

During the past 6 weeks, while experiencing the symptom, how much did it bother you in terms of discomfort or pain?

***If you have not experienced the symptom at all during the past 6 weeks, please select "Not at all."**

	Not at all	A little bit	Somewhat	Quite a bit	Very much
Abdominal pain	○	○	○	○	○
Abnormally low temperature	○	○	○	○	○
Acid reflux	○	○	○	○	○
Afternoon or evening fevers/low-grade fevers	○	○	○	○	○
Anemia (low number of red blood cells)	○	○	○	○	○
Anxiety	○	○	○	○	○
Arrythmia (improper beating of the heart due to electrical impulse problems)	○	○	○	○	○
Bilateral neck throbbing around lymph nodes	○	○	○	○	○
Blurry vision	○	○	○	○	○
Bone aches in extremities	○	○	○	○	○
Brain fog	○	○	○	○	○
Brain pressure	○	○	○	○	○
Bulging veins	○	○	○	○	○
Burning sensations	○	○	○	○	○
Bruising of skin	○	○	○	○	○
Calf cramps	○	○	○	○	○
Change in nails (i.e. white spots, brittleness, change in moons)	○	○	○	○	○
Changes in voice	○	○	○	○	○
Changed sense of taste	○	○	○	○	○
Chills but no fever	○	○	○	○	○
Clogged ears	○	○	○	○	○
Cold burning feeling in lungs	○	○	○	○	○
Confusion	○	○	○	○	○
Congested or runny nose	○	○	○	○	○
Constant thirst	○	○	○	○	○
Costochondritis (inflammation of the cartilage that connects a rib to the breastbone)	○	○	○	○	○

Cough	○	○	○	○	○
Coughing up blood	○	○	○	○	○
Covid toes (tender or itchy rash or chilblains on the toes or foot)	○	○	○	○	○
Cracked or dry lips	○	○	○	○	○
Dental problems (i.e. chipped tooth, tooth loss)	○	○	○	○	○
Diarrhea	○	○	○	○	○
Difficulty concentrating or focusing	○	○	○	○	○
Difficulty sleeping	○	○	○	○	○
Difficulty speaking properly	○	○	○	○	○
Discoloration of the skin (for example: purple or blue on the hands or feet, no blistering)	○	○	○	○	○
Dizziness	○	○	○	○	○
Dry eyes	○	○	○	○	○
Dry or peeling skin	○	○	○	○	○
Dry scalp or dandruff	○	○	○	○	○
Dry throat	○	○	○	○	○
Ear pain/earache	○	○	○	○	○
Elevated thyroid hormones	○	○	○	○	○
Extreme pressure at base of head or occipital nerve	○	○	○	○	○
Eye stye or infection	○	○	○	○	○
Fatigue	○	○	○	○	○
Feeling irritable	○	○	○	○	○
Feeling of burning skin	○	○	○	○	○
Fever or chills	○	○	○	○	○
Floaters or flashes of light in vision	○	○	○	○	○
Foot pain	○	○	○	○	○
GERD (acid reflux) with excessive salivation	○	○	○	○	○
Goiter or lump in throat	○	○	○	○	○
Hair loss	○	○	○	○	○
Hand or wrist pain	○	○	○	○	○
Headache	○	○	○	○	○
Heart palpitations (heart skipping a beat or racing)	○	○	○	○	○
Heat intolerance	○	○	○	○	○

High blood pressure	○	○	○	○	○
Hormone imbalances	○	○	○	○	○
"Hot" blood rush	○	○	○	○	○
Inability to cry	○	○	○	○	○
Inability to exercise or be active	○	○	○	○	○
Inability to yawn	○	○	○	○	○
Internal tremors or buzzing/vibration	○	○	○	○	○
Irregular or skipped menstrual cycles	○	○	○	○	○
Jaw pain	○	○	○	○	○
Joint pain	○	○	○	○	○
Kidney issues or protein in urine	○	○	○	○	○
Kidney pain	○	○	○	○	○
Low blood oxygen	○	○	○	○	○
Low blood pressure	○	○	○	○	○
Lower back pain	○	○	○	○	○
Loss of appetite	○	○	○	○	○
Loss of hearing	○	○	○	○	○
Loss or decrease in quality of vision	○	○	○	○	○
Lump in throat/difficulty swallowing	○	○	○	○	○
Memory problems	○	○	○	○	○
Menstrual cycles that are heavier or lighter than normal	○	○	○	○	○
Mid-back pain at base of ribs	○	○	○	○	○
Mouth sores or sore tongue	○	○	○	○	○
Muscle or body aches	○	○	○	○	○
Muscle twitching	○	○	○	○	○
Nausea or vomiting	○	○	○	○	○
Neck muscle pain	○	○	○	○	○
Nerve pain	○	○	○	○	○
Nerve sensations (tingling, pins and needles, numbness)	○	○	○	○	○
Neuropathy in feet and hands (weakness, numbness, and pain)	○	○	○	○	○
New allergies	○	○	○	○	○
Night sweats	○	○	○	○	○
Nightmares	○	○	○	○	○
Painful scalp	○	○	○	○	○

Partial or complete loss of sense of smell	○	○	○	○	○
Partial or complete loss of sense of taste	○	○	○	○	○
Persistent chest pain or pressure	○	○	○	○	○
Personality change (drastic)	○	○	○	○	○
Petechiae (pinpoint rash)	○	○	○	○	○
Phantom smells	○	○	○	○	○
Phlegm in back of throat	○	○	○	○	○
Post-exertional malaise (worsened symptoms or flu-like symptoms after exertion)	○	○	○	○	○
Postnasal drip	○	○	○	○	○
Rash	○	○	○	○	○
Reflux or heartburn	○	○	○	○	○
Runny nose	○	○	○	○	○
Sadness	○	○	○	○	○
Seizures	○	○	○	○	○
Sharp or sudden chest pain	○	○	○	○	○
Shortness of breath or difficulty breathing	○	○	○	○	○
Shortness of breath or exhaustion from bending over	○	○	○	○	○
Sleeping more than normal	○	○	○	○	○
Sore throat	○	○	○	○	○
Spinal issues	○	○	○	○	○
Spikes in blood pressure	○	○	○	○	○
Swollen hands or feet	○	○	○	○	○
Swollen lymph nodes	○	○	○	○	○
Syncope (fainting)	○	○	○	○	○
Tachycardia (rapid heartbeat) at rest	○	○	○	○	○
Tachycardia (rapid heartbeat) after standing up	○	○	○	○	○
Thrush (white fungal infection in the mouth or throat)	○	○	○	○	○
Tinnitus or humming in ears	○	○	○	○	○
Tremors or shakiness	○	○	○	○	○
Upper back pain	○	○	○	○	○
UTI (urinary tract infection)	○	○	○	○	○
Weakened neck	○	○	○	○	○

Weight gain	◯	◯	◯	◯	◯

During the past 6 weeks, ow much did the symptom impair your ability to work compared to pre-COVID?

***If you have not experienced the symptom at all during the past 6 weeks, please select "Not at all."**

	Not at all	A little bit	Somewhat	Quite a bit	Very much
Abdominal pain					
Abnormally low temperature					
Acid reflux					
Afternoon or evening fevers/low-grade fevers					
Anemia (low number of red blood cells)					
Anxiety					
Arrythmia (improper beating of the heart due to electrical impulse problems)					
Bilateral neck throbbing around lymph nodes					
Blurry vision					
Bone aches in extremities					
Brain fog					
Brain pressure					
Bulging veins					
Burning sensations					
Bruising of skin					
Calf cramps					
Change in nails (i.e. white spots, brittleness, change in moons)					
Changes in voice					
Changed sense of taste					
Chills but no fever					
Clogged ears					
Cold burning feeling in lungs					
Confusion					
Congested or runny nose					
Constant thirst					
Costochondritis (inflammation of the cartilage that connects a rib to the breastbone)					

Cough	◯	◯	◯	◯	◯
Coughing up blood	◯	◯	◯	◯	◯
Covid toes (tender or itchy rash or chilblains on the toes or foot)	◯	◯	◯	◯	◯
Cracked or dry lips	◯	◯	◯	◯	◯
Dental problems (i.e. chipped tooth, tooth loss)	◯	◯	◯	◯	◯
Diarrhea	◯	◯	◯	◯	◯
Difficulty concentrating or focusing	◯	◯	◯	◯	◯
Difficulty sleeping	◯	◯	◯	◯	◯
Difficulty speaking properly	◯	◯	◯	◯	◯
Discoloration of the skin (for example: purple or blue on the hands or feet, no blistering)	◯	◯	◯	◯	◯
Dizziness	◯	◯	◯	◯	◯
Dry eyes	◯	◯	◯	◯	◯
Dry or peeling skin	◯	◯	◯	◯	◯
Dry scalp or dandruff	◯	◯	◯	◯	◯
Dry throat	◯	◯	◯	◯	◯
Ear pain/earache	◯	◯	◯	◯	◯
Elevated thyroid hormones	◯	◯	◯	◯	◯
Extreme pressure at base of head or occipital nerve	◯	◯	◯	◯	◯
Eye stye or infection	◯	◯	◯	◯	◯
Fatigue	◯	◯	◯	◯	◯
Feeling irritable	◯	◯	◯	◯	◯
Feeling of burning skin	◯	◯	◯	◯	◯
Fever or chills	◯	◯	◯	◯	◯
Floaters or flashes of light in vision	◯	◯	◯	◯	◯
Foot pain	◯	◯	◯	◯	◯
GERD (acid reflux) with excessive salivation	◯	◯	◯	◯	◯
Goiter or lump in throat	◯	◯	◯	◯	◯
Hair loss	◯	◯	◯	◯	◯
Hand or wrist pain	◯	◯	◯	◯	◯
Headache	◯	◯	◯	◯	◯
Heart palpitations (heart skipping a beat or racing)	◯	◯	◯	◯	◯
Heat intolerance	◯	◯	◯	◯	◯

High blood pressure	○	○	○	○	○
Hormone imbalances	○	○	○	○	○
"Hot" blood rush	○	○	○	○	○
Inability to cry	○	○	○	○	○
Inability to exercise or be active	○	○	○	○	○
Inability to yawn	○	○	○	○	○
Internal tremors or buzzing/vibration	○	○	○	○	○
Irregular or skipped menstrual cycles	○	○	○	○	○
Jaw pain	○	○	○	○	○
Joint pain	○	○	○	○	○
Kidney issues or protein in urine	○	○	○	○	○
Kidney pain	○	○	○	○	○
Low blood oxygen	○	○	○	○	○
Low blood pressure	○	○	○	○	○
Lower back pain	○	○	○	○	○
Loss of appetite	○	○	○	○	○
Loss of hearing	○	○	○	○	○
Loss or decrease in quality of vision	○	○	○	○	○
Lump in throat/difficulty swallowing	○	○	○	○	○
Memory problems	○	○	○	○	○
Menstrual cycles that are heavier or lighter than normal	○	○	○	○	○
Mid-back pain at base of ribs	○	○	○	○	○
Mouth sores or sore tongue	○	○	○	○	○
Muscle or body aches	○	○	○	○	○
Muscle twitching	○	○	○	○	○
Nausea or vomiting	○	○	○	○	○
Neck muscle pain	○	○	○	○	○
Nerve pain	○	○	○	○	○
Nerve sensations (tingling, pins and needles, numbness)	○	○	○	○	○
Neuropathy in feet and hands (weakness, numbness, and pain)	○	○	○	○	○
New allergies	○	○	○	○	○
Night sweats	○	○	○	○	○
Nightmares	○	○	○	○	○
Painful scalp	○	○	○	○	○

Partial or complete loss of sense of smell	◯	◯	◯	◯	◯
Partial or complete loss of sense of taste	◯	◯	◯	◯	◯
Persistent chest pain or pressure	◯	◯	◯	◯	◯
Personality change (drastic)	◯	◯	◯	◯	◯
Petechiae (pinpoint rash)	◯	◯	◯	◯	◯
Phantom smells	◯	◯	◯	◯	◯
Phlegm in back of throat	◯	◯	◯	◯	◯
Post-exertional malaise (worsened symptoms or flu-like symptoms after exertion)	◯	◯	◯	◯	◯
Postnasal drip	◯	◯	◯	◯	◯
Rash	◯	◯	◯	◯	◯
Reflux or heartburn	◯	◯	◯	◯	◯
Runny nose	◯	◯	◯	◯	◯
Sadness	◯	◯	◯	◯	◯
Seizures	◯	◯	◯	◯	◯
Sharp or sudden chest pain	◯	◯	◯	◯	◯
Shortness of breath or difficulty breathing	◯	◯	◯	◯	◯
Shortness of breath or exhaustion from bending over	◯	◯	◯	◯	◯
Sleeping more than normal	◯	◯	◯	◯	◯
Sore throat	◯	◯	◯	◯	◯
Spinal issues	◯	◯	◯	◯	◯
Spikes in blood pressure	◯	◯	◯	◯	◯
Swollen hands or feet	◯	◯	◯	◯	◯
Swollen lymph nodes	◯	◯	◯	◯	◯
Syncope (fainting)	◯	◯	◯	◯	◯
Tachycardia (rapid heartbeat) at rest	◯	◯	◯	◯	◯
Tachycardia (rapid heartbeat) after standing up	◯	◯	◯	◯	◯
Thrush (white fungal infection in the mouth or throat)	◯	◯	◯	◯	◯
Tinnitus or humming in ears	◯	◯	◯	◯	◯
Tremors or shakiness	◯	◯	◯	◯	◯
Upper back pain	◯	◯	◯	◯	◯
UTI (urinary tract infection)	◯	◯	◯	◯	◯
Weakened neck	◯	◯	◯	◯	◯

Weight gain	○	○	○	○	○

During the past 6 weeks, how much did the symptom impair your social or family functioning compared to pre-COVID?

***If you have not experienced the symptom at all during the past 6 weeks, please select "Not at all."**

	Not at all	A little bit	Somewhat	Quite a bit	Very much
Abdominal pain	◯	◯	◯	◯	◯
Abnormally low temperature	◯	◯	◯	◯	◯
Acid reflux	◯	◯	◯	◯	◯
Afternoon or evening fevers/low-grade fevers	◯	◯	◯	◯	◯
Anemia (low number of red blood cells)	◯	◯	◯	◯	◯
Anxiety	◯	◯	◯	◯	◯
Arrythmia (improper beating of the heart due to electrical impulse problems)	◯	◯	◯	◯	◯
Bilateral neck throbbing around lymph nodes	◯	◯	◯	◯	◯
Blurry vision	◯	◯	◯	◯	◯
Bone aches in extremities	◯	◯	◯	◯	◯
Brain fog	◯	◯	◯	◯	◯
Brain pressure	◯	◯	◯	◯	◯
Bulging veins	◯	◯	◯	◯	◯
Burning sensations	◯	◯	◯	◯	◯
Bruising of skin	◯	◯	◯	◯	◯
Calf cramps	◯	◯	◯	◯	◯
Change in nails (i.e. white spots, brittleness, change in moons)	◯	◯	◯	◯	◯
Changes in voice	◯	◯	◯	◯	◯
Changed sense of taste	◯	◯	◯	◯	◯
Chills but no fever	◯	◯	◯	◯	◯
Clogged ears	◯	◯	◯	◯	◯
Cold burning feeling in lungs	◯	◯	◯	◯	◯
Confusion	◯	◯	◯	◯	◯
Congested or runny nose	◯	◯	◯	◯	◯
Constant thirst	◯	◯	◯	◯	◯
Costochondritis (inflammation of the cartilage that connects a rib to the breastbone)	◯	◯	◯	◯	◯

Cough	○	○	○	○	○
Coughing up blood	○	○	○	○	○
Covid toes (tender or itchy rash or chilblains on the toes or foot)	○	○	○	○	○
Cracked or dry lips	○	○	○	○	○
Dental problems (i.e. chipped tooth, tooth loss)	○	○	○	○	○
Diarrhea	○	○	○	○	○
Difficulty concentrating or focusing	○	○	○	○	○
Difficulty sleeping	○	○	○	○	○
Difficulty speaking properly	○	○	○	○	○
Discoloration of the skin (for example: purple or blue on the hands or feet, no blistering)	○	○	○	○	○
Dizziness	○	○	○	○	○
Dry eyes	○	○	○	○	○
Dry or peeling skin	○	○	○	○	○
Dry scalp or dandruff	○	○	○	○	○
Dry throat	○	○	○	○	○
Ear pain/earache	○	○	○	○	○
Elevated thyroid hormones	○	○	○	○	○
Extreme pressure at base of head or occipital nerve	○	○	○	○	○
Eye stye or infection	○	○	○	○	○
Fatigue	○	○	○	○	○
Feeling irritable	○	○	○	○	○
Feeling of burning skin	○	○	○	○	○
Fever or chills	○	○	○	○	○
Floaters or flashes of light in vision	○	○	○	○	○
Foot pain	○	○	○	○	○
GERD (acid reflux) with excessive salivation	○	○	○	○	○
Goiter or lump in throat	○	○	○	○	○
Hair loss	○	○	○	○	○
Hand or wrist pain	○	○	○	○	○
Headache	○	○	○	○	○
Heart palpitations (heart skipping a beat or racing)	○	○	○	○	○
Heat intolerance	○	○	○	○	○

High blood pressure	○	○	○	○	○
Hormone imbalances	○	○	○	○	○
"Hot" blood rush	○	○	○	○	○
Inability to cry	○	○	○	○	○
Inability to exercise or be active	○	○	○	○	○
Inability to yawn	○	○	○	○	○
Internal tremors or buzzing/vibration	○	○	○	○	○
Irregular or skipped menstrual cycles	○	○	○	○	○
Jaw pain	○	○	○	○	○
Joint pain	○	○	○	○	○
Kidney issues or protein in urine	○	○	○	○	○
Kidney pain	○	○	○	○	○
Low blood oxygen	○	○	○	○	○
Low blood pressure	○	○	○	○	○
Lower back pain	○	○	○	○	○
Loss of appetite	○	○	○	○	○
Loss of hearing	○	○	○	○	○
Loss or decrease in quality of vision	○	○	○	○	○
Lump in throat/difficulty swallowing	○	○	○	○	○
Memory problems	○	○	○	○	○
Menstrual cycles that are heavier or lighter than normal	○	○	○	○	○
Mid-back pain at base of ribs	○	○	○	○	○
Mouth sores or sore tongue	○	○	○	○	○
Muscle or body aches	○	○	○	○	○
Muscle twitching	○	○	○	○	○
Nausea or vomiting	○	○	○	○	○
Neck muscle pain	○	○	○	○	○
Nerve pain	○	○	○	○	○
Nerve sensations (tingling, pins and needles, numbness)	○	○	○	○	○
Neuropathy in feet and hands (weakness, numbness, and pain)	○	○	○	○	○
New allergies	○	○	○	○	○
Night sweats	○	○	○	○	○
Nightmares	○	○	○	○	○
Painful scalp	○	○	○	○	○

Partial or complete loss of sense of smell	○	○	○	○	○
Partial or complete loss of sense of taste	○	○	○	○	○
Persistent chest pain or pressure	○	○	○	○	○
Personality change (drastic)	○	○	○	○	○
Petechiae (pinpoint rash)	○	○	○	○	○
Phantom smells	○	○	○	○	○
Phlegm in back of throat	○	○	○	○	○
Post-exertional malaise (worsened symptoms or flu-like symptoms after exertion)	○	○	○	○	○
Postnasal drip	○	○	○	○	○
Rash	○	○	○	○	○
Reflux or heartburn	○	○	○	○	○
Runny nose	○	○	○	○	○
Sadness	○	○	○	○	○
Seizures	○	○	○	○	○
Sharp or sudden chest pain	○	○	○	○	○
Shortness of breath or difficulty breathing	○	○	○	○	○
Shortness of breath or exhaustion from bending over	○	○	○	○	○
Sleeping more than normal	○	○	○	○	○
Sore throat	○	○	○	○	○
Spinal issues	○	○	○	○	○
Spikes in blood pressure	○	○	○	○	○
Swollen hands or feet	○	○	○	○	○
Swollen lymph nodes	○	○	○	○	○
Syncope (fainting)	○	○	○	○	○
Tachycardia (rapid heartbeat) at rest	○	○	○	○	○
Tachycardia (rapid heartbeat) after standing up	○	○	○	○	○
Thrush (white fungal infection in the mouth or throat)	○	○	○	○	○
Tinnitus or humming in ears	○	○	○	○	○
Tremors or shakiness	○	○	○	○	○
Upper back pain	○	○	○	○	○
UTI (urinary tract infection)	○	○	○	○	○
Weakened neck	○	○	○	○	○

Weight gain	◯	◯	◯	◯	◯

Are there any symptoms not listed above that you have experienced within the past 6 weeks?

*Please list all if so

____

During the past 6 weeks

	76-100% of health before COVID-19	51-75% of health before COVID-19	26-50% of health before COVID-19	0-25% of health before COVID-19
On your best day, would you say that you are at	◯	◯	◯	◯
On your worst day, would you say that you are at	◯	◯	◯	◯

Compared to your health before you received the first dose of your vaccine on [vaccine_arm_1][v_v_vax_date], would you say that your health is currently

◯ Better
◯ Worse
◯ The same
◯ Don't know

Please, in the space below, share any information about your experience with COVID-19 or the vaccine that we might have missed.

____

Ineligible

Please complete the survey below for all ineligible participants.

Find ineligible participants by going to:

Alerts & NotificationsClick on Alert #7 (Ineligible)Click on "view list" in "Activity" (below the alert)Select any ineligible participants who haven't yet been marked as ineligible.Thank you!

2846) Please check if this participant is ineligible ◯ This participant is ineligible

Lost to Follow-Up

Please complete the survey below for all participants who were lost to follow-up.

Participants are lost to follow-up if they:

Don't complete phone consultation after 3 attempts.Don't get vaccinated within 5 weeks of completing Study Visit 1 (unless they have extenuating medical circumstances).Are more than 1 week late for a study visit. (i.e. do not complete study visit 2 within 8 weeks after the first dose of the vaccine. The time period for study visit 2 should be 5-7 weeks after the vaccine.)Thank you!

2847) Please check if this participant is lost to follow-up ◯ This participant has been lost to follow-up

Off Study (Complete)

Please complete the survey below as soon as a participant has completed his or her last study visit.

Thank you!

2848) Please check if this participant has completed the study.

◯ This participant has completed the study
