# Supplementary material for: Impact of COVID-19 vaccination on symptoms and immune phenotypes in vaccine-naïve individuals with Long COVID
Source: Commun Med (Lond). 2025 May 9;5:163. doi: 10.1038/s43856-025-00829-3 (PMC12064684; doi:10.1038/s43856-025-00829-3)
Supplement: Supplementary file 3 — Description of Supplementary Data [file 43856_2025_829_MOESM3_ESM.docx]

**Description of additional supplementary data**

Supplementary Data 1: On the Physical Effect Scale, Number of Participants whose Symptoms Resolved, Improved, Stayed the Same, or Worsened after Vaccination Compared to Before Vaccination for the Fifteen Most Frequently Reported Symptoms

Supplementary Data 2: On the Social Effect Scale, Number of Participants whose Symptoms Resolved, Improved, Stayed the Same, or Worsened after Vaccination Compared to Before Vaccination

Supplementary Data 3: Wilcoxon Matched-Pairs Signed Rank Test Results for Humoral Responses to SARS-CoV-2 and Other Viral Pathogens (REAP Assay)

Supplementary Data 4: Coefficient values of Spearman correlation analyses for SARS-CoV-2 specific TCRs and antibody levels.

Supplementary Data 5: Unadjusted p-values of Spearman correlation analyses of SARS-CoV-2 specific TCRs and antibody levels.

Supplementary Data 6: The p-values of Spearman correlation analyses of SARS-CoV-2 specific TCRs and antibody levels multiplied by the number of tests.

Supplementary Data 7: Data Used to Generate Figure 1

Supplementary Data 8: Data Used to Generate Figure 2

Supplementary Data 9: Data Used to Generate Figure 3

Supplementary Data 10: Data Used to Generate Figure 4
