# Supplementary material for: Impact of COVID-19 vaccination on symptoms and immune phenotypes in vaccine-naïve individuals with Long COVID
Source: Commun Med (Lond). 2025 May 9;5:163. doi: 10.1038/s43856-025-00829-3 (PMC12064684; doi:10.1038/s43856-025-00829-3)
Supplement: Supplementary file 14 — Reporting Summary [file 43856_2025_829_MOESM14_ESM.pdf]

nature portfolio

Corresponding author(s): Akiko Iwasaki

Last updated by author(s): 10/18/2024

Reporting Summary

Nature Portfolio wishes to improve the reproducibility of the work that we publish. This form provides structure for consistency and transparency in reporting. For further information on Nature Portfolio policies, see our Editorial Policies and the Editorial Policy Checklist.

Statistics

For all statistical analyses, confirm that the following items are present in the figure legend, table legend, main text, or Methods section.

n/a	Confirmed	
☐	☒	The exact sample size (*n*) for each experimental group/condition, given as a discrete number and unit of measurement
☐	☒	A statement on whether measurements were taken from distinct samples or whether the same sample was measured repeatedly
☐	☒	The statistical test(s) used AND whether they are one- or two-sided<br>*Only common tests should be described solely by name; describe more complex techniques in the Methods section.*
☐	☒	A description of all covariates tested
☐	☒	A description of any assumptions or corrections, such as tests of normality and adjustment for multiple comparisons
☐	☒	A full description of the statistical parameters including central tendency (e.g. means) or other basic estimates (e.g. regression coefficient) AND variation (e.g. standard deviation) or associated estimates of uncertainty (e.g. confidence intervals)
☐	☒	For null hypothesis testing, the test statistic (e.g. F, t, r) with confidence intervals, effect sizes, degrees of freedom and P value noted<br>*Give P values as exact values whenever suitable.*
☒	☐	For Bayesian analysis, information on the choice of priors and Markov chain Monte Carlo settings
☐	☒	For hierarchical and complex designs, identification of the appropriate level for tests and full reporting of outcomes
☒	☐	Estimates of effect sizes (e.g. Cohen's *d*, Pearson's *r*), indicating how they were calculated

Our web collection on statistics for biologists contains articles on many of the points above.

Software and code

Policy information about availability of computer code

Data collection

Participant surveys were administered through RedCap version 12.0.25 (Vanderbilt University). Whole blood was collected in sodium-heparin-coated vacutainers (BD 367874, BD Biosciences) and EDTA-coated vacutainers (BD 367856, BD Biosciences).

Data analysis

Data analyses included simple descriptive statistics (median, range, frequencies, proportions) of participant demographics, experience with acute COVID-19 infection and Long COVID symptom profile before and after vaccination.

Analysis of biospecimen samples includes:

1. differences in SARS-CoV-2 specific T-cell responses, anti-SARS-CoV-2 antibody responses measured by ELISA and REAP before and after vaccination assessed using Wilcoxon matched-pairs signed rank tests,

2. correlation between observed T-cell responses and antibody levels as well as to determine concordance between the two different methods of determining anti-SARS-CoV-2 antibody levels, Spearman rank correlations were calculated, and the correlation coefficients between assays were used to measure distances [1-absolute (correlation coefficients)], and hierarchical clustering was conducted using Morpheus,

3. differences in SARS-CoV-2 T-cell responses, antibody levels, anti-viral antibody levels against common viruses and autoantibody levels among symptom outcome groups were compared using Kruskal-Wallis tests,

4. to estimate the average differences in expression of each cytokine over the course of vaccination we used linear mixed models via Restricted Maximum Likelihood (REML) regression, estimating the cytokine expression over all three timepoints amongst three symptom

outcome groups; the model incorporated a random effect for each individual as a random intercept, nested within their respective symptom outcome groups. The fixed effects in the model included the symptom outcome and time, along with an interaction term between them to investigate any potential modifying effect of time on the symptom outcome group,

5. unsupervised hierarchical clustering was conducted on 162 plasma-derived analytes obtained from the multiplex proteomic assays to assess patterns of expression across the cohort, and data was standardized by factor and clustering was done based on Ward's distance,

6. to identify predictors of symptom improvement from the 162 plasma-derived analytes, we used Partial Least Squares (PLS) analysis via the Non-linear iterative partial least squares (NIPALS) algorithm with k-fold cross validation (k=5); all plasma factors and sex were incorporated into the model; final analysis involved reduction to 4 principal components, which simultaneously minimized the Van der Voet's T-squared statistic (0.00, P=1.00) and the Root Mean PRESS (0.27) accounting for a sizeable portion of the variance in the data (cumulative pseudo-R-squared= 0.98); post-analysis, the Variable Importance on Projection (VIP) score was generated for each feature and bootstrapped using Bayesian Bootstrapping; bias-corrected 95% confidence intervals were calculated; only features with 95% confidence intervals above the threshold cutoff of 0.8, corresponding to the standard threshold for importance were considered significant.

Data analyses were performed using R (v 4.2.2), GraphPad PRISM(v 9.5.1), and JMP statistical software platform (JMP® Pro 17.0.0).

For manuscripts utilizing custom algorithms or software that are central to the research but not yet described in published literature, software must be made available to editors and reviewers. We strongly encourage code deposition in a community repository (e.g. GitHub). See the Nature Portfolio guidelines for submitting code & software for further information.

Data

Policy information about availability of data

All manuscripts must include a data availability statement. This statement should provide the following information, where applicable:

- Accession codes, unique identifiers, or web links for publicly available datasets
- A description of any restrictions on data availability
- For clinical datasets or third party data, please ensure that the statement adheres to our policy

The data that support the findings of this study are available from the corresponding author upon reasonable request. Data are located in controlled access data storage at the Yale School of Medicine.

Human research participants

Policy information about studies involving human research participants and Sex and Gender in Research.

Reporting on sex and gender	Gender was self-reported by participants. Analyses by gender were planned as we sought to enroll 50-100 participants, but analyses by gender were ultimately not performed due to small sample size. 13 participants self-reported female gender; 3 reported male gender.
Population characteristics	The median age of the 16 included participants was 54 years (range 21-69), 13 (81%) were female (3 were male), and 14 (88%) identified as Non-Hispanic White (n=1 American Indian/Alaska Native, n=1 Hispanic). At baseline, on participants' worst days, 9 (56%) felt they were 50% or less of their health before COVID-19. On participants' best days, 7 (44%) reported feeling 51-75% of their health before COVID-19. The median number of symptoms per participant before vaccination was 23 (Q1-Q3, 13.8-27). Three (19%) participants were previously hospitalized due to COVID-19 and 4 (25%) visited the hospital or were hospitalized for COVID-19 more than 2 weeks after onset of acute disease.
Recruitment	Recruitment was conducted through social media advertisements and patient support groups. Participants also had to be willing to travel to New Haven, Connecticut to provide blood and saliva samples.
Ethics oversight	This study was approved by the Yale University Institutional Review Board (IRB #2000030423)

Note that full information on the approval of the study protocol must also be provided in the manuscript.

Field-specific reporting

Please select the one below that is the best fit for your research. If you are not sure, read the appropriate sections before making your selection.

☒ Life sciences ☐ Behavioural & social sciences ☐ Ecological, evolutionary & environmental sciences

For a reference copy of the document with all sections, see nature.com/documents/nr-reporting-summary-flat.pdf

Life sciences study design

All studies must disclose on these points even when the disclosure is negative.

Sample size	We sought to enroll 50-100 participants to estimate the overall health change of individuals with Long COVID after receiving a COVID-19 vaccine compared to their overall health pre-vaccination. However, the study was terminated early due to an inability to reach the target sample size given that few people with Long COVID were vaccine naïve.

Data exclusions	To harmonize data across the two batches, ComBat was used, an empirical Bayes method available through the "sva"34 R package (version 3.4.6), designating the initial batch as the reference and incorporating the following covariates: disease status, sex, age, and hormone conditions. The effectiveness of the ComBat was validated using sample replicates between each batch in a matched pairs analysis. Analytes that exhibited significant differences post-correction were excluded from further analysis
Replication	*Describe the measures taken to verify the reproducibility of the experimental findings. If all attempts at replication were successful, confirm this OR if there are any findings that were not replicated or cannot be reproduced, note this and describe why.*
Randomization	Randomization is not relevant to this study, as this is a descriptive analysis of an observational single-treatment intervention. Confounder control via covariate adjustment is not relevant to this study.
Blinding	Given the study was a single-treatment intervention, blinding of treatment is not relevant. Participants had to express their willingness to be vaccinated to be enrolled, and only participants who obtained a vaccination were included in the analysis.

Reporting for specific materials, systems and methods

We require information from authors about some types of materials, experimental systems and methods used in many studies. Here, indicate whether each material, system or method listed is relevant to your study. If you are not sure if a list item applies to your research, read the appropriate section before selecting a response.

Materials & experimental systems

n/a	Involved in the study	
☐	☒	Antibodies
☒	☐	Eukaryotic cell lines
☒	☐	Palaeontology and archaeology
☒	☐	Animals and other organisms
☒	☐	Clinical data
☒	☐	Dual use research of concern

Methods

n/a	Involved in the study	
☒	☐	ChIP-seq
☒	☐	Flow cytometry
☒	☐	MRI-based neuroimaging

Antibodies

Antibodies used	Human anti-spike (SARS-CoV-2 human anti-spike [AM006415; 91351, Active Motif]) , human anti-nucleocapsid (SARS-CoV-2 anti-nucleocapsid [1A6; MA5-35941, Active Motif]) and HRP anti-human IgG antibody (A00166, GenScript)
Validation	1. SARS-CoV-2 human anti-spike [AM006415; 91351, Active Motif: Wan J., et. al. Human IgG neutralizing monoclonal antibodies block SARS-CoV-2 infection. Cell Reports, 32: 107918, July 21, 2020.<br>2. SARS-CoV-2 anti-nucleocapsid [1A6; MA5-35941, Active Motif]: https://www.thermofisher.com/antibody/product/SARS-CoV-2-Nucleocapsid-Chimeric-Antibody-clone-1A6-Recombinant-Monoclonal/MA5-35941<br>3. HRP anti-human IgG antibody (A00166, GenScript): Carolina Lucas, et al. Impact of circulating SARS-CoV-2 variants on mRNA vaccine-induced immunity. Nature. (2021-10)
